# Identification of miRNAs-mediated seed and stone-hardening regulatory networks and their signal pathway of GA-induced seedless berries in grapevine (*V. vinifera* L.)

**DOI:** 10.1186/s12870-021-03188-y

**Published:** 2021-09-29

**Authors:** Peipei Wang, Xuxian Xuan, Ziwen Su, Wenran Wang, Mostafa Abdelrahman, Songtao Jiu, Xiaowen Zhang, Zhongjie Liu, Xicheng Wang, Chen Wang, Jinggui Fang

**Affiliations:** 1 College of Horticulture, Nanjing Agricultural University, Nanjing, 210095 China; 2 Institute of Pomology, Jiangsu Academy of Agricultural Sciences, Nanjing, 210014 China; 3 Department of Botany, Faculty of Sciences, Aswan University, Aswan, 81528 Egypt; 4 Arid Land Research Center, Tottori University, Tottori, 680-001 Japan; 5 Department of Plant Science, School of Agriculture and Biology, Shanghai Jiao Tong University, Shanghai, China

**Keywords:** Grape, Seed and stone hardening development, GA, Seedless berry, miRNA, Target genes

## Abstract

**Background:**

Stone-hardening stage is crucial to the development of grape seed and berry quality. A significant body of evidence supports the important roles of MicroRNAs in grape-berry development, but their specific molecular functions during grape stone-hardening stage remain unclear.

**Results:**

Here, a total of 161 conserved and 85 species-specific miRNAs/miRNAs* (precursor) were identified in grape berries at stone-hardening stage using Solexa sequencing. Amongst them, 30 VvmiRNAs were stone-hardening stage-specific, whereas 52 exhibited differential expression profiles during berry development, potentially participating in the modulation of berry development as verified by their expression patterns. GO and KEGG pathway analysis showed that 13 VvmiRNAs might be involved in the regulation of embryo development, another 11 in lignin and cellulose biosynthesis, and also 28 in the modulation of hormone signaling, sugar, and proline metabolism. Furthermore, the target genes for 4 novel VvmiRNAs related to berry development were validated using RNA Ligase-Mediated (RLM)-RACE and Poly(A) Polymerase-Mediated (PPM)-RACE methods, and their cleavage mainly occurred at the 9th–11th sites from the 5′ ends of miRNAs at their binding regions. In view of the regulatory roles of GA in seed embryo development and stone-hardening in grape, we investigated the expression modes of VvmiRNAs and their target genes during GA-induced grape seedless-berry development, and we validated that GA induced the expression of VvmiR31-3p and VvmiR8-5p to negatively regulate the expression levels of *CAFFEOYL COENZYME A-3-O-METHYLTRANSFERASE* (*VvCCoAOMT*), and *DDB1-CUL4 ASSOCIATED FACTOR1* (*VvDCAF1*). The series of changes might repress grape stone hardening and embryo development, which might be a potential key molecular mechanism in GA-induced grape seedless-berry development. Finally, a schematic model of miRNA-mediated grape seed and stone-hardening development was proposed.

**Conclusion:**

This work identified 30 stone-hardening stage-specific VvmiRNAs and 52 significant differential expression ones, and preliminary interpreted the potential molecular mechanism of GA-induced grape parthenocarpy. GA negatively manipulate the expression of *VvCCoAOMT* and *VvDCAF1* by up-regulation the expression of VvmiR31-3p and VvmiR8-5p, thereby repressing seed stone and embryo development to produce grape seedless berries.

**Supplementary Information:**

The online version contains supplementary material available at 10.1186/s12870-021-03188-y.

## Background

Grape (*V. vinifera* L.) is a soft pulpy berry with a thin edible outer skin (exocarp) and fleshy edible inner layers (mesocarp and endocarp) of storage tissues containing seeds. During grape stone-hardening stage, the coats surrounding the seed gradually harden to form a lignified seed coat (stone). Stone hardening is an essential strategy for seed protection and dispersal in different plant species, including cherry (*Prunuscerasus* and *P. avium*), peach (*P. persica*), plum (*P. salicina*) and grape [1–3]. Lignin deposition plays a critical role in seed stone-hardening formation, and several transcriptome and proteome studies have demonstrated that the flavonoid and lignin biosynthesis pathways are highly involved in the lignification of seed-coat structures [4–7]. However, seed coat development might affect the expansion and ripening of fruits, resulting in low-quality fruit [7]. By contrast, seed abortion and seed-coat degradation during the seed-development stage could lead to seedless berry, which is a favourable trait for consumers [8–10]. Therefore, an in-depth understanding of the regulatory mechanisms and molecular basis underlying seed stone-hardening formation during grape-berry development is essential for the production of high-quality fruit.

With the recent development of high-throughput sequencing technologies, many miRNAs from various plant species have been released on the miRBase21.0 database (http://www.mirbase.org). The miRNA-guided cleavage of target mRNAs and/or the translational repression of development-related genes especially transcription factors (TFs) in different plant species, including grapevine plays an important role in the regulation of fruit development and ripening [11–13]. For example, PbrmiR397a regulates the fruit stone-cell lignification by inhibiting the expression of three *LACCASE* (*LAC*) genes involved in lignin biosynthesis, resulting in decreased lignin content and stone-cell number in Chinese pear (*Pyrus bretschneideri*) fruit [7]. *Arabidopsis* AUXIN RESPONSE FACTOR8 (*AtARF8*) and FRUITFULL (*AtFUL*) MADS-domains act together to directly activate the expression of *MIR172C*, a valve-specific AtmiR172-encoding gene, leading to the repression of the flower-patterning gene *APETALA2* (*AtAP2*) and the promotion of fruit valve growth [14]. Similarly, tomato (*S. lycopersicum*) SlmiR156/157 and SlmiR172 have been reported as important regulators in the ripening process of tomato fruits by inhibiting the expression of ripening regulatory genes *COLORLESS NON-RIPENING* (*CNR*) and *SlAP2a* [15]. Our research group also identified and characterized many known and grape specific miRNAs (VvmiRNAs) during grape-berry development, of which a large number of VvmiRNAs might be involved in the modulation of berry development and seed formation [12, 13, 16–19], and a good example is that VvmiR058 negatively regulates the expression of *POLYPHENOL OXIDASE* (*VvPPO*) gene involved in the synthesis of lignin in peel and seeds during berry development [8]. Nevertheless, the research on the regulatory networks and transcriptome dynamics of VvmiRNAs during seed and stone-hardening development in grape remains imperative.

Gibberellin (GA) is a well-known phytohormone involved in diverse biological processes of grape-berry development, leading to the improvement of berry size, weight, and seedless-berry formation [13, 20]. Many VvmiRNAs are differentially expressed during the different stages of grape-berry development in response to GA application [21]. Consequently, the seed stone-hardening stage-specific VvmiRNAs responsive to GA signal could be further identified, which is crucial to the molecular breeding of seedless-grape production. Hence, in the present study, we attempted to identify and characterize the expression of seed stone-hardening stage-specific VvmiRNAs and their target genes using Solexa sequencing technology. Moreover, GO and KEGG pathway analyses were performed to explore the regulatory networks of VvmiRNA-mediated seed stone-hardening development stage in grape berries. The dynamic regulatory roles of several important miRNA-mediated target genes involved in the GA signal pathway and seed development in GA-induced grape seedless berries were also explored. Our results can provide novel genetic information for the improvement of grape breeding programs to advance the production of new seedless-grape varieties.

## Results

### High throughput sequencing of small RNA in grape berries of the stone-hardening stage

**Fig. 1:**
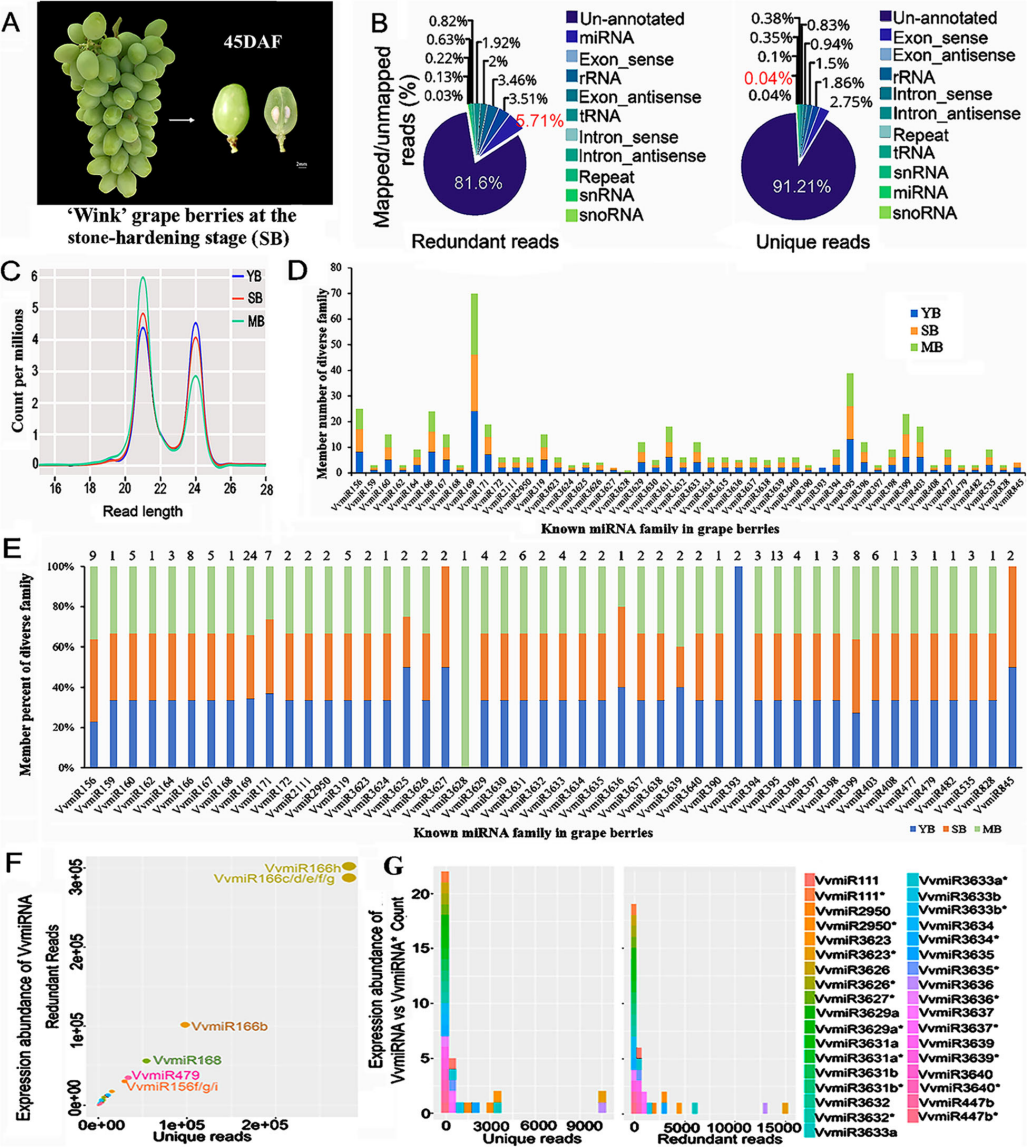
The sRNA dataset of grape berries at 45DAF stage. **a** Morphology of grape berries at the stone-hardening stage (SB). **b** Pie charts of diverse types of sRNAs derived from grape berry library at seed coat-hardening stage based on the redundant and unique reads, respectively. **c** Expression level of miRNA (21 nt) and siRNA (24 nt) reads at different berry developmental stages. **d** Percentage number of VvmiRNA family members at various developmental stages. **e** Number of diverse VvmiRNA family members at different berry developmental stages. **f-g** Expression abundance of redundant vs unique reads in VvmiRNA and VvmiRNA versus VvmiRNA* precursor. Young berry, YB [5 days after flowering (5DAF)]; stone-hardening berry, SB (45DAF); ripening/mature berry, MB (90DAF)

The sRNA library from the ‘Wink’ cultivar grape stone-hardening berries (SB) at 45 days after flowering (DAF) was constructed and sequenced in depth by Solexa technology to explore the role of sRNA in the grape stone-hardening process (Fig. 1a). Sequencing result analysis showed that total of 11,456,656 redundant and 3,591,857 unique clean reads were identified (Supplementary Table S1), of which about 18.40 and 8.79% were annotated, respectively. Out of them, 5.54 and 1.99% of these two reads, respectively, were further successfully mapped into the non-coding RNA of rRNAs, snRNAs, snoRNAs, and tRNAs. While, 5.71 and 0.04% of the redundant and unique reads, respectively, were determined to be putative known miRNAs, and the majority of the redundant (81.6%) and unique (91.21%) reads, were mapped to un-annotated regions in the grape genome (Fig. 1b).

The length distribution of sRNAs in the SB library were uneven, and most sRNA reads were 21 and 24 nucleotides (nt), which were the characteristic lengths of miRNAs and siRNAs, respectively, and consistent with the expected size range generated by Dicer [22]. In contrast to two other sRNA libraries of 5 DAF berry (YB) and 90 DAF berry (MB) (data were not shown), the sRNA distribution at 21 and 24 nt at SB was more similar to that at MB than that at YB (Fig. 1c). The comparative expression level using normalized counts per million of miRNA (21 nt) and siRNA (24 nt) at grape YB, SB, and MB stages indicated a gradual increase in the known miRNA (21 nt) reads towards the MB stage. By contrast, the siRNA (24 nt) reads exhibited a reverse trend with the highest level observed at the early YB stage followed by a gradual decrease towards the MB stage (Fig. 1c). The above results suggested that known miRNA (21 nt) reads were highly abundant during the later stage (MB) of grape-berry ripening compared with siRNA (24 nt) reads.

### Identification and characterization of known VvmiRNAs in the stone-hardening stage of grape berries

A total of 143 known VvmiRNAs and 18 corresponding precursors, namely, VvmiRNAs* belonging to 48 VvmiRNA families, were identified in the stone-hardening stage of grape berries (Supplementary Table S2). Although the number of known VvmiRNA members within each family varied from 1 to 24 during grape-berry development, they had the most variation at the SB stage compared with the other two stages (YB and MB) (Fig. 1d and Fig. 1e). Amongst 48 VvmiRNA families, VvmiR169 family was highly represented during all three stages with 22–24 members, followed by VvmiR395 family with 13 members, VvmiR156 family with 8–9 members, and VvmiR166 family with 8 members, whereas the remaining miRNA families consisted of 1–7 members (Fig. 1d). This result suggested a diversification in the functions of these VvmiRNA families during grape development and ripening. Interestingly, from the percent numbers of VvmiRNA members at different grape-berry developments, we found that VvmiR3628 family was sequenced only in mature berries (90 DAF), whereas VvmiR393 family was detected only in young berries (5 DAF) (Fig. 1e), indicating the stage-specificity of VvmiRNAs’ expression.

We further characterized most known VvmiRNA families with high expression abundances at the stone-hardening stage of grape berries. For instance, amongst the 143 known VvmiRNAs, about 68% exhibited high copy read number, including 42 known VvmiRNAs with read number > 1000, whereas 55 known VvmiRNAs had read numbers ranging between 100 and 1000 (Supplementary Table S2). Specifically, VvmiR166, VvmiR168, VvmiR479, VvmiR156, and VvmiR3636 families (not including their VvmiRNA* sequences) possessed more than 10,000 copy reads (Supplementary Table S2). Meanwhile, 83.3% of known VvmiRNA* family members exhibited lower copy number than their corresponding VvmiRNAs (Fig. 1f and g, and Supplementary Table S2), which may have been due to the fact VvmiRNAs* were easier to degrade than their corresponding VvmiRNAs. Thus, they usually had less copy numbers, similar to a previous report [23]. Nevertheless, two VvmiRNA* families of VvmiR3623* and VvmiR2950* showed higher copy numbers (14,830 and 3029, respectively) than their corresponding mature sequences (4405 and 1773, respectively) (Fig. 1g and Supplementary Table S2). This finding implied that VvmiR3623* and VvmiR2950* might play potential roles during the development and ripening of grape berries, similar to VvmiRNAs [17, 24].

### Screening of novel VvmiRNAs at grape stone-hardening stage and their validation using miR-RACE and qRT-PCR

**Fig. 2:**
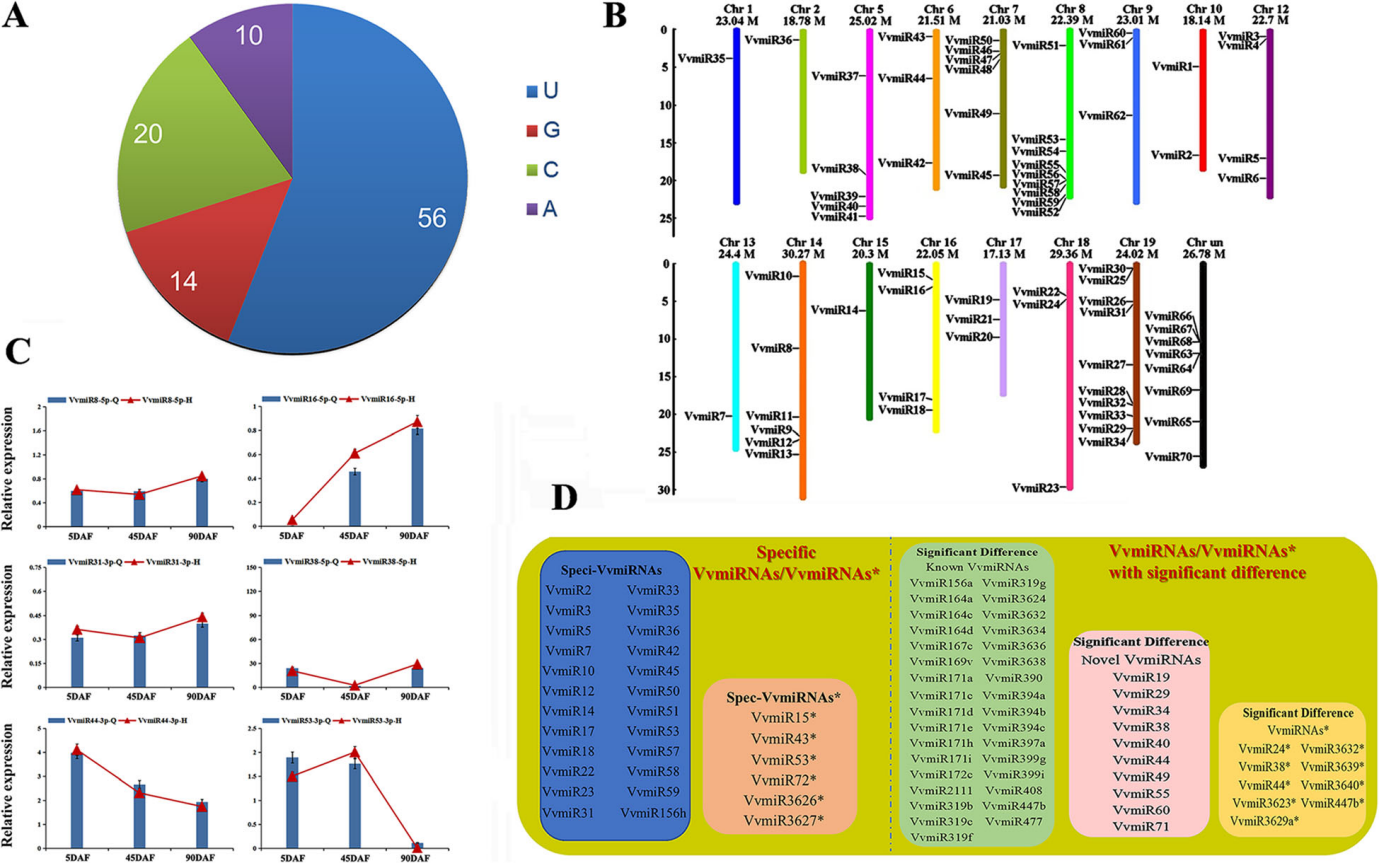
Screening of novel VvmiRNAs at grape stone-hardening stage. **a** First base preference. **b** Distribution in grape chromosomes of VvmiRNAs. **c** Validation of VvmiRNAs from high throughput sequencing data by qRT-PCR at different grape berry developmental time points [YB (5DAF), SB (45DAF), MB (90DAF)]. Each bar indicated the mean ± SE of triplicate assays. H: high throughput sequencing; Q: qRT-PCR; each assay was conducted by using three repeats. There is no significant difference between high throughput sequencing and qRT-PCR results in *P* < 0.05 by Student’s *t*-test. **d** Comparison of specific and significant VvmiRNAs in grape sRNA library. VvmiRNAs with specific expressions at the seed coat-hardening stage, Spec-VvmiRNAs: VvmiRNAs with the absolute log_2_Fold (YB/SB) or log_2_Fold (MB/SB) > 1, Significant difference VvmiRNAs

According to the annotation criteria of novel miRNAs [25], all un-annotated sRNAs were used to explore the stem–loop structures of their precursors for the prediction of novel miRNAs. A total of 90,352 reads were identified as novel VvmiRNAs, including 72 novel VvmiRNAs and 12 novel VvmiRNAs*, in the grape stone-hardening stage (Table 1). These novel precursors were folded into stable hairpin structures, and their negative minimal folding free energy (MFE) ranged from − 108.2 kcal mol^− 1^ to − 20.88 kcal mol^− 1^ (Table 1), which was in line with the criteria of novel VvmiRNAs (MFE < − 20.0 kcal mol^− 1^) as previously reported [25]. The novel VvmiRNAs and VvmiRNAs* were primarily 21 nt in length, accounting for 84.14% (69/82), and the first base with uracil (U) at the 5′-end of their mature sequences reached 56.0%, confirming that these were novel VvmiRNAs (Fig. 2a). From our datasets, the novel VvmiRNAs were unconserved, species specific, and low abundance; they usually exhibited lower accumulation level than conserved ones, and in agreement with previous results [23, 26, 27]. Interestingly, we also observed a few novel VvmiRNAs with high abundance and read number > 1000, such as VvmiR10, VvmiR13, VvmiR29, VvmiR30, VvmiR34, VvmiR37, VvmiR43, and VvmiR71 (Table 1). Amongst them, VvmiR37, VvmiR13, and VvmiR30 showed higher copy numbers (37,985, 13,748, and 10,199, respectively) than the others (Table 1). Furthermore, their corresponding VvmiR37* and VvmiR13* exhibited high copy numbers with 2588 and 1332, respectively, which implying that VvmiR37/VvmiR37* and VvmiR13/VvmiR13* may play significant roles during the stone-hardening stage of grape berries. The location of novel VvmiRNAs in their precursors showed that 40 novel VvmiRNAs were located in the 5′-arm of their precursors, whereas another 32 ones were located in the 3′-arm of their precursors (Table 1). Similarly, seven novel VvmiRNAs* were located in the 5′-arm of their precursors, whereas another 5 ones were located in the 3′-arm of their precursors (Table 1). These results indicated that the 5′-arm of miRNA precursors may be more efficient in generating miRNAs and miRNA* than the 3′-arm. However, further confirmation of this mechanism is necessary. The identified novel VvmiRNAs exhibited three types of VvmiRNA-3P or VvmiRNA-5P or the first sequences (3p and 5p) (Table 1). As shown in Table 1, the miRNA with 3p indicated this miRNA sequence originated only from the 3′ arm of its precursor, and the marked 5p denoting the corresponding miRNA originated just from the 5′ arm of its precursors. Conversely, the miRNA with 3p and 5p represented both arms of miRNA precursors that generated two sequences of miRNA and miRNA* (Table 1). The distribution of all these novel VvmiRNAs varied between the 19 grape Chrs and 1 unknown Chr (Fig. 2b). Amongst them, Chr19 possessed the highest number (10) of novel VvmiRNAs, followed by Chr8 with 9 novel VvmiRNAs and the unknown Chr with 8 ones (Fig. 2b), whereas Chr3, Chr4, and Chr11 did not harbor any novel miRNAs (Fig. 2b).

**Table 1:** Novel miRNAs identified in grape berries of stone hardening stage

Id	location	mfe	Id(5p)	Id(3p)	count(5p)	count(3p)	seq(5p)
VvmiR1	chr10:4660933:4661043:+	−49.4	–	▲	–	15	–
VvmiR2	chr10:16154669:16154760:+	−54	▲	–	58	–	UCAGCGGCUGAGAUAAGCAAA
VvmiR3	chr12:836885:836996:-	−29.4	–	▲	–	6	–
VvmiR4	chr12:1137532:1137662:-	−32.3	–	▲	–	5	–
VvmiR5	chr12:17324634:17324749:-	−27.7	–	▲	–	5	–
VvmiR6	chr12:20076549:20076639:-	−26.3	–	▲	–	20	–
VvmiR7	chr13:20001280:20001472:+	−47.7	–	▲	–	10	–
VvmiR8	chr13:20934176:20934434:-	−64.1	▲	–	8	–	UCCAAGGAUGGAAAAGGCUUC
VvmiR9	chr13_random:2451486:2451802:-	−78.6	–	▲	–	7	–
VvmiR10	chr14:10959011:10959186:+	−20.55	▲	–	1447	–	CUAGAGAUUGUGGAUUAGGCU
VvmiR11*/VvmiR11	chr14:22335449:22335595:+	−70	△	▲	1	12	UUCUCAGCUACUAAUAUCAAG
VvmiR12	chr14:1713283:1713430:-	−46.7	▲	–	47	–	CACGGAAGUGGAGCGGGCGGGCG
VvmiR13/VvmiR13*	chr14:19755471:19755583:-	−57.9	▲	▲	13,748	1332	GGAAUGGGCUGAUUGGGAUA
VvmiR14	chr14:22697840:22697962:-	−37.7	▲	–	23	–	UCAGCUGGGUUGGCAUCUGAA
VvmiR15/VvmiR15*	chr14:24560621:24560731:-	−54.7	▲	△	13	3	UCUGAACUCUCUCCCUCAUGGC
VvmiR16	chr15:6122847:6123043:-	−50.53	▲	–	7	–	UCUUUUCUUGAUAGAAGGCCU
VvmiR17	chr16:2126040:2126238:-	−38.7	▲	–	77	–	AUACCAUGUGGAAAAGAGGAAUC
VvmiR18	chr16:3111476:3111566:-	−51.7	–	▲	–	5	–
VvmiR19	chr16:17808410:17808741:-	−79	▲	–	41	–	UGCGGGUGGAAGAGAAGGAAG
VvmiR20	chr16:19208159:19208370:-	−67.7	–	▲	–	11	–
VvmiR21	chr17:4716715:4716853:+	−44.23	–	▲	–	10	–
VvmiR22	chr17:9575775:9575974:+	−57.7	▲	–	5	–	CGACGGCAAGGACACUUUCGU
VvmiR23	chr17:7265156:7265271:-	−43.9	–	▲	–	19	–
VvmiR24*/VvmiR24	chr18:4079210:4079312:+	−71.9	△	▲	2	93	GACAAGUUACAUACAUCCAAG
VvmiR25	chr18:29129189:29129421:+	−68.2	▲	–	17	–	UCCUUCGGCGUCGGCAAAUCC
VvmiR26	chr18_random:4558402:4558602:+	−51.3	–	▲	–	9	–
VvmiR27/VvmiR27*	chr19:607159:607251:+	−21.75	▲	△	22	1	UUUGAUCAGAUAUUGGAUUGC
VvmiR28	chr19:5046231:5046495:+	−67.97	▲	–	17	–	CAGGACUGGCAGUGAUGGUUA
VvmiR29*/VvmiR29	chr19:13510105:13510195:+	−44.7	△	▲	1	8174	UCCCUCAAAGGCUUCCAAUUU
VvmiR30	chr19:18678400:18678570:+	−51.9	▲	–	10,199	–	GUUGGAAGUCGGUGGGGGAAC
VvmiR31	chr19:21910338:21910598:+	−54.19	–	▲	–	7	–
VvmiR32	chr19:580958:581064:-	−21	–	▲	–	28	–
VvmiR33	chr19:5446765:5447061:-	−68.9	–	▲	–	8	–
VvmiR34	chr19:18872600:18872761:-	−50	▲	–	1615	–	GUUGGAAGUCGGUGGGGGACC
VvmiR35	chr19:20383158:20383366:-	−66.7	▲	–	10	–	UGCUGAGUCAGUGAUGGUAGG
VvmiR36	chr19:22103176:22103318:-	−35.7	▲	–	11	–	UGGGCUUGUGGAGAAGAAAGUGA
VvmiR37/VvmiR37*	chr1:3865565:3865681:+	−46.2	▲	△	37,985	2588	CAUGGGCGGUUUGGUAAGAGG
VvmiR38*/VvmiR38	chr2:1237534:1237664:+	−64.9	△	▲	17	629	ACUCUCCCUCAAGGGCUUCUG
VvmiR39	chr5:6017515:6017763:-	−98.3	▲	–	10	–	CAGCAGUUGCUAUUGUGGUUG
VvmiR40	chr5:19124470:19124728:-	−65.6	–	▲	–	15	–
VvmiR41	chr5:22090345:22090434:-	−37.9	–	▲	–	9	–
VvmiR42	chr5:23402211:23402278:-	−27.1	▲	–	10	–	CUGAACAGAACUGAGGACAGU
VvmiR43*/VvmiR43	chr5:24742118:24742235:-	−45.5	△	▲	13	2793	UUUUGUUGCUGGUCAUCUAGUC
VvmiR44/VvnuR44*	chr6:17896119:17896283:+	−75.22	△	▲	61	38	UGCAUUUGCACCUGCACCUUA
VvmiR45	chr6:777459:777636:-	−30.5	–	▲	–	61	–
VvmiR46	chr6:6489357:6489602:-	−103.01	▲	–	22	–	CACUCCCUCGAGCUCGUCGGC
VvmiR47	chr7:19450050:19450214:+	−49.6	–	▲	–	10	–
VvmiR48	chr7:2818487:2818617:-	−40.23	–	▲	–	6	–
VvmiR49	chr7:3130346:3130460:-	−26.9	–	▲	–	15	–
VvmiR50	chr7:3926329:3926600:-	−92.68	–	▲	–	5	–
VvmiR51	chr7:11137979:11138169:-	−42.6	▲	–	12	–	UCUGACGUAUAUGCUGAUGGA
VvmiR52	chr7_random:1422270:1422383:-	−52.2	▲	–	45	–	UGACAAAGAGAGAGAGCACAC
VvmiR53*/VvmiR53	chr8:2139178:2139403:+	−108.2	△	▲	3	23	GGGUAGUAUGCUGCUGUCUU
VvmiR54	chr8:22308229:22308469:+	−68.25	▲	–	14	–	AUGUAUUUGAGGGAAAGCAAA
VvmiR55	chr8:14593879:14594080:-	−98.9	▲	–	87	–	CCGAGGGAGAGAGCGAGAGGA
VvmiR56	chr8:16138745:16139039:-	−84.2	–	▲	–	8	–
VvmiR57	chr8:18999725:18999889:-	−57.1	▲	–	24	–	UCUGCAUUUGCACCUGCACCU
VvmiR58	chr8:19904186:19904332:-	−42.5	▲	–	11	–	CACAUAUAAUUUUUUCCCGUCA
VvmiR59	chr8:19949160:19949307:-	−45.7	▲	–	11	–	CACAUAUAAUUUUUUCCCGUCA
VvmiR60	chr8:20492988:20493218:-	−60.2	–	▲	–	10	–
VvmiR61	chr8:21905817:21906095:-	−100	▲	–	159	–	CAUCGUCCGAGGCUAUGGCGG
VvmiR62	chr9:406257:406420:+	−73.5	▲	–	9	–	UCUGUUGGGACGUCAUUUGUU
VvmiR63	chr9:1040473:1040614:+	−48.5	▲	–	22	–	CCUUGGCUGUUGGAGAGGAUA
VvmiR64	chr9:11237291:11237395:-	−28.8	▲	–	25	–	UGAUAGAUUAAGGUACCUCAA
VvmiR65	chrUn:11785723:11785836:+	−51.3	▲	–	45	–	UGACAAAGAGAGAGAGCACAC
VvmiR66	chrUn:11820795:11820908:+	−50.1	▲	–	45	–	UGACAAAGAGAGAGAGCACAC
VvmiR67	chrUn:20798908:20799202:+	−69.53	–	▲	–	8	–
VvmiR68	chrUn:10241355:10241468:-	−52.2	▲	–	45	–	UGACAAAGAGAGAGAGCACAC
VvmiR69	chrUn:10268710:10268823:-	−52.2	▲	–	45	–	UGACAAAGAGAGAGAGCACAC
VvmiR70	chrUn:10281947:10282060:-	−51	▲	–	45	–	UGACAAAGAGAGAGAGCACAC
VvmiR71*/VvmiR71	chrUn:16672978:16673068:-	−45.5	△	▲	1	8174	UCCCUCAAAGGCUUCCAAUUU
VvmiR72	chrUn:25396495:25396608:-	−52.2	▲	–	45	–	UGACAAAGAGAGAGAGCACAC

▲ denotes VvmiRNA; △ represents VvmiRNA*; − indicates no

Subsequently, six novel VvmiRNAs closely related to berry development, such as VvmiR8, VvmiR16, VvmiR31, VvmiR38-5p, VvmiR44-3p, and VvmiR53-3p, were further validated by miR-RACE. Their precise sequences were detected by miR-RACE, and the sequences were consistent with those from the high-throughput sequencing dataset (Table 1 and Supplementary Table S2), which further verified the results of high throughput sequencing. Moreover, their qRT-PCR expression profiles during grape-berry development showed differential expression patterns similar to the high-throughput sequencing dataset (Fig. 2c). Therefore, our miR-RACE and qRT-PCR results confirmed the reliability and expression modes of VvmiRNA involved in the modulation of grape-berry development.

### Identification of grape stone-hardening stage-specific VvmiRNAs

The identification of grape stone-hardening stage-specific VvmiRNAs is essential to gain insights into the regulatory roles of grape-berry development. Compared with our other two sRNA libraries from 5 and 90 DAF in our other work (Supplementary Tables S3 and S4 and Fig. 2d), 35 VvmiRNAs/VvmiRNAs* were identified only at the stone-hardening stage of grape berries, including 28 VvmiRNAs (1 known and 27 novel) and 7 VvmiRNAs* (2 known and 5 novel). Among them, there were a large number of stage-specific novel VvmiRNAs indicated that novel miRNAs may play significant roles in the stone-hardening stage of grape-berry development.

To identify the differential expression VvmiRNAs during grape-berry development, the fold changes log2 (YB/SB) or log2 (MB/SB) > 1 cut offs were selected, and the filtered VvmiRNA/VvmiRNA* possessed significant expression difference across diverse development stages of grape berries. Here, we discovered that 52 VvmiRNAs/VvmiRNAs* exhibited significant differences in their expression levels during grape-berry development and ripening, comprising 44VvmiRNAs (34 known and 10 novel ones) and 8 VvmiRNAs* (5 known and 3 novel ones) (Supplementary Tables S3 and S4 and Fig. 2d). This finding indicated that they may possess dynamic regulatory roles of grape-berry development and of them, more known VvmiRNAs may have dynamic variation in their regulatory roles than novel ones.

### SNPs and their edit types of known VvmiRNAs/VvmiRNAs* from grape berries at stone-hardening stage

**Fig. 3:**
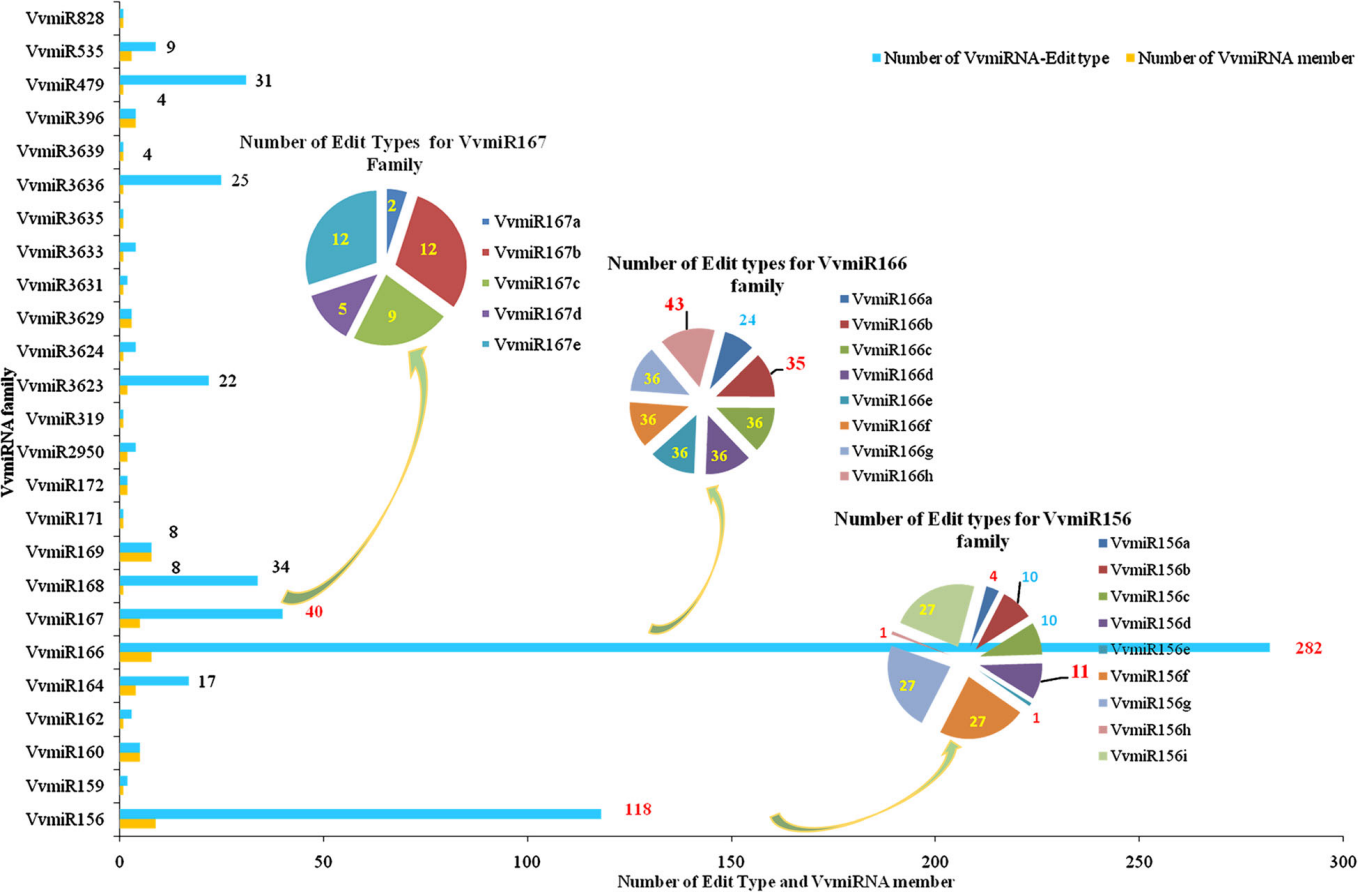
Number of SNP and their Edit types of various members of VvmiRNA families. Number in Pie denotes the number of different SNP edit types of VvmiRNA families

Numerous SNP variations of known VvmiRNAs/VvmiRNAs* and their Edit types were detected in our datasets (Fig. 3), which were consistent with our previous work in ‘Amur’ grape [23]. Identifying the characteristics of VvmiRNA SNP helps to recognition of the evolution of VvmiRNA and its overall roles in the process ofthe stone-hardening stage of grape berries. Amongst 161 types of VvmiRNAs/VvmiRNAs*, 71 types of VvmiRNA SNPs and corresponding Edit types were identified, while the mature sequences of the remaining 90 types of VvmiRNA remained unchanged (Table 2 and Supplementary Table S5). Furthermore, several VvmiRNA families exhibited high SNPs amongst their members. For example, VvmiR166, VvmiR156, and VvmiR167 families exhibited the highest SNPs amongst their members. In contrast, VvmiR169 family possessed the lowest SNP amongst eight members (Table 2). This finding suggested that divergence of conservation in the sequences amongst various VvmiRNA families (Table 2 and Fig. 3), similar to the previous report [23].

**Table 2:** List of VvmiRNAs Normal and SNP

miRNA_Name	SNP_Count	Normal_Count	SNP Rate	miRNA_Name	SNP_Count	Normal_Count	SNP Rate
VvmiR156a	10,847	65	99.40%	VvmiR169g	27	7	79.41%
VvmiR156b	150	11,417	1.30%	VvmiR169h	292	49	85.63%
VvmiR156c	145	10,753	1.33%	***VvmiR169i***	292	0	100.00%
VvmiR156d	157	11,570	1.34%	VvmiR169l	66	292	18.44%
VvmiR156e	9056	25	99.72%	VvmiR169r	269	57	82.52%
VvmiR156f	359	29,990	1.18%	VvmiR169u	291	17	94.48%
VvmiR156g	359	29,952	1.18%	VvmiR171e	116	1	99.15%
VvmiR156h	32	1	96.97%	VvmiR172d	14	2747	0.51%
VvmiR156i	359	29,952	1.18%	VvmiR2950	8	1773	0.45%
VvmiR159c	27	1335	1.98%	VvmiR2950	18	3029	0.59%
VvmiR160a	20	7	74.07%	VvmiR319e	63	100	38.65%
VvmiR160b	20	7	74.07%	VvmiR3623	18	4405	0.41%
VvmiR160c	7	17	29.17%	VvmiR3623	201	14,830	1.34%
VvmiR160d	7	21	25.00%	VvmiR3624	313	2652	10.56%
VvmiR160e	7	21	25.00%	VvmiR3629a	36	27	57.14%
VvmiR162	19	4100	0.46%	VvmiR3629b	34	27	55.74%
VvmiR164a	59	3790	1.53%	VvmiR3629c	36	27	57.14%
***VvmiR164b***	74	0	100.00%	VvmiR3631b	103	28	78.63%
VvmiR164c	65	3809	1.68%	VvmiR3633a	26	6164	0.42%
VvmiR164d	59	3791	1.53%	VvmiR3635	5	3021	0.17%
VvmiR166a	270	17,456	1.52%	VvmiR3636	195	12,946	1.48%
VvmiR166b	1683	101,565	1.63%	VvmiR3639	9	493	1.79%
VvmiR166c	6036	287,126	2.06%	VvmiR396a	708	753	48.46%
VvmiR166d	6040	287,317	2.06%	VvmiR396b	1163	1008	53.57%
VvmiR166e	6036	287,126	2.06%	VvmiR396c	614	257	70.49%
VvmiR166f	6055	287,902	2.06%	VvmiR396d	614	262	70.09%
VvmiR166g	6056	288,001	2.06%	VvmiR399a	5	10	33.33%
VvmiR166h	6324	301,916	2.05%	VvmiR399b	10	5	66.67%
VvmiR167a	19	341	5.28%	VvmiR399c	10	5	66.67%
VvmiR167b	120	9550	1.24%	VvmiR399h	5	10	33.33%
VvmiR167c	75	6468	1.15%	VvmiR479	465	34,737	1.32%
VvmiR167d	56	6285	0.88%	VvmiR535a	17	3427	0.49%
VvmiR167e	120	9501	1.25%	VvmiR535b	17	3427	0.49%
VvmiR168	1070	55,816	1.88%	VvmiR535c	17	3427	0.49%
VvmiR169b	292	50	85.38%	***VvmiR828b***	7	0	100.00%
VvmiR169f	27	7	79.41%				

Words with bold and italic denote the new generated VvmiRNAs

Interestingly, we observed that diverse VvmiRNA families had diverse SNP variations in their Edit types and numbers (Fig. 3), supported by the Amur grape report [23]. To depict this phenomenon clearly, all VvmiRNA families with SNPs were further classified into several groups (Fig. 3). In group I, most members of each VvmiRNA family had an SNP, and each member with an SNP possessed multiple Edit types of SNP. For example, the VvmiR166 family (VvmiR166s) had 8 members and 282 Edit types of SNV (8, 282), followed by VvmiR156s (9, 118), VvmiR167s (5, 40), VvmiR164s (4, 17), and VvmiR535s (3, 9), respectively. From these VvmiRNA families, diverse members with sequence variations obviously exhibited divergent Edit types of SNP. Although the diverse members of one miRNA family with various precursors possessed same mature sequences (e.g., VvmiR166b/c/d/e/f/g/h, VvmiR156b/c/d, VvmiR156f/g/i, VvmiR167b/c/d/e, VvmiR164a/c/d, and VvmiR535a/b/c), they had various Edit types. For instance, VvmiR166b and VvmiR166c/d/e/f/g/h had the same mature sequences, but they possessed 35 and 43 Edit types (35 and 43, respectively), resembling VvmiR156b, VvmiR156c and VvmiR156d (10, 10, and 11, respectively); VvmiR167b, VvmiR167c, VvmiR167d and VvmiR167e (12, 9, 5, and 12, respectively); and VvmiR164a, VvmiR164b, VvmiR164c, and VvmiR164d (5, 6, and 5, respectively) (Fig. 3). These findings suggested the diversification of the assorted VvmiRNA families in the evolution of the sequences. In group II, other VvmiRNA families only had one member, but it possessed multiple Edit types, such as VvmiR168 (34), VvmiR479 (31), VvmiR3636 (25), VvmiR3623*(19), VvmiR3624 (4), VvmiR3633a (4), VvmiR162 (3), VvmiR3623 (3), and VvmiR2950* (3). This finding implied that VvmiRNA families with single member exhibited drastic divergence and may thus be active factors during VvmiRNA sequence evolution. In group III, some VvmiRNA families such as VvmiR169b/c/g/h/i/l/r/u, VvmiR160a/b/c/d, VvmiR399a/b/c/d, and VvmiR3629 were also revealed only one Edit type even though they had multiple members with SNPs, indicating that they may possess relatively high conservation during VvmiRNA sequence evolution. In the final group, the remaining VvmiRNA families had fewer members and Edit types (Fig. 3). All these results confirmed the diversification of VvmiRNA families in the evolution of their mature sequences.

The total read number of VvmiRNAs with SNP was further revealed to reach 77,141,827, and diverse VvmiRNA families and their various members had conspicuous divergence in the number of sequences with SNP. Amongst them, VvmiR166 and VvmiR156 families had considerably more reads with SNP than the other families (Table 2). Generally, the number of SNPs in VvmiRNA families was less than that of the normal sequences. However, the SNP sequences of 21 VvmiRNAs had more read numbers than miRNAs themselves, including VvmiR156a, VvmiR156e, VvmiR156h, VvmiR160a, VvmiR160b, VvmiR169b, VvmiR169f, VvmiR169g, VvmiR169h, VvmiR169r, VvmiR169u, VvmiR171e, VvmiR3629a, VvmiR3629b, VvmiR3629c, VvmiR3631b*, VvmiR396b, VvmiR396c, VvmiR396d, VvmiR399b, and VvmiR399c, suggesting that these VvmiRNAs had stronger evolution than the others. Interestingly, compared with homologous VvmiRNAs from the grape cv. ‘Pinot Noir’ in miRBase 21.0 (http://www.mirbase.org/summary.shtml?org=vvi), some VvmiRNAs could not be identified in this work. However, their SNP sequences such as VvmiR164b, VvmiR169i, and VvmiR828b were identified (Supplementary Table S5; bold and italic words). Thus, SNPs may explain the generation of new members of VvmiRNA family in miRNA evolution.

### Functional annotation of specific-VvmiRNA targets during grape stone-hardening stage

**Fig. 4:**
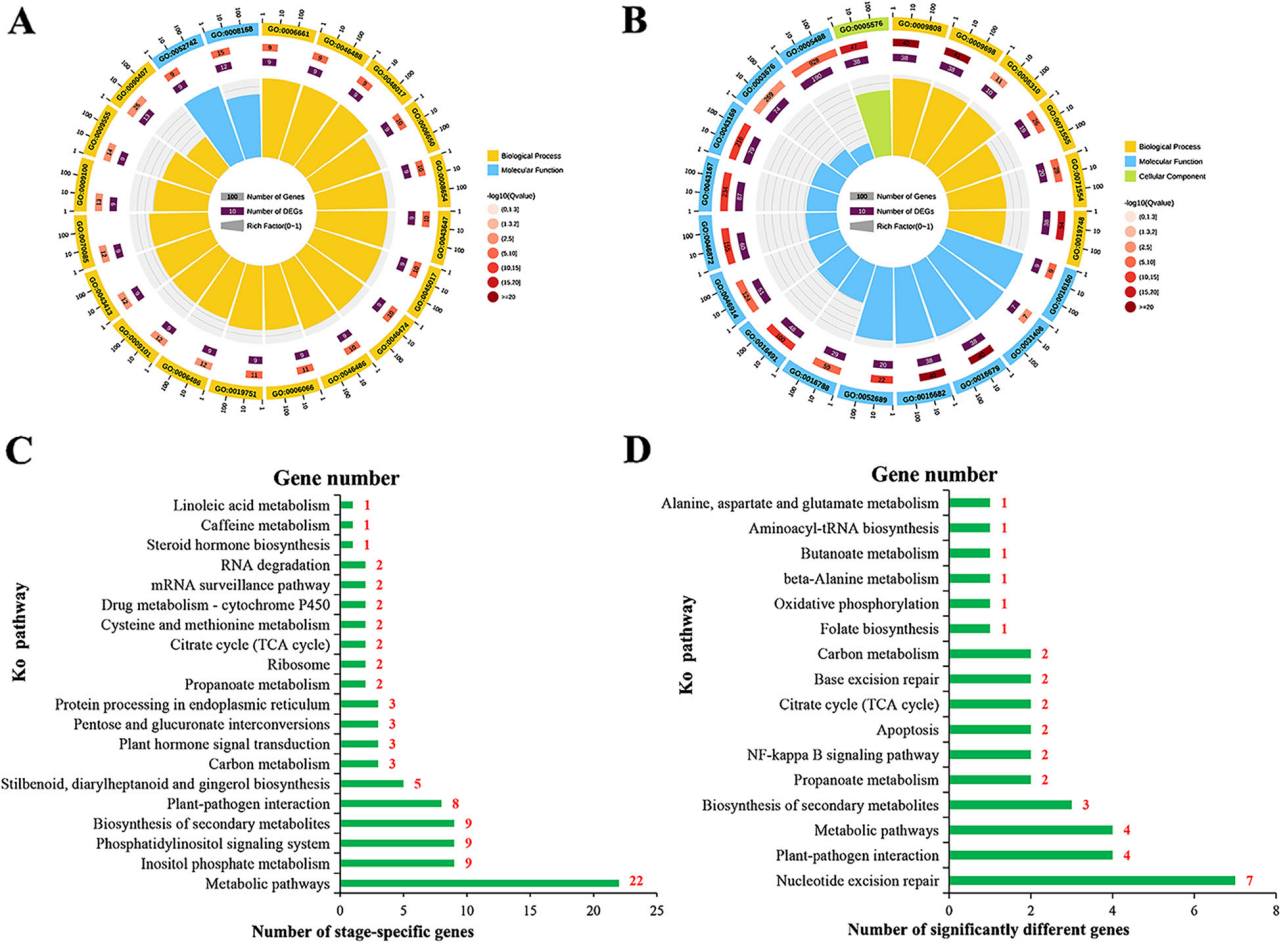
Number of target genes for VvmiRNAs involved in diverse Gene Ontology (GO) and KEGG pathway. Notes: Number of stage- specific VvmiRNAs (**a**) and VvmiRNAs with significant difference (**b**) target genes enriched in biological process, molecular function and cellular component. Top KEGG pathways highly enriched in stage- specific VvmiRNAs (**c**) and VvmiRNAs with significant difference (**d**) target genes

To further recognize the roles of VvmiRNA during grape stone-hardening stage, PsRNATarget software (http://plantgrn.noble.org/psRNATarget/result?sessionid=1503987414486479) was utilized to predict the potential miRNA target genes on the basis of our previous RNA-seq data (GEO Accession: GSE77218) by using mature miRNA sequences as queries. A total of 2124 targets for known VvmiRNAs and 885 targets for novel VvmiRNAs were predicted in this work, amongst which 1639 and 1635 target genes may be targeted by 24 stone-hardening-specific VvmiRNAs and VvmiRNAs with significant expressional difference, respectively. GO and KEGG analyses were performed with these predicted target gene sequences to improve our understanding of their functions in grape stone-hardening stage. Totally, 13 VvmiRNAs might be involved in the regulation of embryo development, 11 in lignin and cellulose biosynthesis, and 28 in the modulation of hormone signaling, sugar, and proline metabolism (Supplementary Table S6). From the Fig. 4a, the top 20 of GO enrichment of the stone-hardening-specific VvmiRNA targets included 18 GO enrichment of biological process and 2 of molecular function, whereas those of VvmiRNAs with significant difference had 6 GO enrichment of biological process, 13 of molecular function, and 1 of cell component (Fig. 4b). Further comparison of the biological processes between Fig. 4a and Fig.4b, the target genes for stone-hardening-specific VvmiRNAs primarily focused on the lipid, phosphatelipid biosynthesis/metabolic process (GO: 0008610; GO: 0008654; GO: 0006644), phosphatidylinositol biosynthesis/metabolic process (GO: 0006661; GO: 0046488), and glycerophospholipid/glycerolipid biosynthetic/metabolic process (GO: 0046474; GO: 0006650; GO: 0046486). These GO pathways were closely related to seed development, supporting the modulation of the stone-hardening-specific VvmiRNAs on grape seed development. Whereas, those of VvmiRNAs with significant difference largely participated in cellular metabolic process (GO: 004237), ligin metabolic process (GO: 0009808), secondary/aromatic metabolic process (GO: 0019748; GO: 0019439), cell-wall organisation or biogenesis (GO:0071555; GO: 0071554), and peptidyl-proline modification (GO: 0018208), thereby providing evidence of supporting their potential regulatory roles in grape-berry development and quality formation.

Meanwhile, an overview of the KO pathways of VvmiRNA-mediated target genes was further generated by KEGG pathway analysis (http://www.genome.jp/kegg/). A total of 73 KO pathways were identified by 228 targets for VvmiRNAs (Supplementary Table S6), among which the stone-hardening-specific VvmiRNAs-mediated target genes largely participated in the KO pathways as showed in Fig. 4c. Amongst them, metoblism pathway (ko01100), inostol phosphate biosynthesis (ko00562), phosphatidylinositol signaling system (ko04070), biosynthesis of secondary metabolites (ko01110), stilbenoid, diarylheptanoid and gingerol biosynthesis (ko00945), carbon metabolism (ko01200), plant hormone signal transduction (ko04075), and pentose and glucuronate interconversions (ko00040) were the main pathways. These results supported this view of these specific VvmiRNAs involved in the modulation of seed and berry development during grape stone-hardening stage. By contrast, VvmiRNAs had significant difference-mediated target genes in 17 KO pathways (Fig. 4d), primarily enriched in nucleotide excision repair (ko03420), metabolic pathways (ko01100), biosynthesis of secondary metabolites (ko01110), NF-kappa B signaling pathway (ko04064), apoptosis (ko04210), citrate cycle (TCA cycle) (ko00020), carbon metabolism (ko01200), and so on. These findings indicated the potential role of these VvmiRNAs in the regulation of grape-berry development.

### Verification of target genes for novel VvmiRNAs related to berry development at the stone-hardening stage of grape berries

Based on the expression profiles of novel VvmiRNAs (Fig. 2c), together with their potential functional annotation, we found four novel VvmiRNAs closely related to the berry development of VvmiR8-5p, VvmiR31-3p, VvmiR38-5p, and VvmiR53-3p. Their corresponding target genes involved in embryo and seed stone development [VIT_204s0008g03060, DDB1- and CUL4-ASSOCIATED FACTOR HOMOLOG 1 (*VvDCAF1*)] [28], GA signaling [VIT_217s0000g10300, *GIBERELLIN INSENSITIVE* (*VvGAI1*)] [29], lignification [VIT_212s0028g03110, *CAFFEOYL-CoA O-METHYLTRANSFERASE* (*VvCCoAOMT*)] [30], and cell wall deacetylation [VIT_218s0041g02160 (*ESTERASELLIPASE*,*VvGDSL*)] [31], which were further selected to verify their roles at the stone-hardening stage of grape berries. The cleavage interactions of these four VvmiRNAs on their target genes above at the berries of grape stone-hardening stage were verified with our modified RNA ligase-mediated 5′-rapid amplifification of cDNA ends (RLM-RACE) and developed poly(A) polymerase-mediated 3′-rapid amplifification of cDNA ends (PPM-RACE) procedures [32, 33], which are high effeciency, low cost strategies for validating the true target genes for miRNAs by sequencing the 3′- / 5′- end cleavage products of their target genes.

**Fig. 5:**
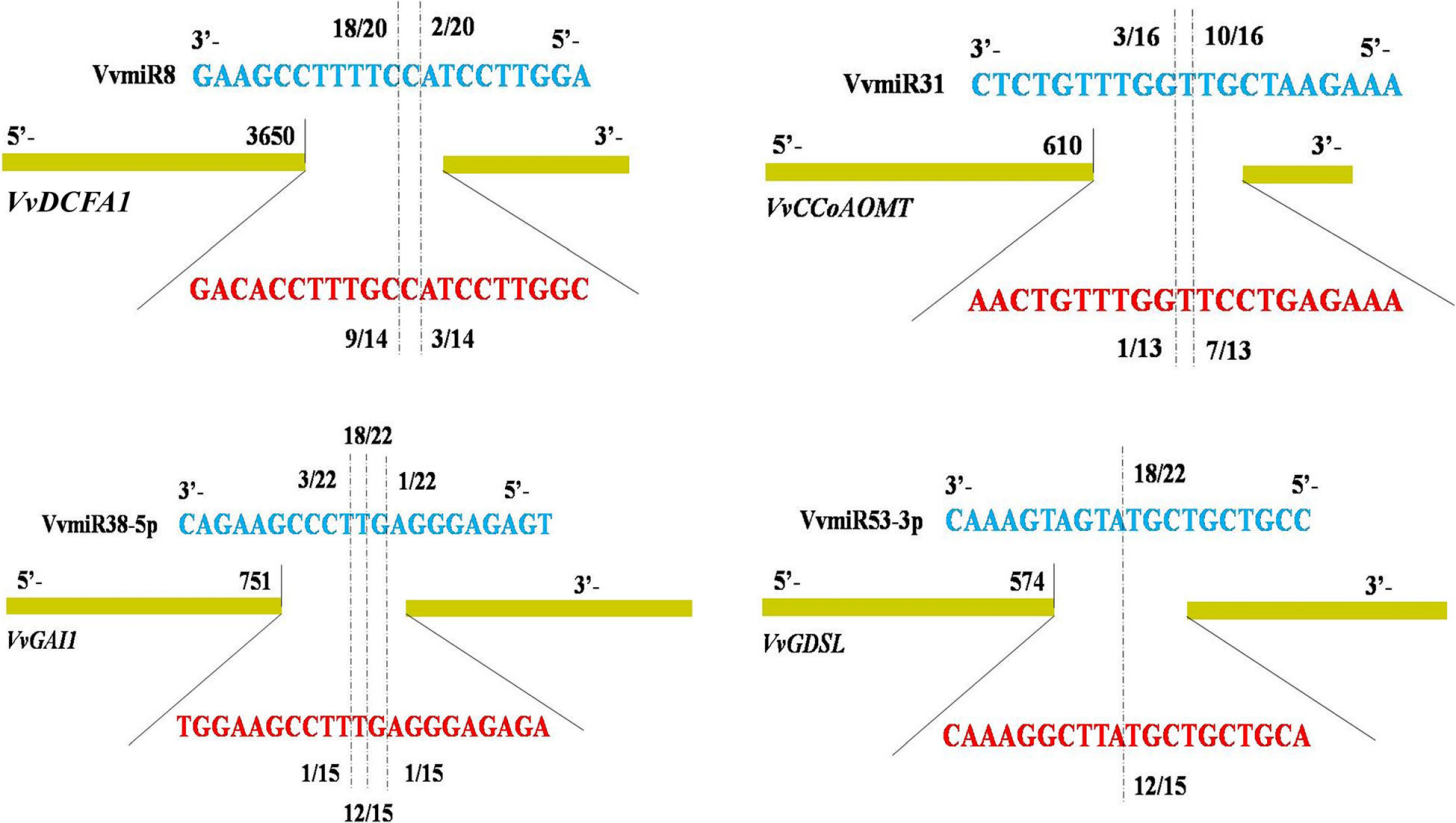
Validation of target genes cleaved by VvmiRNAs using RLM-RACE and PPM-RACE. The gray dotted lines indicate the cleavage sites of VvmiRNAs on target genes identified by 5′ and 3′-ends of mRNA fragment using PPM-RACE and RLM-RACE, respectively. The blue and red regions denote the sequences of VvmiRNAs and complementary sequences of VvmiRNAs on their target genes. Number above the blue regions denote the cleavage counts by RLM-RACE, while the number below the red regions indicate the cleavage counts by PPM-RACE. Each assay was repeated the three times

First, using RLM-RACE, the sequencing of the amplified 3′-end products confirmed that the VvmiRNAs’ cleavage on their target genes occurred at the 9th–11th sites, amongst which the 10th was their main cleavage site with the highest cleavage frequency (Fig. 5). This result indicated the specificity of cleavage sites of miRNAs, consistent with previous findings [32, 33]. Although VvmiR8 and VvmiR31 had two cleavage sites on their corresponding targets (*VvDCAF1* and *VvCCoAOMT*), the cleavage sites of the former were at the 9th and the 10th (the highest frequency 18/20), and those of the latter were at the 10th (the highest frequency 10/16) and the 11th. Similarly, VvmiR38-5p on *VvGAI1* possessed three cleavage sites at the 9th–11th, amongst which the 10th had the highest frequency 18/22. Meanwhile, VvmiR53-3p on *VvGDSL* possessed only one cleavage site at the 10th with the same high frequency 18/22. Next, our developed PPM-RACE was used to further confirm the target genes of VvmiR8-5p, VvmiR31-3p, VvmiR38-5p, and VvmiR53-3p and their cleavage sites. The sequencing of the amplified 5′-end products identified the same cleavage sites as those of the 3′-end sequencing in the RLM-RACE experiment, but all their 5′-end cleavage product accumulation levels and their cleavage frequency detected in PPM-RACE were lower than their corresponding 3′-end cleavage frequency examined in RLM-RACE (Fig. S1; Fig. 5). This phenomenon may be due to the fact that the 5′-end cleavage products were more easily degraded than the 3′-end cleavage products, similar to previous reports [32, 33]. The consistent results of the RLM-RACE and PPM-RACE experiments demonstrated that *VvDCAF1*, *VvCCoAOMT*, *VvGAI1*, and *VvGDSL* were the true target genes of VvmiR8-5p, VvmiR31-3p, VvmiR38-5p, and VvmiR53-3p, thereby verifying their cleavage-interaction mode in the stone-hardening stage of grape berries.

### Expression modes of VvmiRNAs and their target genes during GA-induced grape seedless-berry development

**Fig. 6:**
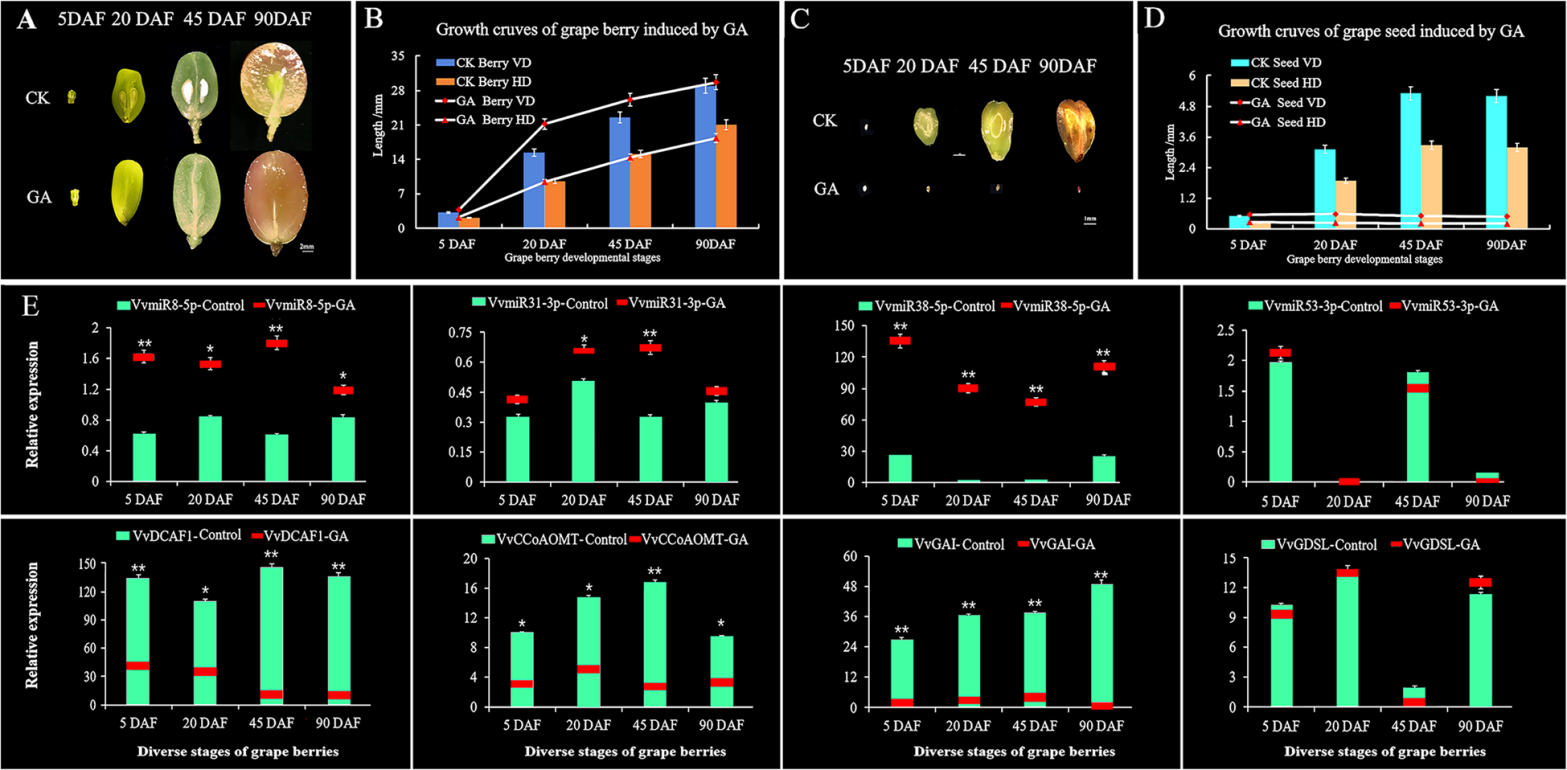
Characterization of grape berries at the seed coat-hardening stage and Gibberellin (GA_3_)-induced grape parthenocarpic berries. **a-b** Variation in morphology and growth curve of grape berries derived from GA_3_-treated and untreated control (CK) plants. **c-d** Variation in morphology and growth curve of seeds derived from GA_3_-treated and CK control plants. Vertical diameter, VD; horizontal diameter, HD. **e** Expression levels of VvmiRNAs responsive to GA_3_-treatment during grape berry development [5 days after flowering (5DAF), 20DAF, 45DAF, 90DAF]. The mean and SD values were obtained from three biological samples. ANOVA test was used to identify significant differences, Asterisks indicated statistically significant differences at (*P < 0.05; **P < 0.01) as determined by Student’s *t*-test

To gain insight into seed development during grape stone-hardening stage and GA-induced parthenocarpy process, we compared berries phenotype and seed morphology of GA-treated and untreated control (CK) ‘Wink’ grape cultivar at 5, 20, 45, and 90 DAF were carried out (Figs. 6a–d). Amongst them, the berries at 45 DAF in untreated control group had full seeds and hardened seed coats (Fig. 1a). GA-treated grape berries showed more distinct increased in vertical diameter of berries grains at 20 and 45 DAF as compared with untreated CK groups, but no variation were observed in horizontal diameter (Fig. 6b). Additionally, GA-treated grapes showed 99.6% seedless rate (Fig. 6d), compared with untreated CK groups, GA-treated grape seeds were drastically inhibited growth, and there were almost no growth from fruit setting to maturation. These results confirmed the profound effects of GA-induced ‘Wink’ grape parthenocarpy, suggesting that complicated regulatory networks might exist during GA-mediated grape berries and seed development.

To determine the long-term roles of VvmiRNAs and their target genes validated above during GA-induced grape seedless-berry development, the expression levels of *VvMIR8-5p*, *VvMIR31-3p*, *VvMIR38-5p*, and *VvMIR53-3p* and their corresponding target genes *VvDCAF1*, *VvGAI1*, *VvCCoAOMT*, and *VvGDSL* were examined in berries at 5, 20, 45, and 90 DAF, respectively. Results (Fig. 6e) showed that except for *VvMIR53-3p* and its target gene, the remaining three *VvMIRNAs* and their target genes exhibited significant expression differences in response to GA treatments relative to untreated control plants, whereas *VvMIR53-3p* and its target gene *VvGDSL* hardly differed in response to GA treatment than the control. Notably, the former three miRNAs *VvmiR8-5p*, *VvmiR31-3p*, and *VvmiR38-5p* expression were strongly up-regulated by GA treatment. Specifically, *VvmiR8-5p* and *VvmiR31-3p* displayed the highest expression level at the grape stone-hardening stage (45 DAF) in response to GA treatment relative to other stages (Fig. 6e), indicating that these two miRNAs may play significant roles by responding to GA during the stone-hardening stage of grape berries. By contrast, the expression of their target genes *VvDCAF1*, *VvCCoAOMT*, and *VvGAI1* exhibited strong down-regulation in response to GA treatment (Fig. 6e). VvmiRNAs and their target genes above also displayed the opposite expression trends during GA-induced grape seedless-berry development, confirming that these VvmiRNAs negatively modulated their target genes’ expression during this process.

Interestingly, GA treatments significantly down-regulated the expression of *VvGAI1*, which is a key DELLA protein negative interaction factor in the GA signal pathway (Fig. 6e). Similarly, during grape stone-hardening stage GA treatment also significantly down-regulated the key genes *VvCCoAOMT* and *VvDCFA1*, which were involved in lignin biosynthesis and embryo development, (Fig. 6e). These results indicated that GA may repress grape stone hardening and embryo development by inducing the expression of *VvmiR31-3p* and *VvmiR8-5p* to negatively regulate the expression levels of *VvCCoAOMT* and *VvDCAF1,* as a key molecular mechanism involved in the modulation of GA-induced grape seedless-berry development. However, GA exhibited no effect on the expression levels of *VvmiR53-3p* and *VvGDSL* target genes compared with the untreated control, due to the fact that *VvGDSL* was a gene related to cell-wall development. Meanwhile, we revealed that *VvGDSL* exhibited the highest expression level at 20 DAF (berry-expansion stage), implying that it may participate in the modulation of the cell-wall expansion development of young berries.

### Dynamic accumulation of cleavage products of the four VvmiRNAs’ target genes during GA-induced grape-berry development

**Fig. 7:**
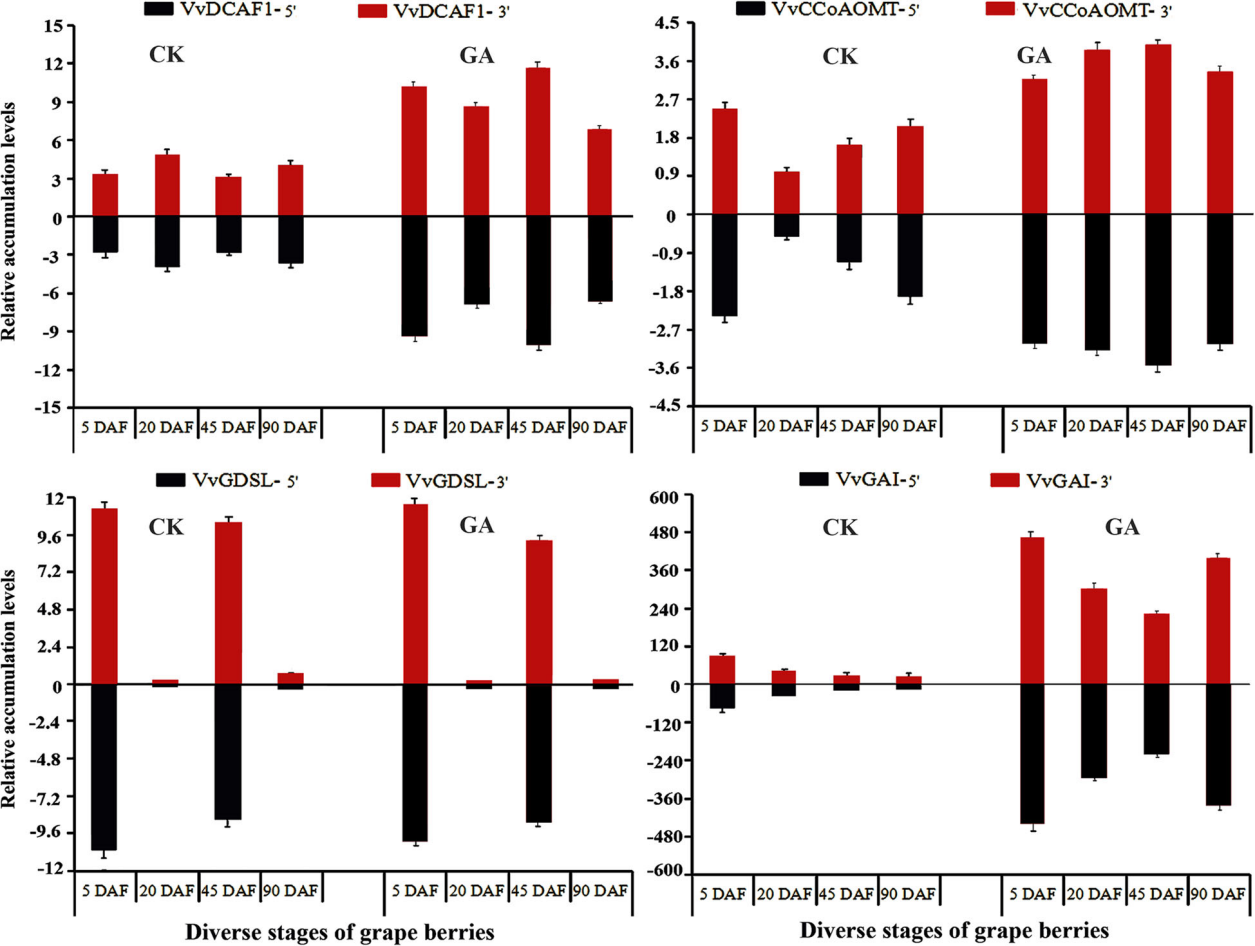
Accumulation levels of 3′−/5′-end cleavage products of VvmiRNAs on their target genes in GA-treated and untreated control (CK) plants at different stages of grape berry development by RLM-RACE and PPM-RACE. 3’DCAF1 denotes 3′-end cleavage products of target genes by VvmiRNAs, whereas 5’DCAF1 indicates 5′-end cleavage products of target genes by VvmiRNAs. Each experiment was repeated three times. ANOVA test was used to identify significant differences, Asterisks indicated statistically significant differences at (*P < 0.05; **P < 0.01) as determined by Student’s *t*-test

Monitoring the accumulation patterns of these four VvmiRNAs’ cleavage products and target genes during GA-induced grape seedless-berry development could contribute to determining the variation of their cleavage roles. Here, the 3′-and 5′-end cleavage product accumulation levels were examined by RLM-RACE and PPM-RACE, respectively. Results showed similar dynamic accumulation modes of both ends-cleavage products during different stages of grape-berry development (Fig. 7), confirming the dynamic variation in cleavage roles of these VvmiRNAs on their target genes during this process. Moreover, the accumulation modes of cleavage products resembled the expression modes of the corresponding VvmiRNAs, indicating that miRNAs may be the main factors affecting their interactions. Interestingly, we found that GA evidently promoted the cleavage roles of VvmiR8-5p, VvmiR31-3p, and VvmiR38-5p on their corresponding targets by obviously up-regulating accumulation levels of the corresponding target cleavage products, while VvmiR53-3p/*VvGDSL* pair with nearly no change under GA treatment. In particular, the cleavage products of VvmiR31 and VvmiR8 on their corresponding targets *VvCCoAOMT* and *VvDCFA1* were boosted at the highest level at 45 DAF (grape stone-hardening stage), which may derive from the correlation of these two targets’ potential functions with embryo and stone development [28, 30]. Unlike this, those of VvmiR38-5p on *VvGAI1* were enhanced drastically by GA at all stages of GA-induced seedless berries used in this work. These results suggested that GA might involved in manipulating grape seedless-berry development primarily by promoting the cleavage role of VvmiR38-5p on *VvGAI1* at all stages here. It is inferred that GA might repress grape stone hardening and embryo development through negatively regulating the expression levels of the lignin biosynthesis gene *VvCCoAOMT* and embryo development related gene *VvDCAF1* by inducing the expression of VvmiR31-3p and VvmiR8-5p, as a potential key molecular mechanism involved in the modulation of GA-induced grape seedless-berry development.

## Discussion

Grape stone-hardening stage is the critical stage for seed embryo development [8]. When grape inflorescences are treated with GA at 10 days before anthesis, seed embryo development and seed coat formation of grape berries are inhibited, leading to seedless-berry formation [12]. Thus, GA is a key phytohormone regulator for seed embryo development and stone-hardening in grape. Previous studies have revealed that some VvmiRNAs are differentially expressed during grape-berry development in response to GA application [8, 12, 21, 34]. However, research on stone-hardening stage-specific VvmiRNAs and their regulatory networks during grape-berry development remains imperative. Accordingly, we identified and characterized stone-hardening stage-specific miRNAs and their mRNA target genes in response to GA in the current work to gain in-depth insights into the molecular basis of seedless-berry development for the molecular breeding of novel seedless-grape varieties.

### VvmiRNA-mediated TFs and methylation−/acylation-related genes in grape during seed-hardening stage

**Fig. 8:**
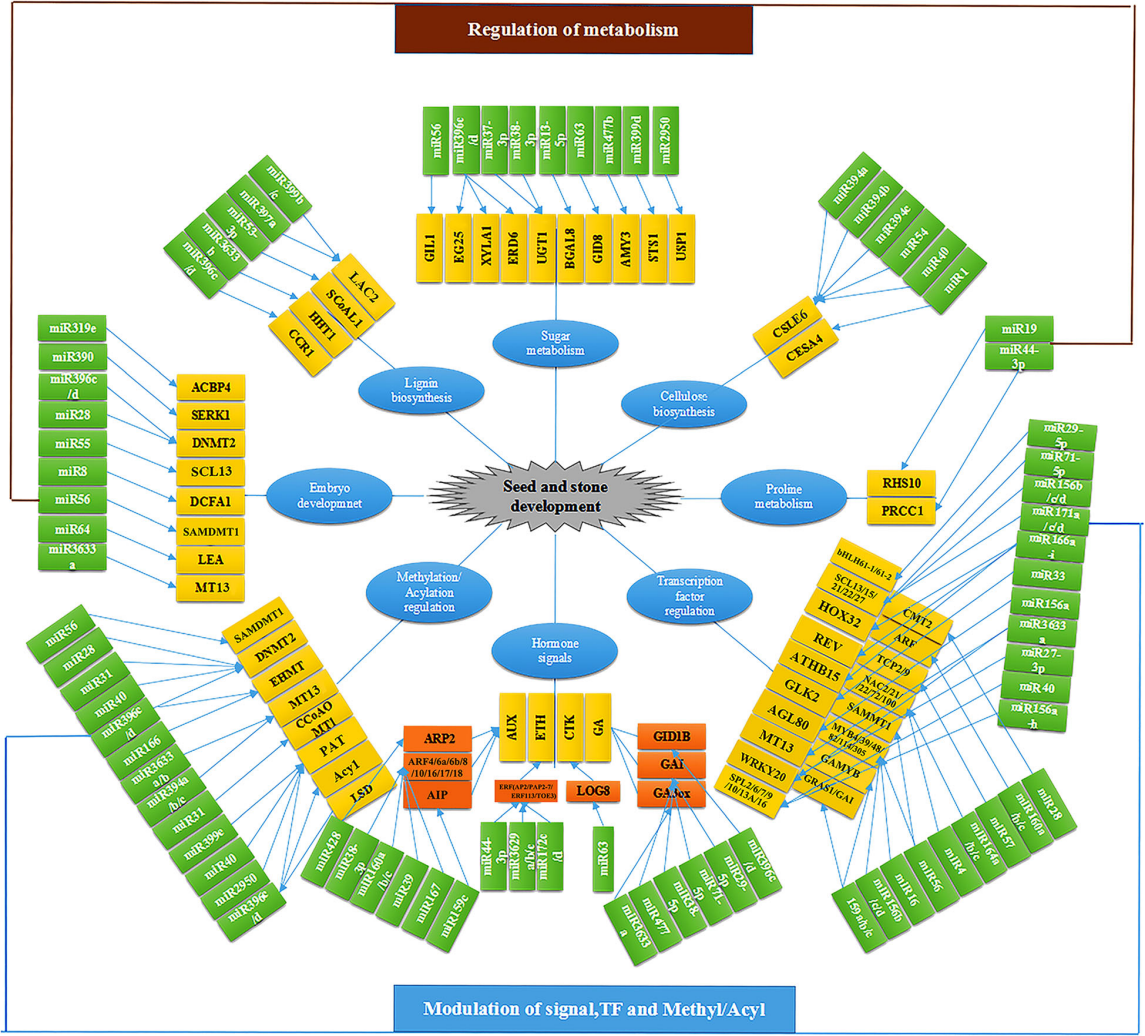
A mode chart of miRNA-mediated regulatory network related to grape berry seed and stone development. Green rectangle represents diverse VvmiRNAs which might regulate seed and seed coat development, whereas yellow rectangle indicates their corresponding target genes. The blue ellipse donates the metabolism pathways and specific tissues of VvmiRNAs and their target gene mediated in grape seed and seed coat development

In this work, the potential target genes of stone-hardening stage-specific VvmiRNAs were predicted. Out of them, some target genes were the members of TF families, such as SPL (2/6/7/9/10/13A/16), SCL (13/15/21/22/27), GRAS (GRAS1/GAI), HB-Zip (HOX32/REV/ATHB15), MADS-box (AGL17/24/62/80/SVP/JONTLESS-like), ERF (AP2/PAP2–7/ERF113/TOE3), MYB (GAMYB/MYB4/39/48/82/114/305), ARF (4/6a/6b/8/10/16/17/18), NAC (2/21/22/72/100), bHLH (61–1/61–2), GATA (5/24), TCP (2/9), GLK 2, and WRKY20 (Fig. 8), indicating VvmiRNA could regulate berry development including seeds through regulating expression of these TFs at stone hardening state. Similar cases were reported by previous studies [35]. For example, ZmMYB138 and ZmMYB115 for ZmmiR159k regulated the endosperm development of corn seeds by affecting the transcriptional activities of *Du1/Wx* and *Ae1/Bt2* genes [36]. MYB89 was expressed predominantly in developing seeds during maturation, which inhibited seed oil accumulation by directly repressing WRI1 [37]. AP2, WRKY TF SHB1, WRKY10 and LEC1 controlled the development of seeds in rice and *Aribidopsis* [38–40]. Additionally, *VvHD-Zips* and *VvMADS39* were closely related to embryo abortion in grape berries [41, 42]. BpMADS12 promotes lignin accumulation in *B. platyphylla* [43, 44]. All these findings supported the view that miRNA-mediated TFs might participate in the regulatory network of seed development and seed coat-hardening formation (lignin) in grape.

In addition, another VvmiRNAs at grape stone-hardening stage were predicated to target some methylation/acylation-related genes. As shown in Fig. 8, *VvASMDMT1*, *VvDNMT2*, *VvEHMT*, *VvMT13*, *VvCCoAOMT1*, *VvPAT*, *VvAcyl*, and *VvLSD* were predicated as the potential target genes for VvmiR28, VvmiR31, VvmiR40, VvmiR56, VvmiR166, VvmiR2950, VvmiR394a/b/c, VvmiR3633a/b, VvmiR396c/d, and VvmiR399e, suggesting these VvmiRNAs above might regulate their potential target genes related to methylation/acylation, whereby involving in modulatiion of grape berry development process. Similarly, previous studies reported that DNA methylation plays important roles in modulation of fruit development [45, 46]. Specific tissues have their own methylome patterns, the epigenome is not static, and extensive reprogramming occurs during tomato fruit development [47]. In apple and pear, DNA methylation of MYB transcription factor can regulate fruit pigment accumulation and fruit color [48, 49]. Therefore, the current work provides the several potential genes for gaining in-depth insights into fruit development.

### VvmiRNA-targeted genes involved in hormone signal pathways in grape berry at the stone-hardening stage

GA and AUX play essential roles in grape berry and seed development [8, 12, 50]. Here, amongst our identified VvmiRNAs in grape berries at the stone-hardening stage, several VvmiRNA-mediated target genes were found to be involved in hormone metabolism and signal transduction according to functional annotations (Fig. 8). From the Fig. 8, *VvGA3ox* is the potential target of VvmiR3633, which is involved in the GA biosynthesis pathway, whereas *VvGID* (the receptor of GA signal), as the predicted target for VvmiR396, plays a key role in the GA signal-transduction pathway (Fig. 8). Similarly, *VvDELLAs* for VvmiR477 and *VvGAMYB* for VvmiR159 and VvmiR319 are the core elements of the GA signal-transduction pathway. Furthermore, GA treatment induced the expression levels of miR159s, leading to the reduction in the expression of *GAMYB* level, which delayed flowering, perturbed anther development, and promoted parthenocarpy and subsequently seedless-fruit formation in *Arabidopsis*, tomato, and grape plants [18, 51]. Likewise, GA treatment down-regulated the expression levels of miR319 and miR166 during the modulation of *Arabidopsis* and tomato plant development [51, 52].

Our previous study also revealed that VvmiR160s may mediate auxin signal transduction to regulate grape berry and seed development at the stone-hardening stage by mediating target genes *ARFs* [13]. Similarly, it has been reported that miR160 and miR167 have important regulatory roles in female and male reproduction and the parthenocarpy of *Arabidopsis* plants [51, 52]. Moreover, *VvAP2* and *VvERF*, ethylene signaling-related genes were identified as target genes for VvmiR172 and VvmiR3629 (Fig. 8). These findings suggested that VvmiRNA-mediated phytohormone signaling was an essential step during early seed development in grape. This result was in agreement with that of Curbara et al. [53], who reported that ARFs and TIR1, as auxin receptors, are negatively regulated by miR160, miR167, miR390, and miR393; and the ABA-insensitive gene *ABI3* is repressed by miR516 during the early seed development of barley [53]. All these findings implied that VvmiRNAs may negatively regulate several hormone-signal-related genes during the modulation of grape berry and seed development at the stone-hardening stage.

### VvmiRNA-mediated regulatory networks in berry and seed development at the grape stone-hardening stage

The characterization of the potential target genes of VvmiRNAs at the grape stone-hardening stage is the key step for elucidating the miRNA-mediated regulatory networks associated with grape berry and seed development. Recently, some miRNAs and their target genes have been reported during fruit development in tomato [54], eggplant [55], pear [56], grape [23, 57], citrus [58, 59], banana [25], melon [60], apple [61], and strawberry [62, 63]. In this work, we identified many key target genes related to berry development for VvmiRNAs involved in sugar, acid, pigmentation and hormone metabolism, lignin synthesis, and methylation/acylation process in grape berries and seeds (Fig. 8). On the basis of the identification and characterization of VvmiRNAs and their target genes in grape berries at the stone-hardening stage, a putative schematic mode of VvmiRNA-mediated berry and seed development was proposed. As shown in Fig. 8: i) some VvmiRNAs might be involved in the development of grape seeds and embryos by mediating their target genes, including VvmiR8, VvmiR27-3p, VvmiR28, VvmiR55, VvmiR56, VvmiR64, VvmiR156a, VvmiR166a-h, VvmiR171a/c/d, VvmiR319e, VvmiR3633a, VvmiR390, and VvmiR396c/d (corresponding yellow regions, same as below); ii) 19 other VvmiRNAs might participate in the modulation of lignin biosynthesis of grape stone by mediating their target genes in lignin biosynthesis, including VvmiR53-5p, VvmiR156b, VvmiR164a/c, VvmiR166a-h, VvmiR3633b, VvmiR396c/d, VvmiR397a, and VvmiR399b/c; iii) two VvmiRNAs of VvmiR19 and VvmiR44-3p might regulate seed and stone development by targeting the genes related to proline metabolism; iv) 10 VvmiRNAs might participate in the modulation of sugar metabolism, including VvmiR13-5p, VvmiR27-5p, VvmiR37-3p, VvmiR56, VvmiR63, VvmiR2950, VvmiR396c/d, and VvmiR477b; and v) six other VvmiRNAs might manipulate cellulose biosynthesis by mediating *VvCSLE6* and *VvCESA4*, including VvmiR394a/b/c, VvmiR54, VvmiR40, and VvmiR1. Our putative schematic mode was supported by previous studies [11], which reported that miR156, miR164, miR1132, miR5077, and miR396b regulate sugar and acid metabolism in *Lyciumbarbarum* and pear fruits, whereas miR397a mediated-*LACs* manipulate the lignin synthesis of pear fruits, poplar, and *Arabidopsis* [7, 64–66]. Furthermore, miR156, miR166, miR167, miR168, miR393, miR172, and miR396 are preferentially/highly expressed during embryo development, whereas miR164 is primarily expressed in seeds [67]. These insights into the miRNA-mediated seed and stone regulatory networks in grapes could contribute to the understanding of the molecular regulatory mechanism during grape-berry development in the stone-hardening stage at the global transcriptome-wide level.

### Regulatory modes of VvmiRNA-mediated seedless berries during GA-induced grape parthnocarpy

GA is one of the key hormones inducing parthenocarpy to produce seedless fruit, and expression changes of genes in the GA signaling pathway could induce parthenocarpy fruit set and fruit development. Meanwhile, DELLA protein is the key repressor of GA signal transduction, and the reduction of DELLA protein activity could lead to released GA signal and the appearance of corresponding phenotypes, such as parthenocarpy [68]. Nowadays, exogenous GA is extensively used to induce grape parthnocarpy, and recent studies have shown that GA signaling induces the expression level of miR159 and miR160 to regulate grape parthnocarpy, producing seedless berries [12, 13]. Here, we also found GA might repress the expression of the lignin biosynthesis enzyme gene *VvCCoAOMT* and the embryo developmental gene *VvDCAF1* to produce grape seedless berries by up-regulating VvmiR31-3p and VvmiR8-5p during GA-induced grape parthenocarpy process. With research development, more miRNAs are identified to be involved in the modulation of GA-mediated parthenocarpy, which would contribute to gain more insight into the regulatory mechanism of miRNA- mediated grape seedless berries induced by exogenous GA.

## Conclusion

In the present study, a total of 161 conserved and 85 species-specific miRNAs/miRNAs* (precursor) were identified in grape berries at stone-hardening stage using Solexa sequencing. Out of them, 30 were stone hardening stage-specific VvmiRNAs. And, high SNP variations in VvmiRNA sequences resulted into the generation of new VvmiRNA family memberslike VvmiR168, VvmiR479, VvmiR3636 families and so on. Among stone-hardening stage VvmiRNAs,13 VvmiRNAs might be involved in the regulation of embryo development, 11 in lignin and cellulose biosynthesis, and 28 in the modulation of hormone signaling, sugar, and proline metabolism. The target genes for 4 novel VvmiRNAs were validated using RLM-RACE and PPM-RACE methods, and their cleavage primarily occurred at the 9th–11th sites from the 5′ ends of miRNAs at their binding regions. Furhtermore, among the 4 novel VvmiRNAs above, GA could up-regulate the expressions of VvmiR31-3p and VvmiR8-5p, thereby inhibiting those of their target genes *VvCCoAOMT* (the lignin biosynthesis enzyme gene) and *VvDCAF1* (the embryo developmental gene) to produce grape seedless berries, as a potential key molecular mechanism involved in GA-induced grape seedless berry development. Finally, a schematic model of miRNA-mediated grape seed and stone-hardening development was proposed. Our results can serve as valuable references for the molecular breeding of seedless grape berries.

## Methods

### Plant materials and GA treatment

Five-year-old ‘Wink’ grape cultivar used as the experimental material was grown in experimental field at Jiangpu Farm of Nanjing Agricultural University, Nanjing City, Jiangsu Province, China. First, the grape berries at 45DAF stage were used for high throughput sequencing. Second, based on our preliminary trails, the total 18 inflorescence clusters with similar growth status from 6 grape plants were selected as materials, of which the 9 clusters were dipped into 50 mg/L GA_3_ for 30s at 10 days before flowering to induce grape seedless berries. The other remaining 9 clusters were treated with water and used as control set. In the early morning (9 to 10 AM), 3–4 grains from the middle of each cluster of GA_3_-treated and water-treated control plants at different time points [5 days after flowering (5DAF), 20DAF, 45DAF and 90DAF] were collected and immediately frozen in liquid nitrogen and stored at − 80 °C until use. Each type of samples consisted of three biological replicates.

### Small RNA (sRNA) library construction and high throughput sequencing analysis

Total RNA was extracted from 200 μg each sample from four grape berries at 5, 20, 45, 90DAF stages respectively using our modified CTAB method for small RNA high-throughput sequencing and qRT-PCR library construction [69]. Libraries were prepared with 1 μg total RNA for each sample. Total RNA samples were purified by electrophoretic separation on a 15% urea denaturing polyacrylamide gel electrophoresis (PAGE) gel and small RNA regions corresponding to the 18–30 nt bands in the marker lane (14–30 ssRNA Ladder Marker, TAKARA) were excised and recovered. Then the 18–30 nt small RNAs were ligated to adenylated 3′ adapters annealed to unique molecular identifiers (UMI), followed by the ligation of 5′ adapters. The adapter-ligated small RNAs were subsequently transcribed into cDNA by SuperScript II Reverse Transcriptase (Invitrogen, USA) and then several rounds of PCR amplification with PCR Primer Cocktail and PCR Mix were performed to enrich the cDNA fragments. The PCR product for cDNA at 45DAF were selected by agarose gel electrophoresis with target fragments 110 ~ 130 bp, and then purified by QIAquick Gel Extraction Kit (QIAGEN, Valencia, CA). The libraries were quality and quantitated in two methods: check the distribution of the fragments size using the Agilent 2100 bioanalyzer, and quantify the library using real-time quantitative PCR (qPCR) (TaqMan Probe). The final ligation PCR products were sequenced using the BGISEQ-500 platform (BGI-Shenzhen, China). On the other hand, four cDNA libraries at 5, 20,45,90 DAF for miRNAs were used as templates to detect miRNA expression levels (see qRT-PCR method section).

After obtaining the sequencing results, further trimming and filtering the adaptor and low-quality tag sequences, and the high-quality sRNA clean reads were mapped into the Rfam (https://rfam.xfam.org) to filter the rRNA, tRNA, snRNA, and snoRNA. The filtered reads were then compared against known plant miRNA database in the miRBase 21.0 (http://www.mirbase.org/) with BLASTn. After searching against Rfam database and miRBase, the remaining reads were further mapped to the grape reference genome (http://genomes.cribi.unipd.it/DATA/V2/).

### Bioinformatics analysis and identification of VvmiRNA and VvmiRNA SNPs

The clean reads were screened from raw data by filtering out the corrupted adapter sequences, poly-A tails and sequences with ≤18 nt and ≥ 30 nt. The clean read sequences were mapped into the Rfam (https://rfam.xfam.org) to filter the rRNA, tRNA, snRNA and snoRNA etc. The filtered reads were then compared against known plant miRNA database existing in the miRBase 21.0 with BLASTn. Only matching (0–3 mismatches) sequences in their sequences’ ends were considered as known VvmiRNAs, while other sequences have one base variation with the known VvmiRNAs in the middle sites of their sequences and thus can be considered as miRNA SNV. On the other hand, the identification criteria of novel miRNA as follow: 1) except for the identified known VvmiRNAs and VvmiRNA SNVs, the remaining sequences were mapped to the grape reference genome (http://genomes.cribi.unipd.it/DATA/V2/) and mRNA sequence. The reads mapped to genome but mRNA were used to predict the potential miRNA precursor with mireap, and then these reads werre processed by miRCat (http://srna-tools.cmp.uea.ac.uk) [70] using default parameters to generate the secondary structures; 2) The negative free energy of folding structure were less than -20kj; 3) The both arms of stem-loop structures contained the bubbles with less than 6 mismatched bases; 4) The first base of miRNAs possessed the “U” preference; 5) The length of miRNA is usually in the range of 19 -24 nt. In addition, as to the depth coverage and frequency filters for reliable calling of SNVs on miRNAs, here the depth coverage was required to be more than 4 (> 4), and the frequency filters was more than 0.05 (> 0.05).

### Identification of precise sequences of VvmiRNAs by miR-RACE

The cDNA was amplified with miR-Racer 5′ primer, 3′ primer and their corresponding gene-specific primers to generate 5′- and 3′-miR-RACE products, respectively, for the identification of precise sequences of miRNAs [16, 69]. The clone products of 5′- and 3′-miR-RACE were approximately 56 and 87 bp, respectively. 3′-miR-RACE was performed using common primer 1(CP1) (ATTCTAGAGGCCGAGGCGGCCGACATG) and miRNA specific primer 1(MGSP1), while 5′-miR-RACE was performed using CP2 (GGAGCACGAGGACACTGACATGGACT) and MGSP2. The design procedure of MGSP1 and MGSP2 primers as follow: MGSP1 primer consists of 10 bp adaptor sequence (GGAGTAGAAA) add 17 bp sequence intercepted from 5’end of miRNA, while MGSP2 primer includes 10 bp Poly(T) and 17 bp complementary sequence cut off from 3’end of miRNA [16, 69]. Here, the 17 nucleotides complimentary to the miRNA were sufficient for the accurate and efficient PCR amplification of the opposite ends. The primer specificity was validated by inspecting the specific band of PCR product. All specific primers are listed in Supplementary Table S7.

### Validation of potential target genes for VvmiRNAs with RLM-RACE and PPM-RACE

The mRNA library was ligated with 5′-adapter or 3′-PolyA tail [29] and then reverse transcribed as cDNA. RLM-RACE and PPM-RACE [28] were performed with their corresponding cDNA and primers, respectively (Supplementary Table S6). The products of RLM- or PPM-RACE were mapped into the target genes for validation of potential target genes and identification of cleavage sites and frequency. RLM-RACE was carried out using the common primer 1 (GGAGCACGAGGACACTGACATGGACT) and the specific primers (P1); and PPM-RACE was performed with the common primer 2 (ATTCTAGAGGCCGAGGCGGCCGACATG) and the specific primers (P2). P1 is the reverse primer at the downstream of the predicted cleavage site in target gene, while P2 is the forward primer at the upstream of corresponding cleavage site in target gene. The primer specificity was validated by inspecting the specific band of PCR product. P1 and P2 were listed in Supplementary Table S8.

### qRT-PCR analysis of VvmiRNAs and their target genes

For qRT-PCR expression analysis of VvmiRNAs and their target genes, the cDNA libraries for mRNA and miRNA from control and GA treatment at diverse stages were constructed using our developed methods [13], and then these cDNAs were used as templates to detect their corresponding expression levels using their primers with three replicates (Table 3), and the expression levels were normalized using 5.8S rRNA. The relative expression levels of the miRNAs and their targets were calculated by using the 2^−ΔΔCT^ method. miRNA qRT-PCR was amplified using common primer (qP1: ATTCTAGAGGCCGAGGCGGCCGACATG) and specific primer (qP2: miRNA sequence). The primer specificity was validated by inspecting the specific band of PCR product. The U6 gene was used as the reference gene for the normalization of all miRNAs’ relative expression values, and the actin gene was used as the referenced one for that of all target genes’ relative expression values. All primers were listed in Supplementary Table S9.

**Table 3:** Comparison of VvmiRNAs by miR-RACE and High throughput sequencing

ID	miR-RACE	High throughput seqencing	Consistence
VvmiR8	UCCAAGGAUGGAAAAGGCUUC	UCCAAGGAUGGAAAAGGCUUC	Yes
VvmiR16	UCUUUUCUUGAUAGAAGGCCU	UCUUUUCUUGAUAGAAGGCCU	Yes
VvmiR31	UUUCUUAGCAACCAAACAGAG	UUUCUUAGCAACCAAACAGAG	Yes
VvmiR38-5p	ACUCUCCCUCAAGGGCUUCUG	ACUCUCCCUCAAGGGCUUCUG	Yes
VvmiR44-3p	AGGUGCAGGUGAAGGUGCAGA	AGGUGCAGGUGAAGGUGCAGA	Yes
VvmiR53-3p	GGCAGCAGCAUACUACUUUG	GGCAGCAGCAUACUACUUUG	Yes

## Supplementary Information

**Additional file 1: Table S1.** Annotation of small RNA library in the stone hardening stage of grape berries. **Table S2.** Identified known VvmiRNAs in the stone hardening stage of grape berries. **Table S3.** Comparison of known VvmiRNAs in grape stone hardening stage and other two stages. **Table S4.** Comparison of novel VvmiRNAs in grape stone hardening stage and other two stages. **Table S5.** List of various SNP Edit types of vmiRNAs. **Table S6.** List of pathways invovled by target genes for VvmiRNAs. **Table S7.** List of miRNA specific primers for miR-RACE. **Table S8.** List of primers for RLM-RACE and PPM-RACE. **Table S9.** List of primers for qRT-PCR. **Figure S1.** Accumulation patterns of 3′- and 5′-end cleavage products of target genes.

## Data Availability

The RNA-seq data have been deposited into the NCBI GEO under the accession number GSE182618 (https://www.ncbi.nlm.nih.gov/geo/query/acc.cgi?acc=GSE182618). All data generated or analyzed during this study are included in this published article and its additional files.

## Abbreviations

Chr: Chromosome
CTAB: Cetyltrimethyl ammonium bromide
DEG: Differentially expressed gene
FPKM: Fragments Per Kilobase of transcript per Million mapped reads
GA: Gibberellin
GO: Gene ontology
HMW RNA: High-molecular-weight RNA
KEGG: Kyoto encyclopedia of genes and genomes
LMW RNA: Low-molecular-weight RNA
MB: Mature berry
miRNAs: MicroRNAs
PPM-RACE: Poly (A) polymerase -mediated 3′ rapid amplification of cDNA ends
qRT-PCR: Quantitative real-time PCR
RLM-RACE: RNA-ligase-mediated Rapid Amplification of cDNA Ends
SB: stone-hardening berry
TF: Transcription factor
Vv: *V. vinifera*
YB: Young berry
