# Supplementary material for: Identification of miRNAs-mediated seed and stone-hardening regulatory networks and their signal pathway of GA-induced seedless berries in grapevine (V. vinifera L.)
Source: BMC Plant Biol. 2021 Sep 29;21:442. doi: 10.1186/s12870-021-03188-y (PMC8480016; doi:10.1186/s12870-021-03188-y)
Supplement: Supplementary file 1 — Additional file 1: Table S1. Annotation of small RNA library in the stone hardening stage of grape berries. Table S2. Identified known VvmiRNAs in the stone hardening stage of grape berries. Table S3. Comparison of known VvmiRNAs in grape stone hardening stage and other two stages. Table S4. Comparison of novel VvmiRNAs in grape stone hardening stage and other two stages. Table S5. List of various SNP Edit types of vmiRNAs. Table S6. List of pathways invovled by target genes for VvmiRNAs. Table S7. List of miRNA specific primers for miR-RACE. Table S8. List of primers for RLM-RACE and PPM-RACE. Table S9. List of primers for qRT-PCR. Figure S1. Accumulation patterns of 3′- and 5′-end cleavage products of target genes. [file 12870_2021_3188_MOESM1_ESM.docx]

**
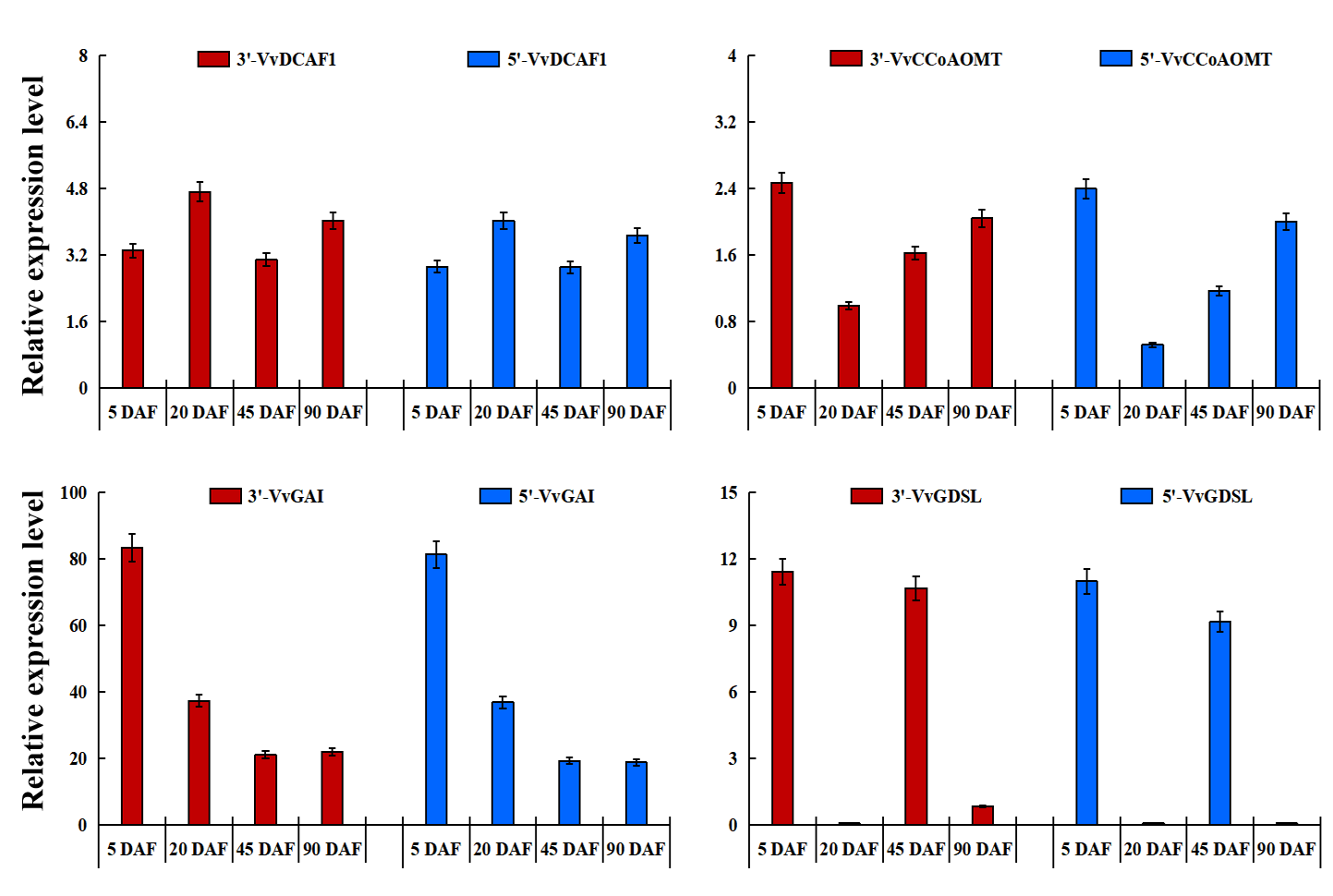
**

**Figure S1** Accumulation patterns of 3′- and 5′-end cleavage products of target genes.

**Table S1** Annotation of small RNA library in the stone hardening stage of grape berries

|  | Unique | % | Redundant | % |
| --- | --- | --- | --- | --- |
| exon_antisense | 66863 | 0.018615162 | 228850 | 0.019975288 |
| exon_sense | 98736 | 0.027488845 | 401889 | 0.035079084 |
| intron_antisense | 29798 | 0.008295987 | 71658 | 0.006254705 |
| intron_sense | 33929 | 0.009446089 | 94351 | 0.008235475 |
| **miRNA** | **1423** | **0.000396174** | **654386** | **0.057118412** |
| rRNA | 53824 | 0.014985006 | 396344 | 0.034595086 |
| repeat | 13632 | 0.003795251 | 24760 | 0.002161189 |
| snRNA | 3582 | 0.000997256 | 14759 | 0.001288247 |
| snoRNA | 1315 | 0.000366106 | 3499 | 0.000305412 |
| tRNA | 12703 | 0.003536611 | 219754 | 0.019181339 |
| **unannotation** | **3276052** | **0.912077513** | **9346406** | **0.815805764** |
| Map to Genome | 675466 | 0.188054814 | 3025532 | 0.264085087 |
| Total | 3591857 | 1 | 11456656 | 1 |

**Table S2** Identified known VvmiRNAs in the stone hardening stage of grape berries

| VvmiRNA ID | Unique Reads | Redundant Reads | Location in Precursors | Mature sequences | Sequences in miRbase 21.0 | Identity |
| --- | --- | --- | --- | --- | --- | --- |
| VvmiR156a | 32 | 65 | 5p | TGACAGAAGAGAGGGAGCAC | TGACAGAAGAGAGGGAGCAC | S |
| VvmiR156b | 9020 | 11417 | 5p | TGACAGAAGAGAGTGAGCAC | TGACAGAAGAGAGTGAGCAC | S |
| VvmiR156c | 9020 | 10753 | 5p | TGACAGAAGAGAGTGAGCAC | TGACAGAAGAGAGTGAGCAC | S |
| VvmiR156d | 9020 | 11570 | 5p | TGACAGAAGAGAGTGAGCAC | TGACAGAAGAGAGTGAGCAC | S |
| VvmiR156e | 23 | 25 | 5p | TGACAGAGGAGAGTGAGCAC | TGACAGAGGAGAGTGAGCAC | S |
| VvmiR156f | 29462 | 29990 | 5p | TTGACAGAAGATAGAGAGCAC | TTGACAGAAGATAGAGAGCAC | S |
| VvmiR156g | 29462 | 29952 | 5p | TTGACAGAAGATAGAGAGCAC | TTGACAGAAGATAGAGAGCAC | S |
| VvmiR156h | 1 | 1 | 5p | CAATGACAGAAGAGAGAGAGCA | TGACAGAAGAGAGAGAGCAT | D |
| VvmiR156i | 29462 | 29952 | 5p | TTGACAGAAGATAGAGAGCAC | TTGACAGAAGATAGAGAGCAC | S |
| VvmiR159c | 1204 | 1335 | 3p | TTTGGATTGAAGGGAGCTCTA | TTTGGATTGAAGGGAGCTCTA | S |
| VvmiR160a | 7 | 7 | 5p | TGCCTGGCTCCCTGAATGCCA | TGCCTGGCTCCCTGAATGCCATC | D |
| VvmiR160b | 7 | 7 | 5p | TGCCTGGCTCCCTGAATGCCA | TGCCTGGCTCCCTGAATGCCATC | D |
| VvmiR160c | 17 | 17 | 5p | TGCCTGGCTCCCTGTATGCCA | TGCCTGGCTCCCTGTATGCCA | S |
| VvmiR160d | 17 | 21 | 5p | TGCCTGGCTCCCTGTATGCCA | TGCCTGGCTCCCTGTATGCCA | S |
| VvmiR160e | 17 | 21 | 5p | TGCCTGGCTCCCTGTATGCCA | TGCCTGGCTCCCTGTATGCCA | S |
| VvmiR162 | 4068 | 4100 | 3p | TCGATAAACCTCTGCATCCAG | TCGATAAACCTCTGCATCCAG | S |
| VvmiR164a | 3783 | 3790 | 5p | TGGAGAAGCAGGGCACGTGCA | TGGAGAAGCAGGGCACGTGCA | S |
| VvmiR164c | 3783 | 3809 | 5p | TGGAGAAGCAGGGCACGTGCA | TGGAGAAGCAGGGCACGTGCA | S |
| VvmiR164d | 3783 | 3791 | 5p | TGGAGAAGCAGGGCACGTGCA | TGGAGAAGCAGGGCACGTGCA | S |
| VvmiR166a | 14630 | 17456 | 3p | TCTCGGACCAGGCTTCATTCC | TCGGACCAGGCTTCATTCC | D |
| VvmiR166b | 98605 | 101565 | 3p | TCGGACCAGGCTTCATTCCTC | TCGGACCAGGCTTCATTCC | D |
| VvmiR166c | 283511 | 287126 | 3p | TCGGACCAGGCTTCATTCCCC | TCGGACCAGGCTTCATTCCCC | S |
| VvmiR166d | 283511 | 287317 | 3p | TCGGACCAGGCTTCATTCCCC | TCGGACCAGGCTTCATTCCCC | S |
| VvmiR166e | 283511 | 287126 | 3p | TCGGACCAGGCTTCATTCCCC | TCGGACCAGGCTTCATTCCCC | S |
| VvmiR166f | 283511 | 287902 | 3p | TCGGACCAGGCTTCATTCCCC | TCGGACCAGGCTTCATTCCCC | S |
| VvmiR166g | 283511 | 288001 | 3p | TCGGACCAGGCTTCATTCCCC | TCGGACCAGGCTTCATTCCCC | S |
| VvmiR166h | 283511 | 301916 | 3p | TCGGACCAGGCTTCATTCCCC | TCGGACCAGGCTTCATTCCCC | S |
| VvmiR167a | 242 | 341 | 5p | TGAAGCTGCCAGCATGATCTGG | TGAAGCTGCCAGCATGATCTG | D |
| VvmiR167b | 6198 | 9550 | 5p | TGAAGCTGCCAGCATGATCTA | TGAAGCTGCCAGCATGATCTA | S |
| VvmiR167c | 5964 | 6468 | 5p | TGAAGCTGCCAGCATGATCTC | TGAAGCTGCCAGCATGATCTC | S |
| VvmiR167d | 6198 | 6285 | 5p | TGAAGCTGCCAGCATGATCTA | TGAAGCTGCCAGCATGATCTA | S |
| VvmiR167e | 6198 | 9501 | 5p | TGAAGCTGCCAGCATGATCTA | TGAAGCTGCCAGCATGATCTA | S |
| VvmiR168 | 54193 | 55816 | 5p | TCGCTTGGTGCAGGTCGGGAA | TCGCTTGGTGCAGGTCGGGAA | S |
| VvmiR169a | 96 | 101 | 5p | CAGCCAAGGATGACTTGCCGG | CAGCCAAGGATGACTTGCCGG | S |
| VvmiR169b | 48 | 50 | 5p | TGAGCCAAGGATGGCTTGCCGT | TGAGCCAAGGATGGCTTGCCG | D |
| VvmiR169c | 96 | 100 | 5p | CAGCCAAGGATGACTTGCCGG | CAGCCAAGGATGACTTGCCGG | S |
| VvmiR169d | 312 | 324 | 5p | CAGCCAAGAATGATTTGCCGG | CAGCCAAGAATGATTTGCCGG | S |
| VvmiR169e | 1 | 1 | 5p | TAGCCAAGGATGACTTGCCTGC | TAGCCAAGGATGACTTGCCTGC | S |
| VvmiR169f | 5 | 7 | 5p | CAGCCAAGGATGACTTGCCGA | CAGCCAAGGATGACTTGCCGA | S |
| VvmiR169g | 5 | 7 | 5p | CAGCCAAGGATGACTTGCCGA | CAGCCAAGGATGACTTGCCGA | S |
| VvmiR169h | 48 | 49 | 5p | TGAGCCAAGGATGGCTTGCCGT | TGAGCCAAGGATGGCTTGCCG | D |
| VvmiR169j | 96 | 101 | 5p | CAGCCAAGGATGACTTGCCGG | CAGCCAAGGATGACTTGCCGG | S |
| VvmiR169k | 96 | 101 | 5p | CAGCCAAGGATGACTTGCCGG | CAGCCAAGGATGACTTGCCGG | S |
| VvmiR169l | 268 | 292 | 5p | TGAGCCAAGGATGACTTGCCG | GAGCCAAGGATGACTTGCCGT | D |
| VvmiR169m | 268 | 276 | 5p | TGAGCCAAGGATGACTTGCCG | GAGCCAAGGATGACTTGCCGG | D |
| VvmiR169n | 57 | 74 | 5p | TAGAGCCAAGGATGACTTGCCG | GAGCCAAGGATGACTTGCCGG | D |
| VvmiR169o | 268 | 270 | 5p | TGAGCCAAGGATGACTTGCCG | GAGCCAAGGATGACTTGCCGC | D |
| VvmiR169p | 268 | 277 | 5p | TGAGCCAAGGATGACTTGCCG | GAGCCAAGGATGACTTGCCGG | D |
| VvmiR169q | 57 | 75 | 5p | TAGAGCCAAGGATGACTTGCCG | GAGCCAAGGATGACTTGCCGG | D |
| VvmiR169r | 55 | 57 | 5p | TGAGTCAAGGATGACTTGCCGA | TGAGTCAAGGATGACTTGCCG | D |
| VvmiR169s | 96 | 100 | 5p | CAGCCAAGGATGACTTGCCGG | CAGCCAAGGATGACTTGCCGG | S |
| VvmiR169t | 27 | 28 | 5p | CGAGTCAAGGATGACTTGCCGA | CGAGTCAAGGATGACTTGCCG | D |
| VvmiR169u | 17 | 17 | 5p | TGAGTCAAGGATGACTTGCCGT | TGAGTCAAGGATGACTTGCCG | D |
| VvmiR169v | 111 | 112 | 5p | AAGCCAAGGATGAATTGCCGG | AAGCCAAGGATGAATTGCCGG | S |
| VvmiR169w | 96 | 100 | 5p | CAGCCAAGGATGACTTGCCGG | CAGCCAAGGATGACTTGCCGG | S |
| VvmiR171a | 112 | 113 | 3p | TGATTGAGCCGTGCCAATATC | TGATTGAGCCGTGCCAATATC | S |
| VvmiR171b | 94 | 98 | 3p | TTGAGCCGCGTCAATATCTCC | TGATTGAGCCGCGTCAATATC | D |
| VvmiR171c | 112 | 121 | 3p | TGATTGAGCCGTGCCAATATC | TGATTGAGCCGTGCCAATATC | S |
| VvmiR171d | 112 | 121 | 3p | TGATTGAGCCGTGCCAATATC | TGATTGAGCCGTGCCAATATC | S |
| VvmiR171e | 1 | 1 | 3p | ATTGAGCCGCGCCAATATCAC | TGATTGAGCCGCGCCAATATC | D |
| VvmiR171f | 1 | 1 | 3p | ATTGAGCCGCGCCAATATCAC | TTGAGCCGCGCCAATATCACT | D |
| VvmiR171i | 112 | 113 | 3p | TGATTGAGCCGTGCCAATATC | TGATTGAGCCGTGCCAATATC | S |
| VvmiR172c | 6 | 8 | 3p | GGGAATCTTGATGATGCTGCA | GGAATCTTGATGATGCTGCAG | D |
| VvmiR172d | 1330 | 2747 | 3p | TGAGAATCTTGATGATGCTGC | TGAGAATCTTGATGATGCTGCAT | D |
| VvmiR2111* | 42 | 54 | 3p | GTCCTCTGGTTGCAGATTACT | GTCCTCTGGTTGCAGATTACT | S |
| VvmiR2111 | 270 | 272 | 5p | TAATCTGCATCCTGAGGTCTA | TAATCTGCATCCTGAGGTCTA | S |
| VvmiR2950* | 2908 | 3029 | 3p | TGGTGTGCACGGGATGGAATA | TGGTGTGCACGGGATGGAATA | S |
| VvmiR2950 | 1730 | 1773 | 5p | TTCCATCTCTTGCACACTGGA | TTCCATCTCTTGCACACTGGA | S |
| VvmiR319b | 2 | 4 | 3p | TTGGACTGAAGGGAGCTCCC | TTGGACTGAAGGGAGCTCCCT | D |
| VvmiR319c | 2 | 6 | 3p | TTGGACTGAAGGGAGCTCCC | TTGGACTGAAGGGAGCTCCCT | D |
| VvmiR319e | 87 | 100 | 3p | TTTGGACTGAAGGGAGCTCCT | TTTGGACTGAAGGGAGCTCCT | S |
| VvmiR319f | 2 | 6 | 3p | TTGGACTGAAGGGAGCTCCC | TTGGACTGAAGGGAGCTCCCT | D |
| VvmiR319g | 2 | 2 | 3p | TTGGACTGAAGGGAGCTCCC | TTGGACTGAAGGGAGCTCCCA | D |
| VvmiR3623 | 3603 | 4405 | 3p | TGGTGCTTGGACGAATTTGCTA | TGGTGCTTGGACGAATTTGCTA | S |
| VvmiR3623* | 10356 | 14830 | 5p | TCACAAGTTCATCCAAGCACCA | TCACAAGTTCATCCAAGCACCA | S |
| VvmiR3624 | 2635 | 2652 | 3p | TCAGGGCAGCAGCATACTACT | TCAGGGCAGCAGCATACTACT | S |
| VvmiR3625 | 9 | 17 | 3p | CGGGAGATGACTACTGGAAGC | CGGGAGATGACTACTGGAAGC | S |
| VvmiR3626* | 1 | 2 | 3p | TACTTCAATTTCACAGCGAC | CTTCAATTTCACAGCGACCAC | D |
| VvmiR3626 | 9 | 15 | 5p | CGGTAGTCGCTGTGAAATTGA | GGTAGTCGCTGTGAAATTGAA | D |
| VvmiR3627* | 1 | 1 | 3p | TCGCCGCTCTCCTGTGACAAG | TCGCCGCTCTCCTGTGACAAG | S |
| VvmiR3629a* | 1 | 2 | 5p | TTCCCGCATTTTCTCAGCAGC | CGCATTTTCTCAGCAGCCAAG | D |
| VvmiR3629a | 13 | 27 | 3p | GGCTGCTGAGAAAATGTAGGA | GGCTGCTGAGAAAATGTAGGA | S |
| VvmiR3629b | 13 | 27 | 3p | GGCTGCTGAGAAAATGTAGGA | GGCTGCTGAGAAAATGTAGGA | S |
| VvmiR3629c | 13 | 27 | 5p | GGCTGCTGAGAAAATGTAGGA | GGCTGCTGAGAAAATGTAGGA | S |
| VvmiR3630 | 6 | 20 | 5p | GCAAGTGACGATATCAGACA | TGCAAGTGACGATATCAGACA | D |
| VvmiR3631a* | 9 | 35 | 3p | TTGGATGATGTCAACAAGTTACTT | TATATTGGATGATGTCAACAA | D |
| VvmiR3631a | 74 | 120 | 5p | CATGTTGACATCATCCAATATA | CATGTTGACATCATCCAATATA | S |
| VvmiR3631b | 74 | 121 | 5p | CATGTTGACATCATCCAATATA | CATGTTGACATCATCCAATATA | S |
| VvmiR3631b* | 6 | 28 | 3p | TTTAGCTATGTTGGATGATGTCAA | TGTTGGATGATGTCAATAAGT | D |
| VvmiR3631c | 74 | 121 | 5p | CATGTTGACATCATCCAATATA | CATGTTGACATCATCCAATATA | S |
| VvmiR3631d | 74 | 120 | 3p | CATGTTGACATCATCCAATATA | CATGTTGACATCATCCAATATA | S |
| VvmiR3632* | 1 | 2 | 5p | TGGATTGGGGGCCGATGGAAAGG | GGATTGGGGGCCGATGGAAAGG | D |
| VvmiR3632 | 139 | 147 | 3p | TTTCCCAGACCCCCAATACCAA | TTTCCCAGACCCCCAATACCAA | S |
| VvmiR3633a* | 409 | 457 | 3p | TTCCTATACCACCCATTCCCTA | TTCCTATACCACCCATTCCCTA | S |
| VvmiR3633a | 3629 | 6164 | 5p | GGAATGGATGGTTAGGAGAG | GGAATGGATGGTTAGGAGAG | S |
| VvmiR3633b* | 210 | 220 | 3p | GTTCCCATGCCATCCATTCCTA | GTTCCCATGCCATCCATTCCTA | S |
| VvmiR3633b | 1001 | 1642 | 5p | GGAATGGGTGGCTGGGATCTA | GGAATGGGTGGCTGGGATCTA | S |
| VvmiR3634 | 120 | 124 | 3p | TTTCCGACTCGCACTCATGCCGT | TTTCCGACTCGCACTCATGCCGT | S |
| VvmiR3634* | 35 | 38 | 5p | GGCATATGTGTGACGGAAAGA | GGCATATGTGTGACGGAAAGA | S |
| VvmiR3635 | 1989 | 3021 | 5p | GGCATGTGTGGGGCATAATAG | GGCATGTGTGGGGCATAATAG | S |
| VvmiR3635* | 323 | 340 | 3p | ATTATGTCCCACACATGCCTC | ATTATGTCCCACACATGCCTC | S |
| VvmiR3636* | 1 | 2 | 5p | GGTTTGCTTCTTTGATAGATTCAG | TCGGTTTGCTTCTTTGATAGATTC | D |
| VvmiR3636 | 10577 | 12946 | 3p | GTCTGTCGGAGAAGCAAGTCGGAG | GTCTGTCGGAGAAGCAAGTCGGAG | S |
| VvmiR3637* | 573 | 1184 | 5p | TATGTATTGTGTTTTGTCGGAAAA | ATTTATGTATTGTGTTTTGTCGGA | D |
| VvmiR3637 | 482 | 1208 | 3p | TTTTTCGACAAGACACAATGCATA | TTTCGACAAGACACAATGCATAAA | D |
| VvmiR3638 | 1 | 1 | 3p | GCAACAAGCATGAAAAGGCACACC | GCAACAAGCATGAAAAGGCACACC | S |
| VvmiR3639* | 12 | 15 | 3p | TAAGAGCTTTTGGCTTCTCAGAAG | GAGCTTTTGGCTTCTCAGAAGTCA | D |
| VvmiR3639 | 154 | 493 | 5p | ATTGACTTCTGAAAGGCTAAAAGC | ATTGACTTCTGAAAGGCTAAAAGC | S |
| VvmiR3640 | 213 | 516 | 3p | GAAAAGGCATCATCAATCAGGTAA | ATCGAAAAGGCATCATCAATCAGG | D |
| VvmiR3640* | 250 | 313 | 5p | ACCTGATTGGTGATGCTTTTTTGG | ACCTGATTGGTGATGCTTTTTTGG | S |
| VvmiR390 | 88 | 178 | 5p | AAGCTCAGGAGGGATAGCGCC | AAGCTCAGGAGGGATAGCGCC | S |
| VvmiR394a | 4 | 5 | 5p | TTGGCATTCTGTCCACCTCC | TTGGCATTCTGTCCACCTCCAT | D |
| VvmiR394b | 4 | 4 | 5p | TTGGCATTCTGTCCACCTCC | TTGGCATTCTGTCCACCTCC | S |
| VvmiR394c | 4 | 5 | 5p | TTGGCATTCTGTCCACCTCC | TTGGCATTCTGTCCACCTCCAT | D |
| VvmiR395a | 132 | 145 | 3p | CTGAAGTGTTTGGGGGAACTC | CTGAAGTGTTTGGGGGAACTC | S |
| VvmiR395b | 132 | 145 | 3p | CTGAAGTGTTTGGGGGAACTC | CTGAAGTGTTTGGGGGAACTC | S |
| VvmiR395c | 132 | 145 | 3p | CTGAAGTGTTTGGGGGAACTC | CTGAAGTGTTTGGGGGAACTC | S |
| VvmiR395d | 132 | 145 | 3p | CTGAAGTGTTTGGGGGAACTC | CTGAAGTGTTTGGGGGAACTC | S |
| VvmiR395e | 132 | 145 | 3p | CTGAAGTGTTTGGGGGAACTC | CTGAAGTGTTTGGGGGAACTC | S |
| VvmiR395f | 132 | 145 | 3p | CTGAAGTGTTTGGGGGAACTC | CTGAAGTGTTTGGGGGAACTC | S |
| VvmiR395g | 132 | 147 | 3p | CTGAAGTGTTTGGGGGAACTC | CTGAAGTGTTTGGGGGAACTC | S |
| VvmiR395h | 132 | 145 | 3p | CTGAAGTGTTTGGGGGAACTC | CTGAAGTGTTTGGGGGAACTC | S |
| VvmiR395i | 132 | 145 | 3p | CTGAAGTGTTTGGGGGAACTC | CTGAAGTGTTTGGGGGAACTC | S |
| VvmiR395j | 132 | 145 | 3p | CTGAAGTGTTTGGGGGAACTC | CTGAAGTGTTTGGGGGAACTC | S |
| VvmiR395k | 132 | 145 | 3p | CTGAAGTGTTTGGGGGAACTC | CTGAAGTGTTTGGGGGAACTC | S |
| VvmiR395l | 132 | 145 | 3p | CTGAAGTGTTTGGGGGAACTC | CTGAAGTGTTTGGGGGAACTC | S |
| VvmiR395m | 132 | 145 | 3p | CTGAAGTGTTTGGGGGAACTC | CTGAAGTGTTTGGGGGAACTC | S |
| VvmiR396a | 551 | 753 | 5p | TTCCACAGCTTTCTTGAACTA | TTCCACAGCTTTCTTGAACTA | S |
| VvmiR396b | 608 | 1008 | 5p | TTCCACAGCTTTCTTGAACTT | TTCCACAGCTTTCTTGAACT | D |
| VvmiR396c | 85 | 257 | 5p | TTCCACAGCTTTCTTGAA | TTCCACAGCTTTCTTGAACTG | D |
| VvmiR396d | 85 | 262 | 5p | TTCCACAGCTTTCTTGAA | TTCCACAGCTTTCTTGAACTG | D |
| VvmiR397a | 67 | 71 | 5p | TCATTGAGTGCAGCGTTGATG | TCATTGAGTGCAGCGTTGATG | S |
| VvmiR398a | 1 | 1 | 3p | TGTGTTCTCAGGTCACCCCTT | TGTGTTCTCAGGTCACCCCTT | S |
| VvmiR398b | 4 | 4 | 3p | TGTGTTCTCAGGTCGCCCCTG | TGTGTTCTCAGGTCGCCCCTG | S |
| VvmiR398c | 4 | 4 | 3p | TGTGTTCTCAGGTCGCCCCTG | TGTGTTCTCAGGTCGCCCCTG | S |
| VvmiR399a | 8 | 10 | 3p | TGCCAAAGGAGAATTGCCCTG | TGCCAAAGGAGAATTGCCCTG | S |
| VvmiR399b | 5 | 5 | 3p | TGCCAAAGGAGAGTTGCCCTG | TGCCAAAGGAGAGTTGCCCTG | S |
| VvmiR399c | 5 | 5 | 3p | TGCCAAAGGAGAGTTGCCCTG | TGCCAAAGGAGAGTTGCCCTG | S |
| VvmiR399d | 5 | 8 | 3p | TCTGCCAAAGGAGATTTGCTC | TGCCAAAGGAGATTTGCTCGT | D |
| VvmiR399e | 65 | 65 | 3p | TGCCAAAGGAGATTTGCCCGG | TGCCAAAGGAGATTTGCCCGG | S |
| VvmiR399g | 32 | 34 | 3p | TGCCAAAGGAGATTTGCCCCT | TGCCAAAGGAGATTTGCCCCT | S |
| VvmiR399h | 8 | 10 | 3p | TGCCAAAGGAGAATTGCCCTG | TGCCAAAGGAGAATTGCCCTG | S |
| VvmiR399i | 352 | 366 | 3p | CGCCAAAGGAGAGTTGCCCTG | CGCCAAAGGAGAGTTGCCCTG | S |
| VvmiR403a | 368 | 444 | 3p | TTAGATTCACGCACAAACTCG | TTAGATTCACGCACAAACTCG | S |
| VvmiR403b | 368 | 444 | 3p | TTAGATTCACGCACAAACTCG | TTAGATTCACGCACAAACTCG | S |
| VvmiR403c | 368 | 444 | 3p | TTAGATTCACGCACAAACTCG | TTAGATTCACGCACAAACTCG | S |
| VvmiR403d | 368 | 444 | 3p | TTAGATTCACGCACAAACTCG | TTAGATTCACGCACAAACTCG | S |
| VvmiR403e | 368 | 444 | 3p | TTAGATTCACGCACAAACTCG | TTAGATTCACGCACAAACTCG | S |
| VvmiR403f | 368 | 445 | 3p | TTAGATTCACGCACAAACTCG | TTAGATTCACGCACAAACTCG | S |
| VvmiR408 | 27 | 30 | 3p | ATGCACTGCCTCTTCCCTGGC | ATGCACTGCCTCTTCCCTGGC | S |
| VvmiR447b | 10 | 13 | 5p | ACTCTTTCTCAAGGGCTTCTAG | ACTCTTTCTCAAGGGCTTCTAG | S |
| VvmiR447b* | 85 | 90 | 3p | CGAAGTCTTTGGGGAGAGTGG | CGAAGTCTTTGGGGAGAGTGG | S |
| VvmiR477 | 246 | 317 | 5p | TCCCTCAAAGGCTTCCAATTT | ATCTCCCTCAAAGGCTTCCAA | D |
| VvmiR479 | 33112 | 34737 | 5p | TGTGGTATTGGTTCGGCTCATC | TGTGGTATTGGTTCGGCTCATC | S |
| VvmiR482 | 1369 | 1394 | 3p | TCTTTCCTACTCCTCCCATTCC | TCTTTCCTACTCCTCCCATTCC | S |
| VvmiR535a | 3273 | 3427 | 5p | TGACAACGAGAGAGAGCACGC | TGACAACGAGAGAGAGCACGC | S |
| VvmiR535b | 3273 | 3427 | 5p | TGACAACGAGAGAGAGCACGC | TGACAACGAGAGAGAGCACGC | S |
| VvmiR535c | 3273 | 3427 | 5p | TGACAACGAGAGAGAGCACGC | TGACAACGAGAGAGAGCACGC | S |
| VvmiR828a | 7 | 9 | 5p | TCTTGCTCAAATGAGTATTCCA | TCTTGCTCAAATGAGTATTCCA | S |
| VvmiR845d | 1 | 1 | 3p | ATGGCTCTGATACCAATTGATGGG | TGGCTCTGATACCAATTGATG | D |
| VvmiR845e | 1 | 1 | 3p | ATGGCTCTGATACCAATTGATGGG | TGGCTCTGATACCAATTGATG | D |

**Table S3** Comparison of known VvmiRNAs in grape stone hardening stage and other two stages

| miRNA ID | SB | YB | MB | log2Fold  (YB/SB) | log2Fold  (MB/SB) | pvalue  (YB/SB) | qvalue  (YB/SB) | pvalue  (MB/SB) | qvalue  (MB/SB) | Diff  (YB/SB) | Diff  (MB/SB) |
| --- | --- | --- | --- | --- | --- | --- | --- | --- | --- | --- | --- |
| **VvmiR156a** | 63.8522 | 597.706 | 101.146 | 3.359019123 | 0.73598543 | 2.66E-117 | 1.81E-116 | 0.0012192 | 0.00197476 | Up | noDEG |
| **VvmiR156b** | 11215.4 | 11368.6 | 26498.2 | 0.151967974 | 1.312770921 | 2.02E-15 | 7.08E-15 | 0 | 0 | noDEG | Up |
| **VvmiR156c** | 10563.1 | 10771.9 | 25412.7 | 0.160631359 | 1.338866109 | 3.47E-16 | 1.25E-15 | 0 | 0 | noDEG | Up |
| **VvmiR156d** | 11365.7 | 11526.2 | 26641.5 | 0.152618554 | 1.301341505 | 9.88E-16 | 3.51E-15 | 0 | 0 | noDEG | Up |
| **VvmiR156e** | 24.5585 | 10.1651 | 39.0564 | -1.140213436 | 0.741687461 | 0.026245001 | 0.035247647 | 0.043034558 | 0.05408715 | Down | noDEG |
| **VvmiR156f** | 29460.4 | 21557.1 | 59860.4 | -0.318226273 | 1.09517761 | 4.57E-137 | 3.30E-136 | 0 | 0 | noDEG | Up |
| **VvmiR156g** | 29423.1 | 21515.4 | 59842.4 | -0.319189152 | 1.096572477 | 1.15E-137 | 8.73E-137 | 0 | 0 | noDEG | Up |
| **VvmiR156i** | 29423.1 | 21515.4 | 59842.4 | -0.319189152 | 1.096572477 | 1.15E-137 | 8.73E-137 | 0 | 0 | noDEG | Up |
| VvmiR159c | 1311.43 | 1546.11 | 856.236 | 0.369894385 | -0.542702507 | 7.54E-12 | 2.29E-11 | 4.63E-18 | 1.69E-17 | noDEG | noDEG |
| **VvmiR160a** | 6.87639 | 13.2146 | 19.0275 | 1.07479922 | 1.540714092 | 0.103670876 | 0.129099581 | 0.0106374 | 0.01553398 | Up | Up |
| **VvmiR160b** | 6.87639 | 13.2146 | 19.0275 | 1.07479922 | 1.540714092 | 0.103670876 | 0.129099581 | 0.0106374 | 0.01553398 | Up | Up |
| **VvmiR160c** | 16.6998 | 10.1651 | 43.0621 | -0.583820053 | 1.438943363 | 0.301409807 | 0.345536801 | 0.000259389 | 0.000473487 | noDEG | Up |
| **VvmiR160d** | 20.6292 | 12.1981 | 54.078 | -0.625639889 | 1.462711258 | 0.221721268 | 0.26064943 | 3.41E-05 | 6.74E-05 | noDEG | Up |
| **VvmiR160e** | 20.6292 | 12.1981 | 54.078 | -0.625639889 | 1.462711258 | 0.221721268 | 0.26064943 | 3.41E-05 | 6.74E-05 | noDEG | Up |
| VvmiR162 | 4027.6 | 5979.1 | 2708.91 | 0.702397704 | -0.499853951 | 6.35E-129 | 4.44E-128 | 4.18E-45 | 2.14E-44 | noDEG | noDEG |
| **VvmiR164a** | 3723.07 | 11728.5 | 493.713 | 1.787838019 | -2.842397043 | 0 | 0 | 0 | 0 | Up | Down |
| **VvmiR164c** | 3741.74 | 11804.7 | 495.715 | 1.789971552 | -2.84377068 | 0 | 0 | 0 | 0 | Up | Down |
| **VvmiR164d** | 3724.06 | 11728.5 | 493.713 | 1.787457543 | -2.842777519 | 0 | 0 | 0 | 0 | Up | Down |
| VvmiR166a | 17147.8 | 16874 | 8745.62 | 0.10917505 | -0.899032549 | 2.38E-12 | 7.52E-12 | 0 | 0 | noDEG | noDEG |
| **VvmiR166b** | 99771.5 | 188419 | 37646.3 | 1.049633108 | -1.333764365 | 0 | 0 | 0 | 0 | Up | Down |
| VvmiR166c | 282056 | 231501 | 246372 | -0.152568552 | -0.122790545 | 0 | 0 | 5.28E-238 | 3.85E-237 | noDEG | noDEG |
| VvmiR166d | 282243 | 231699 | 246656 | -0.152299896 | -0.122085878 | 0 | 0 | 1.52E-235 | 1.06E-234 | noDEG | noDEG |
| VvmiR166e | 282056 | 231501 | 246372 | -0.152568552 | -0.122790545 | 0 | 0 | 5.28E-238 | 3.85E-237 | noDEG | noDEG |
| VvmiR166f | 282818 | 232795 | 246824 | -0.14842077 | -0.124035922 | 0 | 0 | 1.96E-243 | 1.50E-242 | noDEG | noDEG |
| VvmiR166g | 282915 | 233033 | 246856 | -0.147444069 | -0.124345149 | 0 | 0 | 1.05E-244 | 8.35E-244 | noDEG | noDEG |
| VvmiR166h | 296585 | 241127 | 254455 | -0.166261657 | -0.148677844 | 0 | 0 | 0 | 0 | noDEG | noDEG |
| VvmiR167a | 334.978 | 204.318 | 376.543 | -0.580861847 | 0.241102258 | 4.07E-06 | 8.80E-06 | 0.025912046 | 0.033481858 | noDEG | noDEG |
| VvmiR167b | 9381.36 | 13146.5 | 7070.2 | 0.619199298 | -0.335691274 | 7.86E-226 | 7.26E-225 | 4.57E-50 | 2.50E-49 | noDEG | noDEG |
| **VvmiR167c** | 6353.78 | 10520.9 | 1539.22 | 0.859953939 | -1.973061488 | 0 | 0 | 0 | 0 | noDEG | Down |
| VvmiR167d | 6174.02 | 4932.09 | 4876.04 | -0.191618655 | -0.26814604 | 2.76E-12 | 8.62E-12 | 1.87E-22 | 7.28E-22 | noDEG | noDEG |
| VvmiR167e | 9333.23 | 13103.8 | 7036.15 | 0.621927183 | -0.335234446 | 7.42E-227 | 7.14E-226 | 1.08E-49 | 5.75E-49 | noDEG | noDEG |
| VvmiR168 | 54830.4 | 51490.2 | 34009.1 | 0.041712149 | -0.616700796 | 1.80E-06 | 3.97E-06 | 0 | 0 | noDEG | noDEG |
| **VvmiR169a** | 99.2165 | 183.988 | 150.217 | 1.023349194 | 0.670748807 | 5.43E-09 | 1.51E-08 | 0.000280046 | 0.000503208 | Up | noDEG |
| VvmiR169b | 49.1171 | 60.9904 | 64.0925 | 0.444749008 | 0.456284989 | 0.106624605 | 0.131712747 | 0.093846821 | 0.109567354 | noDEG | noDEG |
| **VvmiR169c** | 98.2341 | 183.988 | 150.217 | 1.037704468 | 0.685104081 | 3.62E-09 | 1.03E-08 | 0.000214222 | 0.000394169 | Up | noDEG |
| VvmiR169d | 318.279 | 507.237 | 404.584 | 0.80476045 | 0.418502566 | 2.55E-15 | 8.78E-15 | 0.000101671 | 0.000196508 | noDEG | noDEG |
| VvmiR169e | 0.001 | 2.03301 | 5.00723 | 1.181714512 | 2.422069483 | 0.490890014 | 0.542562647 | 0.077028115 | 0.093737918 | noDEG | noDEG |
| VvmiR169f | 6.87639 | 17.2806 | 4.00578 | 1.461822536 | -0.70721344 | 0.01794181 | 0.024308258 | 0.42706647 | 0.479147747 | noDEG | noDEG |
| VvmiR169g | 6.87639 | 17.2806 | 5.00723 | 1.461822536 | -0.385285418 | 0.01794181 | 0.024308258 | 0.647303989 | 0.692464733 | noDEG | noDEG |
| VvmiR169h | 48.1347 | 60.9904 | 63.091 | 0.473895312 | 0.462711105 | 0.087148847 | 0.109409694 | 0.092212722 | 0.108208806 | noDEG | noDEG |
| **VvmiR169i** | 0.001 | 1.01651 | 21.0304 | NA | 5.466755126 | NA | NA | 1.39E-06 | 3.15E-06 | noDEG | Up |
| **VvmiR169j** | 99.2165 | 182.971 | 150.217 | 1.01535646 | 0.670748807 | 7.48E-09 | 2.04E-08 | 0.000280046 | 0.000503208 | Up | noDEG |
| **VvmiR169k** | 99.2165 | 182.971 | 150.217 | 1.01535646 | 0.670748807 | 7.48E-09 | 2.04E-08 | 0.000280046 | 0.000503208 | Up | noDEG |
| **VvmiR169l** | 286.844 | 409.652 | 666.963 | 0.646526057 | 1.289694987 | 4.34E-09 | 1.22E-08 | 6.42E-40 | 3.08E-39 | noDEG | Up |
| **VvmiR169m** | 271.126 | 401.52 | 624.902 | 0.698899018 | 1.277019133 | 4.79E-10 | 1.40E-09 | 5.78E-37 | 2.61E-36 | noDEG | Up |
| **VvmiR169n** | 72.6933 | 74.205 | 187.27 | 0.162085805 | 1.437582004 | 0.495746368 | 0.542736545 | 2.69E-14 | 8.98E-14 | noDEG | Up |
| **VvmiR169o** | 265.232 | 402.537 | 628.908 | 0.734255369 | 1.317946307 | 7.56E-11 | 2.27E-10 | 5.74E-39 | 2.69E-38 | noDEG | Up |
| **VvmiR169p** | 272.109 | 404.57 | 636.919 | 0.704597229 | 1.299282283 | 3.09E-10 | 9.15E-10 | 1.28E-38 | 5.88E-38 | noDEG | Up |
| **VvmiR169q** | 73.6756 | 74.205 | 188.272 | 0.142720507 | 1.425911519 | 0.547288959 | 0.590765185 | 3.30E-14 | 1.08E-13 | noDEG | Up |
| VvmiR169r | 55.9935 | 33.5447 | 54.078 | -0.606781577 | 0.022138703 | 0.050122464 | 0.065785734 | 0.93583517 | 0.960902184 | noDEG | noDEG |
| **VvmiR169s** | 98.2341 | 180.938 | 151.218 | 1.013592184 | 0.694689698 | 9.50E-09 | 2.54E-08 | 0.000170146 | 0.000319458 | Up | noDEG |
| **VvmiR169t** | 27.5056 | 11.1816 | 19.0275 | -1.166208593 | -0.459286118 | 0.016622274 | 0.022992487 | 0.281536845 | 0.325394343 | Down | noDEG |
| VvmiR169u | 16.6998 | 12.1981 | 31.0448 | -0.320784937 | 0.966874983 | 0.552312018 | 0.593414307 | 0.023718734 | 0.031173193 | noDEG | noDEG |
| **VvmiR169v** | 110.022 | 22.3632 | 50.0723 | -2.166208068 | -1.063357008 | 7.34E-14 | 2.42E-13 | 7.09E-06 | 1.52E-05 | Down | Down |
| **VvmiR169w** | 98.2341 | 180.938 | 151.218 | 1.013592184 | 0.694689698 | 9.50E-09 | 2.54E-08 | 0.000170146 | 0.000319458 | Up | noDEG |
| VvmiR169x | 0.001 | 3.04952 | 6.00867 | NA | 3.659400101 | NA | NA | 0.018191044 | 0.024467486 | noDEG | noDEG |
| **VvmiR171a** | 111.005 | 440.148 | 54.078 | 2.119758561 | -0.965150523 | 6.93E-54 | 3.77E-53 | 3.12E-05 | 6.27E-05 | Up | noDEG |
| VvmiR171b | 96.2695 | 113.849 | 50.0723 | 0.374359754 | -0.870712305 | 0.06032361 | 0.077847787 | 0.000375725 | 0.000654672 | noDEG | noDEG |
| **VvmiR171c** | 118.863 | 458.445 | 59.0853 | 2.079835001 | -0.936078998 | 9.37E-55 | 5.35E-54 | 2.66E-05 | 5.44E-05 | Up | noDEG |
| **VvmiR171d** | 118.863 | 458.445 | 59.0853 | 2.079835001 | -0.936078998 | 9.37E-55 | 5.35E-54 | 2.66E-05 | 5.44E-05 | Up | noDEG |
| VvmiR171e | 0.001 | 7.11555 | 0.001 | 2.989069637 | 0 | 0.017154556 | 0.023517522 | 0 | 0 | noDEG | ---- |
| VvmiR171f | 0.001 | 7.11555 | 0.001 | 2.989069637 | 0 | 0.017154556 | 0.023517522 | 0 | 0 | noDEG | ---- |
| **VvmiR171i** | 111.005 | 440.148 | 54.078 | 2.119758561 | -0.965150523 | 6.93E-54 | 3.77E-53 | 3.12E-05 | 6.27E-05 | Up | noDEG |
| **VvmiR172c** | 7.85873 | 27.4457 | 1.00145 | 1.936601957 | -2.89985901 | 0.000273206 | 0.000504885 | 0.017267929 | 0.023500731 | Up | noDEG |
| VvmiR172d | 2698.49 | 2226.15 | 2117.06 | -0.145211334 | -0.277740489 | 0.000419648 | 0.000757334 | 2.74E-11 | 8.63E-11 | noDEG | noDEG |
| **VvmiR2111** | 267.197 | 85.3866 | 415.6 | -1.513430522 | 0.709646054 | 7.40E-20 | 2.80E-19 | 2.14E-10 | 6.48E-10 | Down | noDEG |
| **VvmiR2111*** | 53.0464 | 20.3301 | 88.1272 | -1.251244889 | 0.804685386 | 0.000437636 | 0.000783673 | 0.001107196 | 0.001872465 | Down | noDEG |
| VvmiR2950 | 1741.69 | 1587.78 | 1381.99 | -0.001083407 | -0.26138317 | 0.982708006 | 0.991290609 | 4.45E-07 | 1.04E-06 | noDEG | noDEG |
| **VvmiR2950*** | 2975.51 | 2991.58 | 7934.45 | 0.14016033 | 1.487346456 | 0.000172024 | 0.00032307 | 0 | 0 | noDEG | Up |
| **VvmiR319b** | 3.92937 | 36.5943 | 4.00578 | 3.351639903 | 0.100141534 | 1.26E-08 | 3.30E-08 | 0.922107754 | 0.95105284 | Up | noDEG |
| **VvmiR319c** | 5.89405 | 39.6438 | 4.00578 | 2.882154436 | -0.484821089 | 3.28E-08 | 8.46E-08 | 0.600358558 | 0.649799851 | Up | noDEG |
| **VvmiR319e** | 98.2341 | 34.5612 | 213.308 | -1.374678753 | 1.190994632 | 2.66E-07 | 6.61E-07 | 2.15E-12 | 6.96E-12 | Down | Up |
| **VvmiR319f** | 5.89405 | 39.6438 | 4.00578 | 2.882154436 | -0.484821089 | 3.28E-08 | 8.46E-08 | 0.600358558 | 0.649799851 | Up | noDEG |
| **VvmiR319g** | 1.96468 | 21.3467 | 3.00434 | 3.574031844 | 0.685103307 | 8.19E-06 | 1.70E-05 | 0.600644333 | 0.64555232 | Up | noDEG |
| VvmiR3623 | 4327.21 | 4786.73 | 5141.42 | 0.277993187 | 0.32108229 | 3.37E-20 | 1.30E-19 | 2.73E-27 | 1.18E-26 | noDEG | noDEG |
| **VvmiR3623*** | 14568.1 | 4418.76 | 10389 | -1.588711915 | -0.415405136 | 0 | 0 | 1.40E-113 | 8.27E-113 | Down | noDEG |
| **VvmiR3624** | 2605.17 | 828.453 | 4510.51 | -1.520494063 | 0.864267453 | 9.22E-180 | 7.61E-179 | 4.87E-136 | 3.11E-135 | Down | noDEG |
| **VvmiR3625** | 16.6998 | 37.6108 | 14.0202 | 1.303705257 | -0.179966637 | 0.001371581 | 0.002312666 | 0.730022847 | 0.773756934 | Up | noDEG |
| VvmiR3625* | 0.001 | 1.01651 | 0.001 | NA | 0 | NA | NA | 0 | 0 | noDEG | ---- |
| VvmiR3626 | 14.7351 | 29.4787 | 26.0376 | 1.132805206 | 0.893690174 | 0.01121032 | 0.015790147 | 0.052513099 | 0.064588303 | noDEG | noDEG |
| VvmiR3626* | 1.96468 | 0.001 | 0.001 | NA | NA | NA | NA | NA | NA | noDEG | noDEG |
| VvmiR3627 | 0.001 | 2.03301 | 1.00145 | NA | NA | NA | NA | NA | NA | noDEG | ---- |
| VvmiR3628* | 0.001 | 0.001 | 1.00145 | 0 | NA | 0 | 0 | NA | NA | ---- | noDEG |
| **VvmiR3629a** | 26.5232 | 60.9904 | 44.0636 | 1.333717576 | 0.804685278 | 3.36E-05 | 6.51E-05 | 0.021088273 | 0.028036433 | Up | noDEG |
| VvmiR3629a* | 1.96468 | 2.03301 | 10.0145 | 0.181714219 | 2.422068902 | 0.899751274 | 0.923744641 | 0.012398044 | 0.017178013 | noDEG | noDEG |
| **VvmiR3629b** | 26.5232 | 60.9904 | 44.0636 | 1.333717576 | 0.804685278 | 3.36E-05 | 6.51E-05 | 0.021088273 | 0.028036433 | Up | noDEG |
| **VvmiR3629c** | 26.5232 | 60.9904 | 44.0636 | 1.333717576 | 0.804685278 | 3.36E-05 | 6.51E-05 | 0.021088273 | 0.028036433 | Up | noDEG |
| VvmiR3630 | 19.6468 | 29.4787 | 10.0145 | 0.717767461 | -0.899859193 | 0.08421548 | 0.106888879 | 0.098515533 | 0.114437236 | noDEG | noDEG |
| VvmiR3630* | 0.001 | 2.03301 | 2.00289 | NA | NA | NA | NA | NA | NA | noDEG | noDEG |
| VvmiR3631a | 117.881 | 53.8749 | 88.1272 | -0.997255926 | -0.347318251 | 1.27E-05 | 2.53E-05 | 0.085629554 | 0.102844895 | noDEG | noDEG |
| VvmiR3631a* | 34.382 | 62.0069 | 33.0477 | 0.983168714 | 0.015252232 | 0.00105231 | 0.001841543 | 0.965375217 | 0.965375217 | noDEG | noDEG |
| **VvmiR3631b** | 118.863 | 52.8584 | 89.1286 | -1.036708876 | -0.342988496 | 6.00E-06 | 1.28E-05 | 0.088040508 | 0.104647633 | Down | noDEG |
| VvmiR3631b* | 27.5056 | 46.7593 | 28.0405 | 0.897921604 | 0.100140912 | 0.008365322 | 0.01192833 | 0.795867643 | 0.828278543 | noDEG | noDEG |
| **VvmiR3631c** | 118.863 | 52.8584 | 89.1286 | -1.036708876 | -0.342988496 | 6.00E-06 | 1.28E-05 | 0.088040508 | 0.104647633 | Down | noDEG |
| VvmiR3631d | 117.881 | 53.8749 | 88.1272 | -0.997255926 | -0.347318251 | 1.27E-05 | 2.53E-05 | 0.085629554 | 0.102844895 | noDEG | noDEG |
| **VvmiR3632** | 144.404 | 350.695 | 29.0419 | 1.412494369 | -2.24155029 | 2.71E-25 | 1.10E-24 | 9.34E-19 | 3.52E-18 | Up | Down |
| VvmiR3632* | 1.96468 | 7.11555 | 2.00289 | 1.989069343 | 0.100140807 | 0.059281606 | 0.076932871 | 0.944878431 | 0.961601943 | noDEG | noDEG |
| VvmiR3633a | 6055.15 | 10864.4 | 3443.97 | 0.975768027 | -0.741736429 | 0 | 0 | 1.37E-133 | 8.53E-133 | noDEG | noDEG |
| VvmiR3633a* | 448.93 | 577.376 | 278.402 | 0.495411254 | -0.616967799 | 4.20E-08 | 1.07E-07 | 1.29E-08 | 3.06E-08 | noDEG | noDEG |
| VvmiR3633b | 1613 | 2084.86 | 2169.13 | 0.502588347 | 0.499720674 | 4.63E-26 | 1.98E-25 | 3.01E-26 | 1.26E-25 | noDEG | noDEG |
| VvmiR3633b* | 216.115 | 251.077 | 157.227 | 0.348722345 | -0.386597637 | 0.009050268 | 0.012825839 | 0.010078783 | 0.014859745 | noDEG | noDEG |
| **VvmiR3634** | 121.81 | 402.537 | 72.1041 | 1.856875174 | -0.684129757 | 7.83E-42 | 4.02E-41 | 0.00116766 | 0.001904694 | Up | noDEG |
| VvmiR3634* | 37.329 | 47.7758 | 56.0809 | 0.488375858 | 0.659568743 | 0.119621739 | 0.14698203 | 0.028738909 | 0.036927089 | noDEG | noDEG |
| **VvmiR3635** | 2967.65 | 7864.72 | 1272.84 | 1.538462697 | -1.148920562 | 0 | 0 | 3.29E-136 | 2.16E-135 | Up | Down |
| **VvmiR3635*** | 333.996 | 372.042 | 153.221 | 0.288023311 | -1.051862102 | 0.007999938 | 0.011478171 | 8.78E-15 | 3.01E-14 | noDEG | Down |
| **VvmiR3636** | 12717.4 | 14799.3 | 887.28 | 0.35111987 | -3.768914535 | 5.10E-91 | 3.18E-90 | 0 | 0 | noDEG | Down |
| VvmiR3636* | 1.96468 | 4.06603 | 0.001 | 1.181714219 | NA | 0.329935761 | 0.371781272 | NA | NA | noDEG | noDEG |
| VvmiR3637 | 1186.67 | 1083.6 | 693 | 0.001302565 | -0.703634933 | 0.982834414 | 0.987107607 | 2.78E-25 | 1.12E-24 | noDEG | noDEG |
| VvmiR3637* | 1163.09 | 1404.81 | 708.022 | 0.404803505 | -0.643745564 | 1.25E-12 | 4.02E-12 | 1.94E-21 | 7.44E-21 | noDEG | noDEG |
| VvmiR3638 | 0.001 | 3.04952 | 1.00145 | 1.766677013 | 0.1001411 | 0.254379316 | 0.295284533 | 0.961007539 | 0.96943743 | noDEG | noDEG |
| VvmiR3638* | 0.001 | 1.01651 | 1.00145 | NA | NA | NA | NA | NA | NA | noDEG | noDEG |
| **VvmiR3639** | 484.294 | 486.907 | 199.288 | 0.140152545 | -1.208677906 | 0.129847396 | 0.157040568 | 1.56E-25 | 6.41E-25 | noDEG | Down |
| **VvmiR3639*** | 14.7351 | 13.2146 | 3.00434 | -0.024736523 | -2.221787043 | 0.963857156 | 0.976539487 | 0.004964017 | 0.007561086 | noDEG | Down |
| **VvmiR3640** | 506.888 | 1102.91 | 218.315 | 1.253966491 | -1.142901866 | 1.81E-63 | 1.10E-62 | 1.63E-24 | 6.45E-24 | Up | Down |
| **VvmiR3640*** | 307.473 | 572.294 | 52.0752 | 1.028687052 | -2.489437625 | 5.00E-25 | 1.99E-24 | 6.92E-43 | 3.46E-42 | Up | Down |
| **VvmiR390** | 174.857 | 2778.11 | 20.0289 | 4.122250617 | -3.053664637 | 0 | 0 | 1.10E-30 | 4.86E-30 | Up | Down |
| VvmiR393a | 0.001 | 4.06603 | 0.001 | NA | 0 | NA | NA | 0 | 0 | noDEG | ---- |
| VvmiR393b | 0.001 | 4.06603 | 0.001 | NA | 0 | NA | NA | 0 | 0 | noDEG | ---- |
| **VvmiR394a** | 4.91171 | 27.4457 | 31.0448 | 2.614673825 | 2.732409401 | 1.25E-05 | 2.54E-05 | 2.52E-06 | 5.48E-06 | Up | Up |
| **VvmiR394b** | 3.92937 | 26.4292 | 29.0419 | 2.882154195 | 2.958122417 | 6.43E-06 | 1.35E-05 | 2.14E-06 | 4.74E-06 | Up | Up |
| **VvmiR394c** | 4.91171 | 27.4457 | 31.0448 | 2.614673825 | 2.732409401 | 1.25E-05 | 2.54E-05 | 2.52E-06 | 5.48E-06 | Up | Up |
| VvmiR395a | 142.44 | 179.922 | 253.366 | 0.469411345 | 0.903225554 | 0.003583841 | 0.005537574 | 1.04E-09 | 2.90E-09 | noDEG | noDEG |
| VvmiR395b | 142.44 | 179.922 | 253.366 | 0.469411345 | 0.903225554 | 0.003583841 | 0.005537574 | 1.04E-09 | 2.90E-09 | noDEG | noDEG |
| VvmiR395c | 142.44 | 179.922 | 253.366 | 0.469411345 | 0.903225554 | 0.003583841 | 0.005537574 | 1.04E-09 | 2.90E-09 | noDEG | noDEG |
| VvmiR395d | 142.44 | 179.922 | 253.366 | 0.469411345 | 0.903225554 | 0.003583841 | 0.005537574 | 1.04E-09 | 2.90E-09 | noDEG | noDEG |
| VvmiR395e | 142.44 | 179.922 | 253.366 | 0.469411345 | 0.903225554 | 0.003583841 | 0.005537574 | 1.04E-09 | 2.90E-09 | noDEG | noDEG |
| VvmiR395f | 142.44 | 179.922 | 253.366 | 0.469411345 | 0.903225554 | 0.003583841 | 0.005537574 | 1.04E-09 | 2.90E-09 | noDEG | noDEG |
| VvmiR395g | 144.404 | 186.021 | 254.367 | 0.497741864 | 0.889153551 | 0.001778269 | 0.002955253 | 1.60E-09 | 4.08E-09 | noDEG | noDEG |
| VvmiR395h | 142.44 | 179.922 | 253.366 | 0.469411345 | 0.903225554 | 0.003583841 | 0.005537574 | 1.04E-09 | 2.90E-09 | noDEG | noDEG |
| VvmiR395i | 142.44 | 179.922 | 253.366 | 0.469411345 | 0.903225554 | 0.003583841 | 0.005537574 | 1.04E-09 | 2.90E-09 | noDEG | noDEG |
| VvmiR395j | 142.44 | 179.922 | 253.366 | 0.469411345 | 0.903225554 | 0.003583841 | 0.005537574 | 1.04E-09 | 2.90E-09 | noDEG | noDEG |
| VvmiR395k | 142.44 | 179.922 | 253.366 | 0.469411345 | 0.903225554 | 0.003583841 | 0.005537574 | 1.04E-09 | 2.90E-09 | noDEG | noDEG |
| VvmiR395l | 142.44 | 179.922 | 253.366 | 0.469411345 | 0.903225554 | 0.003583841 | 0.005537574 | 1.04E-09 | 2.90E-09 | noDEG | noDEG |
| VvmiR395m | 142.44 | 179.922 | 253.366 | 0.469411345 | 0.903225554 | 0.003583841 | 0.005537574 | 1.04E-09 | 2.90E-09 | noDEG | noDEG |
| **VvmiR396a** | 739.703 | 494.023 | 287.415 | -0.449978755 | -1.291457603 | 5.76E-08 | 1.45E-07 | 4.26E-42 | 2.08E-41 | noDEG | Down |
| VvmiR396b | 990.2 | 888.427 | 610.882 | -0.02407591 | -0.624473374 | 0.717604863 | 0.750075671 | 1.36E-17 | 4.88E-17 | noDEG | noDEG |
| VvmiR396c | 252.462 | 189.07 | 155.224 | -0.28475116 | -0.629358731 | 0.039008024 | 0.051786515 | 1.42E-05 | 2.97E-05 | noDEG | noDEG |
| VvmiR396d | 257.373 | 195.169 | 163.236 | -0.266745459 | -0.584553057 | 0.05018254 | 0.065492468 | 4.08E-05 | 7.96E-05 | noDEG | noDEG |
| **VvmiR397a** | 69.7462 | 234.813 | 87.1257 | 1.883716377 | 0.393337773 | 1.46E-25 | 6.03E-25 | 0.088675075 | 0.104591114 | Up | noDEG |
| VvmiR398a | 0.001 | 2.03301 | 1.00145 | 1.181714512 | 0.1001411 | 0.490890014 | 0.542562647 | 0.961007539 | 0.96943743 | noDEG | noDEG |
| VvmiR398b | 3.92937 | 2.03301 | 7.01012 | -0.818285414 | 0.907496302 | 0.499916703 | 0.543438863 | 0.308134104 | 0.353470543 | noDEG | noDEG |
| VvmiR398c | 3.92937 | 2.03301 | 7.01012 | -0.818285414 | 0.907496302 | 0.499916703 | 0.543438863 | 0.308134104 | 0.353470543 | noDEG | noDEG |
| VvmiR399a | 9.82341 | 7.11555 | 8.01156 | -0.332858458 | -0.221786814 | 0.636946089 | 0.670316841 | 0.746070618 | 0.785337493 | noDEG | noDEG |
| VvmiR399c | 4.91171 | 0.001 | 3.00434 | NA | -0.636824641 | NA | NA | 0.540637747 | 0.593540247 | noDEG | noDEG |
| VvmiR399d | 4.91171 | 5.08254 | 3.00434 | 0.181714649 | -0.636824641 | 0.842117673 | 0.86843385 | 0.540637747 | 0.593540247 | noDEG | noDEG |
| VvmiR399d-1 | 7.85873 | 0.001 | 17.0246 | -3.841905748 | 1.187604255 | 0.007988211 | 0.011605515 | 0.047706823 | 0.059311186 | noDEG | noDEG |
| **VvmiR399e** | 63.8522 | 23.3797 | 41.0593 | -1.317091531 | -0.564674657 | 5.95E-05 | 0.00011266 | 0.047619766 | 0.059524708 | Down | noDEG |
| **VvmiR399g** | 33.3996 | 42.6933 | 104.15 | 0.486569046 | 1.713118004 | 0.142576054 | 0.171536815 | 1.12E-10 | 3.49E-10 | noDEG | Up |
| VvmiR399h | 9.82341 | 7.11555 | 6.00867 | -0.332858458 | -0.636824254 | 0.636946089 | 0.670316841 | 0.386882257 | 0.43619078 | noDEG | noDEG |
| **VvmiR399i** | 359.537 | 57.9409 | 338.489 | -2.501095223 | -0.014678855 | 3.81E-49 | 2.00E-48 | 0.893079444 | 0.925262487 | Down | noDEG |
| **VvmiR403a** | 436.16 | 832.519 | 599.866 | 1.065018196 | 0.532137327 | 1.23E-37 | 5.68E-37 | 3.63E-09 | 8.92E-09 | Up | noDEG |
| **VvmiR403b** | 436.16 | 837.602 | 603.871 | 1.073799193 | 0.541739374 | 2.42E-38 | 1.20E-37 | 1.78E-09 | 4.47E-09 | Up | noDEG |
| **VvmiR403c** | 436.16 | 832.519 | 599.866 | 1.065018196 | 0.532137327 | 1.23E-37 | 5.68E-37 | 3.63E-09 | 8.92E-09 | Up | noDEG |
| **VvmiR403d** | 436.16 | 837.602 | 603.871 | 1.073799193 | 0.541739374 | 2.42E-38 | 1.20E-37 | 1.78E-09 | 4.47E-09 | Up | noDEG |
| **VvmiR403e** | 436.16 | 832.519 | 598.864 | 1.065018196 | 0.529726917 | 1.23E-37 | 5.68E-37 | 4.33E-09 | 1.04E-08 | Up | noDEG |
| **VvmiR403f** | 437.142 | 836.585 | 600.867 | 1.068801603 | 0.531298434 | 5.07E-38 | 2.44E-37 | 3.71E-09 | 8.97E-09 | Up | noDEG |
| **VvmiR408** | 29.4702 | 35.5778 | 256.37 | 0.404107088 | 3.193250906 | 0.25946238 | 0.299679049 | 3.05E-48 | 1.60E-47 | noDEG | Up |
| **VvmiR447b** | 12.7704 | 6.09904 | 87.1257 | -0.933762819 | 2.842644776 | 0.173726471 | 0.207931683 | 8.41E-16 | 2.98E-15 | noDEG | Up |
| **VvmiR447b*** | 88.4107 | 45.7428 | 417.603 | -0.818285379 | 2.31219165 | 0.001374025 | 0.002299998 | 1.25E-55 | 6.99E-55 | noDEG | Up |
| **VvmiR477** | 311.402 | 178.905 | 1127.63 | -0.667193194 | 1.928793056 | 4.98E-07 | 1.22E-06 | 2.98E-117 | 1.80E-116 | noDEG | Up |
| VvmiR479 | 34123.6 | 36770.1 | 33541.4 | 0.24015468 | 0.047527644 | 1.22E-110 | 8.04E-110 | 1.57E-05 | 3.25E-05 | noDEG | noDEG |
| **VvmiR482** | 1369.38 | 2508.74 | 517.747 | 1.005826522 | -1.330853418 | 3.71E-100 | 2.38E-99 | 5.93E-80 | 3.41E-79 | Up | Down |
| **VvmiR535a** | 3366.48 | 6314.54 | 8758.64 | 1.039826112 | 1.45181827 | 3.02E-263 | 3.17E-262 | 0 | 0 | Up | Up |
| **VvmiR535b** | 3366.48 | 6314.54 | 8758.64 | 1.039826112 | 1.45181827 | 3.02E-263 | 3.17E-262 | 0 | 0 | Up | Up |
| **VvmiR535c** | 3366.48 | 6314.54 | 8758.64 | 1.039826112 | 1.45181827 | 3.02E-263 | 3.17E-262 | 0 | 0 | Up | Up |
| VvmiR828a | 8.84107 | 16.2641 | 13.0188 | 1.011789285 | 0.630656322 | 0.085767113 | 0.108263404 | 0.311247354 | 0.354390551 | noDEG | noDEG |
| VvmiR845d | 0.001 | 1.01651 | 0.001 | 0.181714512 | 0 | 0.929019692 | 0.947477037 | 0 | 0 | noDEG | ---- |
| VvmiR845e | 0.001 | 1.01651 | 0.001 | 0.181714512 | 0 | 0.929019692 | 0.947477037 | 0 | 0 | noDEG | ---- |

Notes: VvmiRNA with the absolute value of log2Fold (YB/SB) >=1 or the absolute value of log2Fold (MB/SB)>=1 could be the VvmiRNAs with significant difference during grape berry development, which were marked with bold font underlined.

**Table S4** Comparison of novel VvmiRNAs in grape stone hardening stage and other two stages

| miRNA ID | SB | YB | MB | log2Fold  (YB/SB) | log2Fold  (MB/SB) | pvalue  (YB/SB) | qvalue  (YB/SB) | pvalue  (MB/SB) | qvalue  (MB/SB) | Diff  (YB/SB) | Diff  (MB/SB) |
| --- | --- | --- | --- | --- | --- | --- | --- | --- | --- | --- | --- |
| **VvmiR1** | 14.73512 | 16.26411 | 0.001 | 0.27482369 | -4.808832734 | 0.59593014 | 0.631467258 | 0.000102864 | 0.000196338 | noDEG | Down |
| **VvmiR10** | 1421.448 | 0.001 | 0.001 | -11.34075499 | -11.40079144 | 2.48E-181 | 2.12E-180 | 4.22E-183 | 2.85E-182 | Down | Down |
| **VvmiR11** | 11.7881 | 32.52823 | 0.001 | 1.596751739 | -4.486905128 | 0.000530213 | 0.000942148 | 0.000610019 | 0.001043156 | Up | Down |
| **VvmiR12** | 46.17004 | 0.001 | 0.001 | -6.396494455 | -6.456530906 | 6.17E-12 | 1.90E-11 | 3.99E-12 | 1.27E-11 | Down | Down |
| **VvmiR13** | 13505.23 | 7334.099 | 29283.26 | -0.748435942 | 1.188912134 | 7.40E-295 | 8.99E-294 | 0 | 0 | noDEG | Up |
| VvmiR13* | 1308.479 | 991.0944 | 1079.558 | -0.268405765 | -0.205095845 | 8.88E-06 | 1.83E-05 | 0.000526265 | 0.000910082 | noDEG | noDEG |
| **VvmiR14** | 22.59385 | 0.001 | 0.001 | -5.3654676 | -5.425504052 | 1.41E-06 | 3.14E-06 | 1.08E-06 | 2.48E-06 | Down | Down |
| **VvmiR15** | 12.77044 | 0.001 | 0.001 | -4.542345706 | -4.602382158 | 0.000398768 | 0.000728186 | 0.000335307 | 0.000590963 | Down | Down |
| VvmiR15* | 2.947024 | 0.001 | 0.001 | NA | NA | NA | NA | NA | NA | noDEG | noDEG |
| VvmiR16 | 6.876389 | 0.001 | 10.01445 | -3.649260539 | 0.614714294 | 0.014816323 | 0.020680185 | 0.385361487 | 0.436616463 | noDEG | noDEG |
| **VvmiR17** | 75.64028 | 0.001 | 0.001 | -7.108692176 | -7.168728628 | 5.39E-18 | 2.01E-17 | 2.92E-18 | 1.08E-17 | Down | Down |
| VvmiR18 | 4.911707 | 0.001 | 0.001 | NA | NA | NA | NA | NA | NA | noDEG | noDEG |
| **VvmiR19** | 40.27599 | 191.1033 | 65.09393 | 2.378751357 | 0.764957138 | 8.62E-28 | 3.76E-27 | 0.00729018 | 0.010817687 | Up | noDEG |
| **VvmiR2** | 56.9758 | 0.001 | 0.001 | -6.699886749 | -6.759923201 | 3.19E-14 | 1.07E-13 | 1.92E-14 | 6.50E-14 | Down | Down |
| **VvmiR20** | 10.80575 | 0.001 | 0.001 | -4.301336682 | -4.361373134 | 0.001300485 | 0.002233546 | 0.001115868 | 0.001853066 | Down | Down |
| **VvmiR21** | 9.823413 | 12.19809 | 0.001 | 0.444749628 | -4.223870184 | 0.470542799 | 0.525098486 | 0.002052965 | 0.003256428 | noDEG | Down |
| VvmiR22 | 4.911707 | 0.001 | 0.001 | NA | NA | NA | NA | NA | NA | noDEG | noDEG |
| **VvmiR23** | 18.66449 | 0.001 | 0.001 | -5.089833561 | -5.149870012 | 1.29E-05 | 2.54E-05 | 1.02E-05 | 2.18E-05 | Down | Down |
| **VvmiR24** | 91.35774 | 0.001 | 184.2659 | -7.381064435 | 1.084544416 | 4.96E-21 | 1.94E-20 | 1.40E-09 | 3.61E-09 | Down | Up |
| **VvmiR24*** | 1.964683 | 0.001 | 12.01734 | NA | 2.685103307 | NA | NA | 0.003714375 | 0.005772339 | noDEG | Up |
| VvmiR25 | 16.6998 | 10.16507 | 20.0289 | -0.583820053 | 0.334606535 | 0.301409807 | 0.345536801 | 0.483011822 | 0.539285044 | noDEG | noDEG |
| VvmiR26 | 8.841072 | 6.099043 | 6.008671 | -0.403247801 | -0.484821209 | 0.592053586 | 0.630250592 | 0.521116102 | 0.576234152 | noDEG | noDEG |
| **VvmiR27** | 21.61151 | 10.16507 | 0.001 | -0.955789105 | -5.361373801 | 0.071156529 | 0.091317546 | 1.89E-06 | 4.22E-06 | noDEG | Down |
| VvmiR27* | 0.001 | 2.033014 | 13.01879 | 1.181714512 | -0.286881287 | 0.490890014 | 0.542562647 | 0.589189213 | 0.642244166 | noDEG | noDEG |
| **VvmiR28** | 16.6998 | 0.001 | 50378.7 | -4.929368298 | 2.721757618 | 3.97E-05 | 7.58E-05 | 0 | 0 | Down | Up |
| **VvmiR29** | 8029.658 | 3214.195 | 1.001445 | -1.188490462 | 0.1001411 | 0 | 0 | 0.961007539 | 0.96943743 | Down | noDEG |
| VvmiR3 | 5.894048 | 0.001 | 0.001 | -3.426868187 | -3.486904639 | 0.027677797 | 0.036957059 | 0.02511877 | 0.032732675 | noDEG | noDEG |
| **VvmiR30** | 10018.9 | 6090.911 | 22848.97 | -0.585603569 | 1.261759183 | 1.24E-142 | 9.92E-142 | 0 | 0 | noDEG | Up |
| VvmiR31 | 6.876389 | 0.001 | 0.001 | -3.649260539 | -3.70929699 | 0.014816323 | 0.020680185 | 0.01328583 | 0.018243229 | noDEG | noDEG |
| VvmiR32 | 27.50556 | 14.2311 | 43.06214 | -0.818285474 | 0.719050912 | 0.074279774 | 0.094799049 | 0.038653341 | 0.048847629 | noDEG | noDEG |
| VvmiR33 | 7.858731 | 0.001 | 0.001 | -3.841905748 | -3.901942199 | 0.007988211 | 0.011605515 | 0.007080699 | 0.010644188 | noDEG | noDEG |
| **VvmiR34** | 1586.481 | 0.001 | 12498.04 | -11.49922391 | 3.050153885 | 8.80E-197 | 7.81E-196 | 0 | 0 | Down | Up |
| VvmiR35 | 9.823413 | 0.001 | 0.001 | -4.163833733 | -4.223870184 | 0.002367592 | 0.003865115 | 0.002052965 | 0.003256428 | Down | Down |
| **VvmiR36** | 10.80575 | 0.001 | 0.001 | -4.301336682 | -4.361373134 | 0.001300485 | 0.002233546 | 0.001115868 | 0.001853066 | Down | Down |
| **VvmiR37** | 37314.24 | 51395.62 | 0.001 | 0.594309736 | -16.11508451 | 0 | 0 | 0 | 0 | noDEG | Down |
| **VvmiR37*** | 2542.299 | 3505.933 | 0.001 | 0.596055332 | -12.23956383 | 1.70E-57 | 1.01E-56 | 4.36E-281 | 3.58E-280 | noDEG | Down |
| **VvmiR38** | 617.8927 | 318.1667 | 4340.263 | -0.825182859 | 2.884708403 | 1.57E-17 | 5.77E-17 | 0 | 0 | noDEG | Up |
| **VvmiR38*** | 16.6998 | 18.29713 | 139.2009 | 0.264177169 | 3.13161998 | 0.588067531 | 0.628905554 | 1.57E-26 | 6.70E-26 | noDEG | Up |
| **VvmiR39** | 9.823413 | 24.39617 | 0.001 | 1.444749036 | -4.223870184 | 0.005310165 | 0.007813045 | 0.002052965 | 0.003256428 | Up | Down |
| VvmiR4 | 4.911707 | 10.16507 | 7.010116 | 1.181714366 | 0.585567987 | 0.123459095 | 0.150894449 | 0.486843422 | 0.540937135 | noDEG | noDEG |
| **VvmiR40** | 14.73512 | 5.082536 | 0.001 | -1.403247753 | -4.808832734 | 0.042935533 | 0.056674903 | 0.000102864 | 0.000196338 | noDEG | Down |
| **VvmiR41** | 8.841072 | 15.24761 | 0.001 | 0.918680531 | -4.07186714 | 0.124763914 | 0.151686653 | 0.003800233 | 0.005846512 | noDEG | Down |
| **VvmiR42** | 9.823413 | 0.001 | 0.001 | -4.163833733 | -4.223870184 | 0.002367592 | 0.003865115 | 0.002052965 | 0.003256428 | Down | Down |
| **VvmiR43** | 2743.679 | 0.001 | 0.001 | -12.28950536 | -12.34954182 | 1.65E-294 | 1.91E-293 | 3.74E-297 | 3.18E-296 | Down | Down |
| **VvmiR43*** | 12.77044 | 0.001 | 0.001 | -4.542345706 | -4.602382158 | 0.000398768 | 0.000728186 | 0.000335307 | 0.000590963 | Down | Down |
| **VvmiR44** | 59.92282 | 44.72631 | 20.0289 | -0.289591159 | -1.508668159 | 0.306872886 | 0.34920018 | 1.34E-05 | 2.83E-05 | noDEG | Down |
| **VvmiR44*** | 37.32897 | 24.39617 | 11.0159 | -0.481250405 | -1.688354163 | 0.194821263 | 0.231977896 | 0.000193763 | 0.0003594 | noDEG | Down |
| **VvmiR45** | 59.92282 | 0.001 | 0.001 | -6.772642992 | -6.832679444 | 7.83E-15 | 2.66E-14 | 4.64E-15 | 1.62E-14 | Down | Down |
| **VvmiR46** | 21.61151 | 15.24761 | 9.013006 | -0.370826131 | -1.18936545 | 0.438464988 | 0.491676758 | 0.02939624 | 0.037561862 | noDEG | Down |
| VvmiR47 | 9.823413 | 0.001 | 8.011561 | -4.163833733 | -0.221786814 | 0.002367592 | 0.003865115 | 0.746070618 | 0.785337493 | noDEG | noDEG |
| VvmiR48 | 5.894048 | 10.16507 | 0.001 | 0.918680058 | -3.486904639 | 0.210065055 | 0.248846296 | 0.02511877 | 0.032732675 | noDEG | noDEG |
| **VvmiR49** | 14.73512 | 77.25454 | 5.007226 | 2.522751531 | -1.484821161 | 6.67E-13 | 2.17E-12 | 0.032904349 | 0.041812156 | Up | noDEG |
| VvmiR5 | 4.911707 | 0.001 | 0.001 | NA | NA | NA | NA | NA | NA | noDEG | noDEG |
| VvmiR50 | 4.911707 | 0.001 | 0.001 | NA | NA | NA | NA | NA | NA | noDEG | noDEG |
| **VvmiR51** | 11.7881 | 0.001 | 0.001 | -4.426868677 | -4.486905128 | 0.000718262 | 0.001266554 | 0.000610019 | 0.001043156 | Down | Down |
| **VvmiR52** | 44.20536 | 95.55167 | 68.09827 | 1.2444504 | 0.695751008 | 8.66E-07 | 2.02E-06 | 0.011525989 | 0.016364058 | Up | noDEG |
| **VvmiR53** | 22.59385 | 0.001 | 0.001 | -5.3654676 | -5.425504052 | 1.41E-06 | 3.14E-06 | 1.08E-06 | 2.48E-06 | Down | Down |
| VvmiR53* | 2.947024 | 0.001 | 0.001 | NA | NA | NA | NA | NA | NA | noDEG | noDEG |
| **VvmiR54** | 13.75278 | 0.001 | 5.007226 | -4.649260749 | -1.385285627 | 0.000222486 | 0.00041447 | 0.050440249 | 0.062372351 | Down | noDEG |
| **VvmiR55** | 85.4637 | 263.2753 | 20.0289 | 1.755579117 | -2.020874417 | 5.39E-26 | 2.26E-25 | 1.86E-10 | 5.72E-10 | Up | Down |
| VvmiR56 | 7.858731 | 8.132057 | 0.001 | 0.18171458 | -3.901942199 | 0.801078193 | 0.831681181 | 0.007080699 | 0.010644188 | noDEG | noDEG |
| **VvmiR57** | 23.57619 | 0.001 | 0.001 | -5.426868065 | -5.486904516 | 8.17E-07 | 1.99E-06 | 6.23E-07 | 1.45E-06 | Down | Down |
| **VvmiR58** | 10.80575 | 0.001 | 0.001 | -4.301336682 | -4.361373134 | 0.001300485 | 0.002233546 | 0.001115868 | 0.001853066 | Down | Down |
| **VvmiR59** | 10.80575 | 0.001 | 0.001 | -4.301336682 | -4.361373134 | 0.001300485 | 0.002233546 | 0.001115868 | 0.001853066 | Down | Down |
| VvmiR6 | 19.64683 | 25.41268 | 17.02457 | 0.503642597 | -0.134324023 | 0.243016627 | 0.283519399 | 0.778253355 | 0.813628508 | noDEG | noDEG |
| **VvmiR60** | 9.823413 | 52.85837 | 9.013006 | 2.560226299 | -0.051861833 | 2.06E-09 | 5.94E-09 | 0.937840062 | 0.958680952 | Up | noDEG |
| **VvmiR61** | 156.1923 | 0.001 | 183.2645 | -8.154788901 | 0.302958188 | 1.37E-32 | 6.07E-32 | 0.05338394 | 0.065310139 | Down | noDEG |
| **VvmiR62** | 8.841072 | 0.001 | 0.001 | -4.011830688 | -4.07186714 | 0.004335454 | 0.006419808 | 0.003800233 | 0.005846512 | Down | Down |
| **VvmiR63** | 21.61151 | 0.001 | 11.0159 | -5.30133735 | -0.899858338 | 2.44E-06 | 5.31E-06 | 0.083143828 | 0.100647792 | Down | noDEG |
| **VvmiR64** | 24.55853 | 22.36316 | 10.01445 | -0.002709525 | -1.221786848 | 0.994867778 | 0.994867778 | 0.017700355 | 0.023947539 | noDEG | Down |
| **VvmiR65** | 44.20536 | 95.55167 | 68.09827 | 1.2444504 | 0.695751008 | 8.66E-07 | 2.02E-06 | 0.011525989 | 0.016364058 | Up | noDEG |
| **VvmiR66** | 44.20536 | 95.55167 | 68.09827 | 1.2444504 | 0.695751008 | 8.66E-07 | 2.02E-06 | 0.011525989 | 0.016364058 | Up | noDEG |
| VvmiR67 | 7.858731 | 0.001 | 9.013006 | -3.841905748 | 0.270066152 | 0.007988211 | 0.011605515 | 0.700929941 | 0.746360585 | noDEG | noDEG |
| **VvmiR68** | 44.20536 | 95.55167 | 68.09827 | 1.2444504 | 0.695751008 | 8.66E-07 | 2.02E-06 | 0.011525989 | 0.016364058 | Up | noDEG |
| **VvmiR69** | 44.20536 | 95.55167 | 68.09827 | 1.2444504 | 0.695751008 | 8.66E-07 | 2.02E-06 | 0.011525989 | 0.016364058 | Up | noDEG |
| VvmiR7 | 9.823413 | 0.001 | 0.001 | -4.163833733 | -4.223870184 | 0.002367592 | 0.003865115 | 0.002052965 | 0.003256428 | noDEG | noDEG |
| **VvmiR70** | 44.20536 | 95.55167 | 68.09827 | 1.2444504 | 0.695751008 | 8.66E-07 | 2.02E-06 | 0.011525989 | 0.016364058 | Up | noDEG |
| **VvmiR71** | 8029.658 | 3214.195 | 50378.7 | -1.188490462 | 2.721757618 | 0 | 0 | 0 | 0 | Down | Up |
| **VvmiR72** | 44.20536 | 95.55167 | 68.09827 | 1.2444504 | 0.695751008 | 8.66E-07 | 2.02E-06 | 0.011525989 | 0.016364058 | Up | noDEG |
| VvmiR8 | 7.858731 | 8.132057 | 0.001 | 0.18171458 | -3.901942199 | 0.801078193 | 0.831681181 | 0.007080699 | 0.010644188 | noDEG | noDEG |
| VvmiR9 | 6.876389 | 10.16507 | 0.001 | 0.696287706 | -3.70929699 | 0.323498827 | 0.366314849 | 0.01328583 | 0.018243229 | noDEG | noDEG |

Notes: VvmiRNA with the absolute value of log2Fold (YB/SB)>=1 or the absolute value of log2Fold (MB/SB)>=1 could be the VvmiRNAs with significant difference during grape berry development, which were marked with bold font underlined.

**Table S5** List of various SNP Edit types of VvmiRNAs

| Mir_name | Edit_type | PosINseed | Snp_count | Total | Rate |
| --- | --- | --- | --- | --- | --- |
| VvmiR156a | G->A | 14 | 28 | 10912 | 0.26% |
| VvmiR156a | G->C | 14 | 13 | 10912 | 0.12% |
| VvmiR156a | G->T | 11 | 35 | 10912 | 0.32% |
| ***VvmiR156a*** | ***G->T*** | ***14*** | ***10771*** | ***10912*** | ***98.71%*** |
| VvmiR156b | A->G | 16 | 15 | 11567 | 0.13% |
| VvmiR156b | A->G | 5 | 6 | 11567 | 0.05% |
| VvmiR156b | A->G | 7 | 7 | 11567 | 0.06% |
| VvmiR156b | A->G | 8 | 27 | 11567 | 0.23% |
| VvmiR156b | G->A | 15 | 8 | 11567 | 0.07% |
| VvmiR156b | G->A | 6 | 5 | 11567 | 0.04% |
| VvmiR156b | G->T | 13 | 11 | 11567 | 0.10% |
| VvmiR156b | T->A | 14 | 16 | 11567 | 0.14% |
| VvmiR156b | T->C | 14 | 13 | 11567 | 0.11% |
| VvmiR156b | T->G | 14 | 42 | 11567 | 0.36% |
| VvmiR156c | A->G | 16 | 15 | 10898 | 0.14% |
| VvmiR156c | A->G | 5 | 6 | 10898 | 0.06% |
| VvmiR156c | A->G | 7 | 7 | 10898 | 0.06% |
| VvmiR156c | A->G | 8 | 27 | 10898 | 0.25% |
| VvmiR156c | G->A | 15 | 7 | 10898 | 0.06% |
| VvmiR156c | G->A | 6 | 5 | 10898 | 0.05% |
| VvmiR156c | G->T | 13 | 11 | 10898 | 0.10% |
| VvmiR156c | T->A | 14 | 15 | 10898 | 0.14% |
| VvmiR156c | T->C | 14 | 13 | 10898 | 0.12% |
| VvmiR156c | T->G | 14 | 39 | 10898 | 0.36% |
| VvmiR156d | A->G | 12 | 5 | 11727 | 0.04% |
| VvmiR156d | A->G | 16 | 15 | 11727 | 0.13% |
| VvmiR156d | A->G | 5 | 6 | 11727 | 0.05% |
| VvmiR156d | A->G | 7 | 7 | 11727 | 0.06% |
| VvmiR156d | A->G | 8 | 27 | 11727 | 0.23% |
| VvmiR156d | G->A | 15 | 8 | 11727 | 0.07% |
| VvmiR156d | G->A | 6 | 5 | 11727 | 0.04% |
| VvmiR156d | G->T | 13 | 11 | 11727 | 0.09% |
| VvmiR156d | T->A | 14 | 17 | 11727 | 0.15% |
| VvmiR156d | T->C | 14 | 13 | 11727 | 0.11% |
| VvmiR156d | T->G | 14 | 43 | 11727 | 0.37% |
| ***VvmiR156e*** | ***G->A*** | ***8*** | ***9056*** | ***9081*** | ***99.72%*** |
| VvmiR156f | A->C | 13 | 5 | 30349 | 0.02% |
| VvmiR156f | A->C | 8 | 8 | 30349 | 0.03% |
| VvmiR156f | A->G | 13 | 12 | 30349 | 0.04% |
| VvmiR156f | A->G | 15 | 36 | 30349 | 0.12% |
| VvmiR156f | A->G | 17 | 23 | 30349 | 0.08% |
| VvmiR156f | A->G | 6 | 31 | 30349 | 0.10% |
| VvmiR156f | A->G | 8 | 10 | 30349 | 0.03% |
| VvmiR156f | A->G | 9 | 14 | 30349 | 0.05% |
| VvmiR156f | A->T | 13 | 5 | 30349 | 0.02% |
| VvmiR156f | A->T | 15 | 13 | 30349 | 0.04% |
| VvmiR156f | A->T | 6 | 9 | 30349 | 0.03% |
| VvmiR156f | C->A | 5 | 13 | 30349 | 0.04% |
| VvmiR156f | C->G | 5 | 8 | 30349 | 0.03% |
| VvmiR156f | C->T | 5 | 18 | 30349 | 0.06% |
| VvmiR156f | G->A | 10 | 6 | 30349 | 0.02% |
| VvmiR156f | G->A | 14 | 16 | 30349 | 0.05% |
| VvmiR156f | G->A | 16 | 25 | 30349 | 0.08% |
| VvmiR156f | G->A | 7 | 6 | 30349 | 0.02% |
| VvmiR156f | G->C | 14 | 10 | 30349 | 0.03% |
| VvmiR156f | G->C | 16 | 6 | 30349 | 0.02% |
| VvmiR156f | G->T | 10 | 8 | 30349 | 0.03% |
| VvmiR156f | G->T | 14 | 11 | 30349 | 0.04% |
| VvmiR156f | G->T | 16 | 21 | 30349 | 0.07% |
| VvmiR156f | G->T | 7 | 16 | 30349 | 0.05% |
| VvmiR156f | T->A | 12 | 6 | 30349 | 0.02% |
| VvmiR156f | T->C | 12 | 10 | 30349 | 0.03% |
| VvmiR156f | T->G | 12 | 13 | 30349 | 0.04% |
| VvmiR156g | A->C | 13 | 5 | 30311 | 0.02% |
| VvmiR156g | A->C | 8 | 8 | 30311 | 0.03% |
| VvmiR156g | A->G | 13 | 12 | 30311 | 0.04% |
| VvmiR156g | A->G | 15 | 36 | 30311 | 0.12% |
| VvmiR156g | A->G | 17 | 23 | 30311 | 0.08% |
| VvmiR156g | A->G | 6 | 31 | 30311 | 0.10% |
| VvmiR156g | A->G | 8 | 10 | 30311 | 0.03% |
| VvmiR156g | A->G | 9 | 14 | 30311 | 0.05% |
| VvmiR156g | A->T | 13 | 5 | 30311 | 0.02% |
| VvmiR156g | A->T | 15 | 13 | 30311 | 0.04% |
| VvmiR156g | A->T | 6 | 9 | 30311 | 0.03% |
| VvmiR156g | C->A | 5 | 13 | 30311 | 0.04% |
| VvmiR156g | C->G | 5 | 8 | 30311 | 0.03% |
| VvmiR156g | C->T | 5 | 18 | 30311 | 0.06% |
| VvmiR156g | G->A | 10 | 6 | 30311 | 0.02% |
| VvmiR156g | G->A | 14 | 16 | 30311 | 0.05% |
| VvmiR156g | G->A | 16 | 25 | 30311 | 0.08% |
| VvmiR156g | G->A | 7 | 6 | 30311 | 0.02% |
| VvmiR156g | G->C | 14 | 10 | 30311 | 0.03% |
| VvmiR156g | G->C | 16 | 6 | 30311 | 0.02% |
| VvmiR156g | G->T | 10 | 8 | 30311 | 0.03% |
| VvmiR156g | G->T | 14 | 11 | 30311 | 0.04% |
| VvmiR156g | G->T | 16 | 21 | 30311 | 0.07% |
| VvmiR156g | G->T | 7 | 16 | 30311 | 0.05% |
| VvmiR156g | T->A | 12 | 6 | 30311 | 0.02% |
| VvmiR156g | T->C | 12 | 10 | 30311 | 0.03% |
| VvmiR156g | T->G | 12 | 13 | 30311 | 0.04% |
| ***VvmiR156h*** | ***A->T*** | ***14*** | ***32*** | ***33*** | ***96.97%*** |
| VvmiR156i | A->C | 13 | 5 | 30311 | 0.02% |
| VvmiR156i | A->C | 8 | 8 | 30311 | 0.03% |
| VvmiR156i | A->G | 13 | 12 | 30311 | 0.04% |
| VvmiR156i | A->G | 15 | 36 | 30311 | 0.12% |
| VvmiR156i | A->G | 17 | 23 | 30311 | 0.08% |
| VvmiR156i | A->G | 6 | 31 | 30311 | 0.10% |
| VvmiR156i | A->G | 8 | 10 | 30311 | 0.03% |
| VvmiR156i | A->G | 9 | 14 | 30311 | 0.05% |
| VvmiR156i | A->T | 13 | 5 | 30311 | 0.02% |
| VvmiR156i | A->T | 15 | 13 | 30311 | 0.04% |
| VvmiR156i | A->T | 6 | 9 | 30311 | 0.03% |
| VvmiR156i | C->A | 5 | 13 | 30311 | 0.04% |
| VvmiR156i | C->G | 5 | 8 | 30311 | 0.03% |
| VvmiR156i | C->T | 5 | 18 | 30311 | 0.06% |
| VvmiR156i | G->A | 10 | 6 | 30311 | 0.02% |
| VvmiR156i | G->A | 14 | 16 | 30311 | 0.05% |
| VvmiR156i | G->A | 16 | 25 | 30311 | 0.08% |
| VvmiR156i | G->A | 7 | 6 | 30311 | 0.02% |
| VvmiR156i | G->C | 14 | 10 | 30311 | 0.03% |
| VvmiR156i | G->C | 16 | 6 | 30311 | 0.02% |
| VvmiR156i | G->T | 10 | 8 | 30311 | 0.03% |
| VvmiR156i | G->T | 14 | 11 | 30311 | 0.04% |
| VvmiR156i | G->T | 16 | 21 | 30311 | 0.07% |
| VvmiR156i | G->T | 7 | 16 | 30311 | 0.05% |
| VvmiR156i | T->A | 12 | 6 | 30311 | 0.02% |
| VvmiR156i | T->C | 12 | 10 | 30311 | 0.03% |
| VvmiR156i | T->G | 12 | 13 | 30311 | 0.04% |
| VvmiR159c | C->T | 17 | 18 | 1362 | 1.32% |
| VvmiR159c | T->C | 7 | 9 | 1362 | 0.66% |
| ***VvmiR160a*** | ***A->T*** | ***15*** | ***20*** | ***27*** | ***74.07%*** |
| ***VvmiR160b*** | ***A->T*** | ***15*** | ***20*** | ***27*** | ***74.07%*** |
| VvmiR160c | T->A | 15 | 7 | 24 | 29.17% |
| VvmiR160d | T->A | 15 | 7 | 28 | 25.00% |
| VvmiR160e | T->A | 15 | 7 | 28 | 25.00% |
| VvmiR162 | A->G | 16 | 6 | 4119 | 0.15% |
| VvmiR162 | A->G | 6 | 6 | 4119 | 0.15% |
| VvmiR162 | C->T | 9 | 7 | 4119 | 0.17% |
| VvmiR164a | A->G | 6 | 6 | 3849 | 0.16% |
| VvmiR164a | C->T | 16 | 8 | 3849 | 0.21% |
| VvmiR164a | C->T | 9 | 29 | 3849 | 0.75% |
| VvmiR164a | G->T | 17 | 9 | 3849 | 0.23% |
| VvmiR164a | G->T | 8 | 7 | 3849 | 0.18% |
| ***VvmiR164b*** | ***A->G*** | ***17*** | ***74*** | ***74*** | ***100.00%*** |
| VvmiR164c | A->G | 6 | 6 | 3874 | 0.15% |
| VvmiR164c | C->A | 14 | 5 | 3874 | 0.13% |
| VvmiR164c | C->T | 16 | 8 | 3874 | 0.21% |
| VvmiR164c | C->T | 9 | 30 | 3874 | 0.77% |
| VvmiR164c | G->T | 17 | 9 | 3874 | 0.23% |
| VvmiR164c | G->T | 8 | 7 | 3874 | 0.18% |
| VvmiR164d | A->G | 6 | 6 | 3850 | 0.16% |
| VvmiR164d | C->T | 16 | 8 | 3850 | 0.21% |
| VvmiR164d | C->T | 9 | 29 | 3850 | 0.75% |
| VvmiR164d | G->T | 17 | 9 | 3850 | 0.23% |
| VvmiR164d | G->T | 8 | 7 | 3850 | 0.18% |
| VvmiR166a | A->C | 15 | 9 | 17726 | 0.05% |
| VvmiR166a | A->C | 5 | 5 | 17726 | 0.03% |
| VvmiR166a | A->C | 8 | 6 | 17726 | 0.03% |
| VvmiR166a | A->G | 15 | 7 | 17726 | 0.04% |
| VvmiR166a | A->G | 5 | 13 | 17726 | 0.07% |
| VvmiR166a | A->G | 8 | 7 | 17726 | 0.04% |
| VvmiR166a | C->A | 11 | 7 | 17726 | 0.04% |
| VvmiR166a | C->A | 14 | 33 | 17726 | 0.19% |
| VvmiR166a | C->A | 6 | 7 | 17726 | 0.04% |
| VvmiR166a | C->G | 11 | 14 | 17726 | 0.08% |
| VvmiR166a | C->G | 14 | 32 | 17726 | 0.18% |
| VvmiR166a | C->T | 11 | 5 | 17726 | 0.03% |
| VvmiR166a | C->T | 14 | 22 | 17726 | 0.12% |
| VvmiR166a | C->T | 6 | 10 | 17726 | 0.06% |
| VvmiR166a | C->T | 7 | 9 | 17726 | 0.05% |
| VvmiR166a | G->A | 3 | 8 | 17726 | 0.05% |
| VvmiR166a | G->A | 4 | 7 | 17726 | 0.04% |
| VvmiR166a | G->T | 10 | 6 | 17726 | 0.03% |
| VvmiR166a | G->T | 3 | 10 | 17726 | 0.06% |
| VvmiR166a | G->T | 4 | 13 | 17726 | 0.07% |
| VvmiR166a | T->A | 12 | 7 | 17726 | 0.04% |
| VvmiR166a | T->C | 12 | 15 | 17726 | 0.08% |
| VvmiR166a | T->C | 13 | 6 | 17726 | 0.03% |
| VvmiR166a | T->G | 13 | 12 | 17726 | 0.07% |
| VvmiR166b | A->C | 15 | 148 | 103248 | 0.14% |
| VvmiR166b | A->C | 5 | 21 | 103248 | 0.02% |
| VvmiR166b | A->C | 8 | 30 | 103248 | 0.03% |
| VvmiR166b | A->G | 15 | 126 | 103248 | 0.12% |
| VvmiR166b | A->G | 5 | 65 | 103248 | 0.06% |
| VvmiR166b | A->G | 8 | 32 | 103248 | 0.03% |
| VvmiR166b | A->T | 15 | 51 | 103248 | 0.05% |
| VvmiR166b | A->T | 5 | 15 | 103248 | 0.01% |
| VvmiR166b | A->T | 8 | 17 | 103248 | 0.02% |
| VvmiR166b | C->A | 14 | 133 | 103248 | 0.13% |
| VvmiR166b | C->A | 6 | 48 | 103248 | 0.05% |
| VvmiR166b | C->A | 7 | 15 | 103248 | 0.01% |
| VvmiR166b | C->G | 14 | 54 | 103248 | 0.05% |
| VvmiR166b | C->G | 6 | 91 | 103248 | 0.09% |
| VvmiR166b | C->G | 7 | 44 | 103248 | 0.04% |
| VvmiR166b | C->T | 14 | 87 | 103248 | 0.08% |
| VvmiR166b | C->T | 6 | 61 | 103248 | 0.06% |
| VvmiR166b | C->T | 7 | 77 | 103248 | 0.07% |
| VvmiR166b | G->A | 10 | 19 | 103248 | 0.02% |
| VvmiR166b | G->A | 9 | 15 | 103248 | 0.01% |
| VvmiR166b | G->C | 10 | 8 | 103248 | 0.01% |
| VvmiR166b | G->C | 9 | 7 | 103248 | 0.01% |
| VvmiR166b | G->T | 10 | 12 | 103248 | 0.01% |
| VvmiR166b | G->T | 9 | 29 | 103248 | 0.03% |
| VvmiR166b | T->A | 12 | 8 | 103248 | 0.01% |
| VvmiR166b | T->A | 13 | 21 | 103248 | 0.02% |
| VvmiR166b | T->A | 16 | 109 | 103248 | 0.11% |
| VvmiR166b | T->A | 17 | 7 | 103248 | 0.01% |
| VvmiR166b | T->C | 12 | 13 | 103248 | 0.01% |
| VvmiR166b | T->C | 13 | 26 | 103248 | 0.03% |
| VvmiR166b | T->C | 16 | 37 | 103248 | 0.04% |
| VvmiR166b | T->C | 17 | 33 | 103248 | 0.03% |
| VvmiR166b | T->G | 12 | 19 | 103248 | 0.02% |
| VvmiR166b | T->G | 13 | 44 | 103248 | 0.04% |
| VvmiR166b | T->G | 16 | 161 | 103248 | 0.16% |
| VvmiR166c | A->C | 15 | 858 | 293162 | 0.29% |
| VvmiR166c | A->C | 5 | 78 | 293162 | 0.03% |
| VvmiR166c | A->C | 8 | 102 | 293162 | 0.03% |
| VvmiR166c | A->G | 15 | 484 | 293162 | 0.17% |
| VvmiR166c | A->G | 5 | 178 | 293162 | 0.06% |
| VvmiR166c | A->G | 8 | 107 | 293162 | 0.04% |
| VvmiR166c | A->T | 15 | 240 | 293162 | 0.08% |
| VvmiR166c | A->T | 5 | 42 | 293162 | 0.01% |
| VvmiR166c | A->T | 8 | 79 | 293162 | 0.03% |
| VvmiR166c | C->A | 14 | 481 | 293162 | 0.16% |
| VvmiR166c | C->A | 6 | 158 | 293162 | 0.05% |
| VvmiR166c | C->A | 7 | 43 | 293162 | 0.01% |
| VvmiR166c | C->G | 14 | 201 | 293162 | 0.07% |
| VvmiR166c | C->G | 6 | 289 | 293162 | 0.10% |
| VvmiR166c | C->G | 7 | 97 | 293162 | 0.03% |
| VvmiR166c | C->T | 14 | 423 | 293162 | 0.14% |
| VvmiR166c | C->T | 6 | 262 | 293162 | 0.09% |
| VvmiR166c | C->T | 7 | 212 | 293162 | 0.07% |
| VvmiR166c | G->A | 10 | 41 | 293162 | 0.01% |
| VvmiR166c | G->A | 9 | 30 | 293162 | 0.01% |
| VvmiR166c | G->C | 10 | 34 | 293162 | 0.01% |
| VvmiR166c | G->C | 9 | 14 | 293162 | 0.00% |
| VvmiR166c | G->T | 10 | 50 | 293162 | 0.02% |
| VvmiR166c | G->T | 9 | 113 | 293162 | 0.04% |
| VvmiR166c | T->A | 12 | 17 | 293162 | 0.01% |
| VvmiR166c | T->A | 13 | 58 | 293162 | 0.02% |
| VvmiR166c | T->A | 16 | 295 | 293162 | 0.10% |
| VvmiR166c | T->A | 17 | 20 | 293162 | 0.01% |
| VvmiR166c | T->C | 12 | 80 | 293162 | 0.03% |
| VvmiR166c | T->C | 13 | 63 | 293162 | 0.02% |
| VvmiR166c | T->C | 16 | 108 | 293162 | 0.04% |
| VvmiR166c | T->C | 17 | 221 | 293162 | 0.08% |
| VvmiR166c | T->G | 12 | 37 | 293162 | 0.01% |
| VvmiR166c | T->G | 13 | 114 | 293162 | 0.04% |
| VvmiR166c | T->G | 16 | 401 | 293162 | 0.14% |
| VvmiR166c | T->G | 17 | 6 | 293162 | 0.00% |
| VvmiR166d | A->C | 15 | 858 | 293357 | 0.29% |
| VvmiR166d | A->C | 5 | 78 | 293357 | 0.03% |
| VvmiR166d | A->C | 8 | 102 | 293357 | 0.03% |
| VvmiR166d | A->G | 15 | 484 | 293357 | 0.17% |
| VvmiR166d | A->G | 5 | 178 | 293357 | 0.06% |
| VvmiR166d | A->G | 8 | 107 | 293357 | 0.04% |
| VvmiR166d | A->T | 15 | 240 | 293357 | 0.08% |
| VvmiR166d | A->T | 5 | 42 | 293357 | 0.01% |
| VvmiR166d | A->T | 8 | 79 | 293357 | 0.03% |
| VvmiR166d | C->A | 14 | 480 | 293357 | 0.16% |
| VvmiR166d | C->A | 6 | 158 | 293357 | 0.05% |
| VvmiR166d | C->A | 7 | 44 | 293357 | 0.02% |
| VvmiR166d | C->G | 14 | 202 | 293357 | 0.07% |
| VvmiR166d | C->G | 6 | 289 | 293357 | 0.10% |
| VvmiR166d | C->G | 7 | 97 | 293357 | 0.03% |
| VvmiR166d | C->T | 14 | 424 | 293357 | 0.14% |
| VvmiR166d | C->T | 6 | 262 | 293357 | 0.09% |
| VvmiR166d | C->T | 7 | 212 | 293357 | 0.07% |
| VvmiR166d | G->A | 10 | 41 | 293357 | 0.01% |
| VvmiR166d | G->A | 9 | 30 | 293357 | 0.01% |
| VvmiR166d | G->C | 10 | 34 | 293357 | 0.01% |
| VvmiR166d | G->C | 9 | 14 | 293357 | 0.00% |
| VvmiR166d | G->T | 10 | 50 | 293357 | 0.02% |
| VvmiR166d | G->T | 9 | 113 | 293357 | 0.04% |
| VvmiR166d | T->A | 12 | 17 | 293357 | 0.01% |
| VvmiR166d | T->A | 13 | 58 | 293357 | 0.02% |
| VvmiR166d | T->A | 16 | 295 | 293357 | 0.10% |
| VvmiR166d | T->A | 17 | 20 | 293357 | 0.01% |
| VvmiR166d | T->C | 12 | 80 | 293357 | 0.03% |
| VvmiR166d | T->C | 13 | 64 | 293357 | 0.02% |
| VvmiR166d | T->C | 16 | 108 | 293357 | 0.04% |
| VvmiR166d | T->C | 17 | 222 | 293357 | 0.08% |
| VvmiR166d | T->G | 12 | 37 | 293357 | 0.01% |
| VvmiR166d | T->G | 13 | 114 | 293357 | 0.04% |
| VvmiR166d | T->G | 16 | 401 | 293357 | 0.14% |
| VvmiR166d | T->G | 17 | 6 | 293357 | 0.00% |
| VvmiR166e | A->C | 15 | 858 | 293162 | 0.29% |
| VvmiR166e | A->C | 5 | 78 | 293162 | 0.03% |
| VvmiR166e | A->C | 8 | 102 | 293162 | 0.03% |
| VvmiR166e | A->G | 15 | 484 | 293162 | 0.17% |
| VvmiR166e | A->G | 5 | 178 | 293162 | 0.06% |
| VvmiR166e | A->G | 8 | 107 | 293162 | 0.04% |
| VvmiR166e | A->T | 15 | 240 | 293162 | 0.08% |
| VvmiR166e | A->T | 5 | 42 | 293162 | 0.01% |
| VvmiR166e | A->T | 8 | 79 | 293162 | 0.03% |
| VvmiR166e | C->A | 14 | 481 | 293162 | 0.16% |
| VvmiR166e | C->A | 6 | 158 | 293162 | 0.05% |
| VvmiR166e | C->A | 7 | 43 | 293162 | 0.01% |
| VvmiR166e | C->G | 14 | 201 | 293162 | 0.07% |
| VvmiR166e | C->G | 6 | 289 | 293162 | 0.10% |
| VvmiR166e | C->G | 7 | 97 | 293162 | 0.03% |
| VvmiR166e | C->T | 14 | 423 | 293162 | 0.14% |
| VvmiR166e | C->T | 6 | 262 | 293162 | 0.09% |
| VvmiR166e | C->T | 7 | 212 | 293162 | 0.07% |
| VvmiR166e | G->A | 10 | 41 | 293162 | 0.01% |
| VvmiR166e | G->A | 9 | 30 | 293162 | 0.01% |
| VvmiR166e | G->C | 10 | 34 | 293162 | 0.01% |
| VvmiR166e | G->C | 9 | 14 | 293162 | 0.00% |
| VvmiR166e | G->T | 10 | 50 | 293162 | 0.02% |
| VvmiR166e | G->T | 9 | 113 | 293162 | 0.04% |
| VvmiR166e | T->A | 12 | 17 | 293162 | 0.01% |
| VvmiR166e | T->A | 13 | 58 | 293162 | 0.02% |
| VvmiR166e | T->A | 16 | 295 | 293162 | 0.10% |
| VvmiR166e | T->A | 17 | 20 | 293162 | 0.01% |
| VvmiR166e | T->C | 12 | 80 | 293162 | 0.03% |
| VvmiR166e | T->C | 13 | 63 | 293162 | 0.02% |
| VvmiR166e | T->C | 16 | 108 | 293162 | 0.04% |
| VvmiR166e | T->C | 17 | 221 | 293162 | 0.08% |
| VvmiR166e | T->G | 12 | 37 | 293162 | 0.01% |
| VvmiR166e | T->G | 13 | 114 | 293162 | 0.04% |
| VvmiR166e | T->G | 16 | 401 | 293162 | 0.14% |
| VvmiR166e | T->G | 17 | 6 | 293162 | 0.00% |
| VvmiR166f | A->C | 15 | 860 | 293957 | 0.29% |
| VvmiR166f | A->C | 5 | 78 | 293957 | 0.03% |
| VvmiR166f | A->C | 8 | 102 | 293957 | 0.03% |
| VvmiR166f | A->G | 15 | 490 | 293957 | 0.17% |
| VvmiR166f | A->G | 5 | 179 | 293957 | 0.06% |
| VvmiR166f | A->G | 8 | 108 | 293957 | 0.04% |
| VvmiR166f | A->T | 15 | 240 | 293957 | 0.08% |
| VvmiR166f | A->T | 5 | 42 | 293957 | 0.01% |
| VvmiR166f | A->T | 8 | 79 | 293957 | 0.03% |
| VvmiR166f | C->A | 14 | 480 | 293957 | 0.16% |
| VvmiR166f | C->A | 6 | 158 | 293957 | 0.05% |
| VvmiR166f | C->A | 7 | 44 | 293957 | 0.02% |
| VvmiR166f | C->G | 14 | 201 | 293957 | 0.07% |
| VvmiR166f | C->G | 6 | 290 | 293957 | 0.10% |
| VvmiR166f | C->G | 7 | 97 | 293957 | 0.03% |
| VvmiR166f | C->T | 14 | 425 | 293957 | 0.14% |
| VvmiR166f | C->T | 6 | 263 | 293957 | 0.09% |
| VvmiR166f | C->T | 7 | 214 | 293957 | 0.07% |
| VvmiR166f | G->A | 10 | 41 | 293957 | 0.01% |
| VvmiR166f | G->A | 9 | 30 | 293957 | 0.01% |
| VvmiR166f | G->C | 10 | 34 | 293957 | 0.01% |
| VvmiR166f | G->C | 9 | 14 | 293957 | 0.00% |
| VvmiR166f | G->T | 10 | 50 | 293957 | 0.02% |
| VvmiR166f | G->T | 9 | 113 | 293957 | 0.04% |
| VvmiR166f | T->A | 12 | 17 | 293957 | 0.01% |
| VvmiR166f | T->A | 13 | 58 | 293957 | 0.02% |
| VvmiR166f | T->A | 16 | 295 | 293957 | 0.10% |
| VvmiR166f | T->A | 17 | 20 | 293957 | 0.01% |
| VvmiR166f | T->C | 12 | 80 | 293957 | 0.03% |
| VvmiR166f | T->C | 13 | 64 | 293957 | 0.02% |
| VvmiR166f | T->C | 16 | 109 | 293957 | 0.04% |
| VvmiR166f | T->C | 17 | 222 | 293957 | 0.08% |
| VvmiR166f | T->G | 12 | 37 | 293957 | 0.01% |
| VvmiR166f | T->G | 13 | 114 | 293957 | 0.04% |
| VvmiR166f | T->G | 16 | 401 | 293957 | 0.14% |
| VvmiR166f | T->G | 17 | 6 | 293957 | 0.00% |
| VvmiR166g | A->C | 15 | 860 | 294057 | 0.29% |
| VvmiR166g | A->C | 5 | 78 | 294057 | 0.03% |
| VvmiR166g | A->C | 8 | 102 | 294057 | 0.03% |
| VvmiR166g | A->G | 15 | 490 | 294057 | 0.17% |
| VvmiR166g | A->G | 5 | 179 | 294057 | 0.06% |
| VvmiR166g | A->G | 8 | 108 | 294057 | 0.04% |
| VvmiR166g | A->T | 15 | 240 | 294057 | 0.08% |
| VvmiR166g | A->T | 5 | 43 | 294057 | 0.01% |
| VvmiR166g | A->T | 8 | 79 | 294057 | 0.03% |
| VvmiR166g | C->A | 14 | 480 | 294057 | 0.16% |
| VvmiR166g | C->A | 6 | 158 | 294057 | 0.05% |
| VvmiR166g | C->A | 7 | 44 | 294057 | 0.02% |
| VvmiR166g | C->G | 14 | 201 | 294057 | 0.07% |
| VvmiR166g | C->G | 6 | 290 | 294057 | 0.10% |
| VvmiR166g | C->G | 7 | 97 | 294057 | 0.03% |
| VvmiR166g | C->T | 14 | 425 | 294057 | 0.14% |
| VvmiR166g | C->T | 6 | 263 | 294057 | 0.09% |
| VvmiR166g | C->T | 7 | 214 | 294057 | 0.07% |
| VvmiR166g | G->A | 10 | 41 | 294057 | 0.01% |
| VvmiR166g | G->A | 9 | 30 | 294057 | 0.01% |
| VvmiR166g | G->C | 10 | 34 | 294057 | 0.01% |
| VvmiR166g | G->C | 9 | 14 | 294057 | 0.00% |
| VvmiR166g | G->T | 10 | 50 | 294057 | 0.02% |
| VvmiR166g | G->T | 9 | 113 | 294057 | 0.04% |
| VvmiR166g | T->A | 12 | 17 | 294057 | 0.01% |
| VvmiR166g | T->A | 13 | 58 | 294057 | 0.02% |
| VvmiR166g | T->A | 16 | 295 | 294057 | 0.10% |
| VvmiR166g | T->A | 17 | 20 | 294057 | 0.01% |
| VvmiR166g | T->C | 12 | 80 | 294057 | 0.03% |
| VvmiR166g | T->C | 13 | 64 | 294057 | 0.02% |
| VvmiR166g | T->C | 16 | 109 | 294057 | 0.04% |
| VvmiR166g | T->C | 17 | 222 | 294057 | 0.08% |
| VvmiR166g | T->G | 12 | 37 | 294057 | 0.01% |
| VvmiR166g | T->G | 13 | 114 | 294057 | 0.04% |
| VvmiR166g | T->G | 16 | 401 | 294057 | 0.14% |
| VvmiR166g | T->G | 17 | 6 | 294057 | 0.00% |
| VvmiR166h | A->C | 15 | 867 | 308240 | 0.28% |
| VvmiR166h | A->C | 5 | 83 | 308240 | 0.03% |
| VvmiR166h | A->C | 8 | 109 | 308240 | 0.04% |
| VvmiR166h | A->G | 15 | 489 | 308240 | 0.16% |
| VvmiR166h | A->G | 5 | 190 | 308240 | 0.06% |
| VvmiR166h | A->G | 8 | 115 | 308240 | 0.04% |
| VvmiR166h | A->T | 15 | 244 | 308240 | 0.08% |
| VvmiR166h | A->T | 5 | 45 | 308240 | 0.01% |
| VvmiR166h | A->T | 8 | 82 | 308240 | 0.03% |
| VvmiR166h | C->A | 11 | 7 | 308240 | 0.00% |
| VvmiR166h | C->A | 14 | 513 | 308240 | 0.17% |
| VvmiR166h | C->A | 6 | 164 | 308240 | 0.05% |
| VvmiR166h | C->A | 7 | 44 | 308240 | 0.01% |
| VvmiR166h | C->G | 11 | 14 | 308240 | 0.00% |
| VvmiR166h | C->G | 14 | 233 | 308240 | 0.08% |
| VvmiR166h | C->G | 6 | 292 | 308240 | 0.09% |
| VvmiR166h | C->G | 7 | 97 | 308240 | 0.03% |
| VvmiR166h | C->T | 11 | 6 | 308240 | 0.00% |
| VvmiR166h | C->T | 14 | 445 | 308240 | 0.14% |
| VvmiR166h | C->T | 6 | 271 | 308240 | 0.09% |
| VvmiR166h | C->T | 7 | 220 | 308240 | 0.07% |
| VvmiR166h | G->A | 10 | 44 | 308240 | 0.01% |
| VvmiR166h | G->A | 3 | 8 | 308240 | 0.00% |
| VvmiR166h | G->A | 4 | 8 | 308240 | 0.00% |
| VvmiR166h | G->A | 9 | 30 | 308240 | 0.01% |
| VvmiR166h | G->C | 10 | 35 | 308240 | 0.01% |
| VvmiR166h | G->C | 9 | 14 | 308240 | 0.00% |
| VvmiR166h | G->T | 10 | 56 | 308240 | 0.02% |
| VvmiR166h | G->T | 3 | 10 | 308240 | 0.00% |
| VvmiR166h | G->T | 4 | 13 | 308240 | 0.00% |
| VvmiR166h | G->T | 9 | 113 | 308240 | 0.04% |
| VvmiR166h | T->A | 12 | 24 | 308240 | 0.01% |
| VvmiR166h | T->A | 13 | 62 | 308240 | 0.02% |
| VvmiR166h | T->A | 16 | 295 | 308240 | 0.10% |
| VvmiR166h | T->A | 17 | 20 | 308240 | 0.01% |
| VvmiR166h | T->C | 12 | 95 | 308240 | 0.03% |
| VvmiR166h | T->C | 13 | 66 | 308240 | 0.02% |
| VvmiR166h | T->C | 16 | 108 | 308240 | 0.04% |
| VvmiR166h | T->C | 17 | 221 | 308240 | 0.07% |
| VvmiR166h | T->G | 12 | 40 | 308240 | 0.01% |
| VvmiR166h | T->G | 13 | 125 | 308240 | 0.04% |
| VvmiR166h | T->G | 16 | 401 | 308240 | 0.13% |
| VvmiR166h | T->G | 17 | 6 | 308240 | 0.00% |
| VvmiR167a | C->T | 10 | 9 | 360 | 2.50% |
| VvmiR167a | C->T | 9 | 10 | 360 | 2.78% |
| VvmiR167b | A->C | 14 | 7 | 9670 | 0.07% |
| VvmiR167b | A->G | 14 | 5 | 9670 | 0.05% |
| VvmiR167b | C->G | 6 | 13 | 9670 | 0.13% |
| VvmiR167b | C->T | 10 | 27 | 9670 | 0.28% |
| VvmiR167b | C->T | 6 | 5 | 9670 | 0.05% |
| VvmiR167b | C->T | 9 | 16 | 9670 | 0.17% |
| VvmiR167b | G->A | 12 | 6 | 9670 | 0.06% |
| VvmiR167b | G->A | 16 | 16 | 9670 | 0.17% |
| VvmiR167b | G->A | 8 | 6 | 9670 | 0.06% |
| VvmiR167b | G->T | 16 | 6 | 9670 | 0.06% |
| VvmiR167b | T->A | 15 | 7 | 9670 | 0.07% |
| VvmiR167b | T->G | 15 | 6 | 9670 | 0.06% |
| VvmiR167c | A->G | 14 | 5 | 6543 | 0.08% |
| VvmiR167c | C->A | 13 | 6 | 6543 | 0.09% |
| VvmiR167c | C->A | 6 | 5 | 6543 | 0.08% |
| VvmiR167c | C->G | 6 | 5 | 6543 | 0.08% |
| VvmiR167c | C->T | 10 | 15 | 6543 | 0.23% |
| VvmiR167c | C->T | 6 | 9 | 6543 | 0.14% |
| VvmiR167c | C->T | 9 | 18 | 6543 | 0.28% |
| VvmiR167c | G->A | 16 | 7 | 6543 | 0.11% |
| VvmiR167c | G->A | 5 | 5 | 6543 | 0.08% |
| VvmiR167d | C->G | 6 | 9 | 6341 | 0.14% |
| VvmiR167d | C->T | 10 | 18 | 6341 | 0.28% |
| VvmiR167d | C->T | 9 | 12 | 6341 | 0.19% |
| VvmiR167d | G->A | 16 | 12 | 6341 | 0.19% |
| VvmiR167d | T->A | 15 | 5 | 6341 | 0.08% |
| VvmiR167e | A->C | 14 | 7 | 9621 | 0.07% |
| VvmiR167e | A->G | 14 | 5 | 9621 | 0.05% |
| VvmiR167e | C->G | 6 | 13 | 9621 | 0.14% |
| VvmiR167e | C->T | 10 | 27 | 9621 | 0.28% |
| VvmiR167e | C->T | 6 | 5 | 9621 | 0.05% |
| VvmiR167e | C->T | 9 | 16 | 9621 | 0.17% |
| VvmiR167e | G->A | 12 | 6 | 9621 | 0.06% |
| VvmiR167e | G->A | 16 | 16 | 9621 | 0.17% |
| VvmiR167e | G->A | 8 | 6 | 9621 | 0.06% |
| VvmiR167e | G->T | 16 | 6 | 9621 | 0.06% |
| VvmiR167e | T->A | 15 | 7 | 9621 | 0.07% |
| VvmiR167e | T->G | 15 | 6 | 9621 | 0.06% |
| VvmiR168 | A->C | 12 | 12 | 56886 | 0.02% |
| VvmiR168 | A->G | 12 | 23 | 56886 | 0.04% |
| VvmiR168 | A->T | 12 | 6 | 56886 | 0.01% |
| VvmiR168 | C->A | 16 | 66 | 56886 | 0.12% |
| VvmiR168 | C->G | 16 | 105 | 56886 | 0.18% |
| VvmiR168 | C->T | 16 | 36 | 56886 | 0.06% |
| VvmiR168 | G->A | 10 | 16 | 56886 | 0.03% |
| VvmiR168 | G->A | 13 | 20 | 56886 | 0.04% |
| VvmiR168 | G->A | 14 | 24 | 56886 | 0.04% |
| VvmiR168 | G->A | 17 | 24 | 56886 | 0.04% |
| VvmiR168 | G->A | 7 | 9 | 56886 | 0.02% |
| VvmiR168 | G->A | 8 | 17 | 56886 | 0.03% |
| VvmiR168 | G->C | 10 | 8 | 56886 | 0.01% |
| VvmiR168 | G->C | 14 | 9 | 56886 | 0.02% |
| VvmiR168 | G->C | 17 | 6 | 56886 | 0.01% |
| VvmiR168 | G->C | 7 | 9 | 56886 | 0.02% |
| VvmiR168 | G->C | 8 | 12 | 56886 | 0.02% |
| VvmiR168 | G->T | 10 | 10 | 56886 | 0.02% |
| VvmiR168 | G->T | 13 | 13 | 56886 | 0.02% |
| VvmiR168 | G->T | 14 | 24 | 56886 | 0.04% |
| VvmiR168 | G->T | 17 | 31 | 56886 | 0.05% |
| VvmiR168 | G->T | 7 | 26 | 56886 | 0.05% |
| VvmiR168 | G->T | 8 | 41 | 56886 | 0.07% |
| VvmiR168 | T->A | 15 | 63 | 56886 | 0.11% |
| VvmiR168 | T->A | 6 | 23 | 56886 | 0.04% |
| VvmiR168 | T->A | 9 | 21 | 56886 | 0.04% |
| VvmiR168 | T->C | 15 | 40 | 56886 | 0.07% |
| VvmiR168 | T->C | 5 | 21 | 56886 | 0.04% |
| VvmiR168 | T->C | 6 | 25 | 56886 | 0.04% |
| VvmiR168 | T->C | 9 | 32 | 56886 | 0.06% |
| VvmiR168 | T->G | 15 | 139 | 56886 | 0.24% |
| VvmiR168 | T->G | 5 | 21 | 56886 | 0.04% |
| VvmiR168 | T->G | 6 | 34 | 56886 | 0.06% |
| VvmiR168 | T->G | 9 | 104 | 56886 | 0.18% |
| ***VvmiR169b*** | ***G->A*** | ***14*** | ***292*** | ***342*** | ***85.38%*** |
| ***VvmiR169f*** | ***T->C*** | ***16*** | ***27*** | ***34*** | ***79.41%*** |
| ***VvmiR169g*** | ***T->C*** | ***16*** | ***27*** | ***34*** | ***79.41%*** |
| ***VvmiR169h*** | ***G->A*** | ***14*** | ***292*** | ***341*** | ***85.63%*** |
| ***VvmiR169i*** | ***G->T*** | ***16*** | ***292*** | ***292*** | ***100.00%*** |
| VvmiR169l | A->G | 13 | 49 | 358 | 13.69% |
| VvmiR169l | C->T | 4 | 17 | 358 | 4.75% |
| ***VvmiR169r*** | ***T->C*** | ***5*** | ***269*** | ***326*** | ***82.52%*** |
| ***VvmiR169u*** | ***T->C*** | ***5*** | ***291*** | ***308*** | ***94.48%*** |
| ***VvmiR171e*** | ***C->T*** | ***12*** | ***116*** | ***117*** | ***99.15%*** |
| VvmiR172d | C->G | 8 | 5 | 2761 | 0.18% |
| VvmiR172d | C->T | 8 | 9 | 2761 | 0.33% |
| VvmiR2950 | C->T | 7 | 8 | 1781 | 0.45% |
| VvmiR2950* | C->T | 10 | 6 | 3047 | 0.20% |
| VvmiR2950* | G->T | 5 | 7 | 3047 | 0.23% |
| VvmiR2950* | T->G | 6 | 5 | 3047 | 0.16% |
| VvmiR319e | C->T | 7 | 63 | 163 | 38.65% |
| VvmiR3623 | C->G | 6 | 5 | 4423 | 0.11% |
| VvmiR3623 | C->T | 6 | 5 | 4423 | 0.11% |
| VvmiR3623 | T->G | 16 | 8 | 4423 | 0.18% |
| VvmiR3623* | A->C | 11 | 9 | 15031 | 0.06% |
| VvmiR3623* | A->C | 5 | 5 | 15031 | 0.03% |
| VvmiR3623* | A->G | 11 | 9 | 15031 | 0.06% |
| VvmiR3623* | A->G | 16 | 17 | 15031 | 0.11% |
| VvmiR3623* | A->G | 5 | 9 | 15031 | 0.06% |
| VvmiR3623* | A->G | 6 | 9 | 15031 | 0.06% |
| VvmiR3623* | A->T | 15 | 5 | 15031 | 0.03% |
| VvmiR3623* | C->A | 10 | 5 | 15031 | 0.03% |
| VvmiR3623* | C->A | 13 | 6 | 15031 | 0.04% |
| VvmiR3623* | C->A | 18 | 7 | 15031 | 0.05% |
| VvmiR3623* | C->G | 13 | 6 | 15031 | 0.04% |
| VvmiR3623* | C->G | 14 | 7 | 15031 | 0.05% |
| VvmiR3623* | C->T | 10 | 18 | 15031 | 0.12% |
| VvmiR3623* | C->T | 13 | 50 | 15031 | 0.33% |
| VvmiR3623* | C->T | 14 | 9 | 15031 | 0.06% |
| VvmiR3623* | G->T | 7 | 11 | 15031 | 0.07% |
| VvmiR3623* | T->C | 12 | 6 | 15031 | 0.04% |
| VvmiR3623* | T->C | 8 | 5 | 15031 | 0.03% |
| VvmiR3623* | T->G | 8 | 8 | 15031 | 0.05% |
| VvmiR3624 | A->G | 16 | 5 | 2965 | 0.17% |
| VvmiR3624 | G->A | 6 | 5 | 2965 | 0.17% |
| VvmiR3624 | G->T | 6 | 5 | 2965 | 0.17% |
| VvmiR3624 | G->T | 9 | 298 | 2965 | 10.05% |
| ***VvmiR3629a*** | ***G->A*** | ***8*** | ***36*** | ***63*** | ***57.14%*** |
| ***VvmiR3629b*** | ***G->A*** | ***8*** | ***34*** | ***61*** | ***55.74%*** |
| ***VvmiR3629c*** | ***G->A*** | ***8*** | ***36*** | ***63*** | ***57.14%*** |
| ***VvmiR3631b**** | ***A->G*** | ***10*** | ***83*** | ***131*** | ***63.36%*** |
| VvmiR3631b* | T->C | 17 | 20 | 131 | 15.27% |
| VvmiR3633a | A->G | 14 | 6 | 6190 | 0.10% |
| VvmiR3633a | G->A | 6 | 6 | 6190 | 0.10% |
| VvmiR3633a | G->C | 6 | 6 | 6190 | 0.10% |
| VvmiR3633a | T->G | 12 | 8 | 6190 | 0.13% |
| VvmiR3635 | C->A | 14 | 5 | 3026 | 0.17% |
| VvmiR3636 | A->G | 12 | 6 | 13141 | 0.05% |
| VvmiR3636 | A->G | 13 | 9 | 13141 | 0.07% |
| VvmiR3636 | A->G | 16 | 15 | 13141 | 0.11% |
| VvmiR3636 | A->T | 12 | 5 | 13141 | 0.04% |
| VvmiR3636 | C->A | 15 | 22 | 13141 | 0.17% |
| VvmiR3636 | C->G | 20 | 5 | 13141 | 0.04% |
| VvmiR3636 | C->G | 7 | 7 | 13141 | 0.05% |
| VvmiR3636 | C->T | 7 | 8 | 13141 | 0.06% |
| VvmiR3636 | G->A | 11 | 5 | 13141 | 0.04% |
| VvmiR3636 | G->A | 14 | 10 | 13141 | 0.08% |
| VvmiR3636 | G->A | 5 | 8 | 13141 | 0.06% |
| VvmiR3636 | G->A | 8 | 8 | 13141 | 0.06% |
| VvmiR3636 | G->C | 14 | 8 | 13141 | 0.06% |
| VvmiR3636 | G->T | 11 | 6 | 13141 | 0.05% |
| VvmiR3636 | G->T | 14 | 8 | 13141 | 0.06% |
| VvmiR3636 | G->T | 18 | 6 | 13141 | 0.05% |
| VvmiR3636 | G->T | 5 | 8 | 13141 | 0.06% |
| VvmiR3636 | G->T | 8 | 5 | 13141 | 0.04% |
| VvmiR3636 | G->T | 9 | 10 | 13141 | 0.08% |
| VvmiR3636 | T->A | 19 | 6 | 13141 | 0.05% |
| VvmiR3636 | T->A | 6 | 5 | 13141 | 0.04% |
| VvmiR3636 | T->C | 19 | 5 | 13141 | 0.04% |
| VvmiR3636 | T->C | 6 | 6 | 13141 | 0.05% |
| VvmiR3636 | T->G | 19 | 8 | 13141 | 0.06% |
| VvmiR3636 | T->G | 6 | 6 | 13141 | 0.05% |
| VvmiR3639 | G->A | 15 | 9 | 502 | 1.79% |
| VvmiR396a | A->G | 7 | 708 | 1461 | 48.46% |
| ***VvmiR396b*** | ***A->G*** | ***7*** | ***1163*** | ***2171*** | ***53.57%*** |
| ***VvmiR396c*** | ***A->G*** | ***7*** | ***614*** | ***871*** | ***70.49%*** |
| ***VvmiR396d*** | ***A->G*** | ***7*** | ***614*** | ***876*** | ***70.09%*** |
| VvmiR399a | A->G | 13 | 5 | 15 | 33.33% |
| ***VvmiR399b*** | ***G->A*** | ***13*** | ***10*** | ***15*** | ***66.67%*** |
| ***VvmiR399c*** | ***G->A*** | ***13*** | ***10*** | ***15*** | ***66.67%*** |
| VvmiR399h | A->G | 13 | 5 | 15 | 33.33% |
| VvmiR479 | A->G | 7 | 10 | 35202 | 0.03% |
| VvmiR479 | A->T | 7 | 6 | 35202 | 0.02% |
| VvmiR479 | C->A | 14 | 13 | 35202 | 0.04% |
| VvmiR479 | C->A | 17 | 9 | 35202 | 0.03% |
| VvmiR479 | C->G | 14 | 17 | 35202 | 0.05% |
| VvmiR479 | C->G | 17 | 8 | 35202 | 0.02% |
| VvmiR479 | C->T | 14 | 12 | 35202 | 0.03% |
| VvmiR479 | C->T | 17 | 12 | 35202 | 0.03% |
| VvmiR479 | G->A | 10 | 7 | 35202 | 0.02% |
| VvmiR479 | G->A | 11 | 10 | 35202 | 0.03% |
| VvmiR479 | G->A | 15 | 18 | 35202 | 0.05% |
| VvmiR479 | G->A | 16 | 37 | 35202 | 0.11% |
| VvmiR479 | G->A | 5 | 10 | 35202 | 0.03% |
| VvmiR479 | G->C | 11 | 7 | 35202 | 0.02% |
| VvmiR479 | G->C | 16 | 6 | 35202 | 0.02% |
| VvmiR479 | G->C | 5 | 7 | 35202 | 0.02% |
| VvmiR479 | G->T | 10 | 21 | 35202 | 0.06% |
| VvmiR479 | G->T | 11 | 35 | 35202 | 0.10% |
| VvmiR479 | G->T | 15 | 9 | 35202 | 0.03% |
| VvmiR479 | G->T | 16 | 10 | 35202 | 0.03% |
| VvmiR479 | G->T | 5 | 10 | 35202 | 0.03% |
| VvmiR479 | T->A | 6 | 36 | 35202 | 0.10% |
| VvmiR479 | T->C | 12 | 9 | 35202 | 0.03% |
| VvmiR479 | T->C | 13 | 7 | 35202 | 0.02% |
| VvmiR479 | T->C | 18 | 8 | 35202 | 0.02% |
| VvmiR479 | T->C | 6 | 19 | 35202 | 0.05% |
| VvmiR479 | T->C | 8 | 11 | 35202 | 0.03% |
| VvmiR479 | T->C | 9 | 6 | 35202 | 0.02% |
| VvmiR479 | T->G | 12 | 12 | 35202 | 0.03% |
| VvmiR479 | T->G | 13 | 14 | 35202 | 0.04% |
| VvmiR479 | T->G | 6 | 69 | 35202 | 0.20% |
| VvmiR535a | A->G | 15 | 6 | 3444 | 0.17% |
| VvmiR535a | A->G | 5 | 6 | 3444 | 0.17% |
| VvmiR535a | C->A | 7 | 5 | 3444 | 0.15% |
| VvmiR535b | A->G | 15 | 6 | 3444 | 0.17% |
| VvmiR535b | A->G | 5 | 6 | 3444 | 0.17% |
| VvmiR535b | C->A | 7 | 5 | 3444 | 0.15% |
| VvmiR535c | A->G | 15 | 6 | 3444 | 0.17% |
| VvmiR535c | A->G | 5 | 6 | 3444 | 0.17% |
| VvmiR535c | C->A | 7 | 5 | 3444 | 0.15% |
| ***VvmiR828b*** | ***G->A*** | ***17*** | ***7*** | ***7*** | ***100.00%*** |

Notes: Words with italic denote VvmiRNA edit types with high variation frequency

**Table S6** List of pathways invloved by target genes for VvmiRNAs

| Pathway | Gene Number | pvalue | qvalue | Pathway ID | Genes |  |
| --- | --- | --- | --- | --- | --- | --- |
| Plant-pathogen interaction | 45 | 1.30E-20 | 9.91E-19 | ko04626 | VIT_10s0116g00910.t01;VIT_11s0037g01270.t01;VIT_12s0035g01260.t01;VIT_12s0035g01330.t01;VIT_15s0045g00680.t01;VIT_15s0045g00880.t01;VIT_15s0045g00980.t01;VIT_15s0045g01020.t01;VIT_15s0046g02810.t01;VIT_15s0046g03660.t01;VIT_16s0022g01330.t01;VIT_19s0014g00580.t01;VIT_19s0014g05140.t01;VIT_19s0014g05150.t01;VIT_19s0014g05160.t01;VIT_19s0014g05180.t01;VIT_19s0093g00380.t01;VIT_03s0038g01520.t01;VIT_03s0038g01540.t01;VIT_03s0038g01550.t01;VIT_03s0038g01610.t01;VIT_03s0038g01620.t01;VIT_03s0038g01630.t01;VIT_03s0038g01670.t01;VIT_06s0009g01660.t01;VIT_07s0005g06180.t01;VIT_07s0005g06200.t01;VIT_07s0005g06210.t01;VIT_07s0005g06220.t01;VIT_07s0005g06240.t01;VIT_09s0002g03790.t01;VIT_09s0002g03980.t01;VIT_09s0002g04890.t01;VIT_09s0002g04910.t01;VIT_09s0002g04940.t01;VIT_09s0002g04950.t01;VIT_09s0002g05040.t01;VIT_09s0002g05050.t01;VIT_09s0002g05070.t01;VIT_09s0002g05220.t01;VIT_09s0002g06260.t01;VIT_09s0070g00350.t01;VIT_09s0096g00830.t01;VIT_09s0002g00240.t01;VIT_00s0515g00020.t01 |  |
| Pentose and glucuronate interconversions | 11 | 0.000568179 | 2.16E-02 | ko00040 | VIT_11s0052g01710.t01;VIT_12s0057g00370.t01;VIT_12s0057g00380.t01;VIT_13s0047g00230.t01;VIT_14s0219g00230.t01;VIT_17s0000g09810.t01;VIT_18s0001g01640.t01;VIT_08s0007g07750.t01;VIT_08s0007g07760.t01;VIT_08s0007g07770.t01;VIT_08s0007g07780.t01 |  |
| Base excision repair | 4 | 0.03662017 | 8.76E-01 | ko03410 | VIT_03s0063g02660.t01;VIT_04s0023g03440.t01;VIT_07s0005g00630.t01;VIT_07s0005g03040.t01 |  |
| Taurine and hypotaurine metabolism | 2 | 0.04609587 | 8.76E-01 | ko00430 | VIT_01s0011g06600.t01;VIT_17s0000g00920.t01 |  |
| C5-Branched dibasic acid metabolism | 2 | 0.06731192 | 9.93E-01 | ko00660 | VIT_05s0049g01980.t01;VIT_08s0056g01640.t01 |  |
| RNA degradation | 7 | 0.08464915 | 9.93E-01 | ko03018 | VIT_15s0021g02580.t01;VIT_03s0017g00990.t01;VIT_04s0008g05850.t01;VIT_04s0008g06260.t01;VIT_04s0043g00400.t01;VIT_06s0004g05980.t01;VIT_00s0455g00020.t01 |  |
| Regulation of autophagy | 3 | 0.0914372 | 9.93E-01 | ko04140 | VIT_01s0127g00430.t01;VIT_16s0098g00590.t01;VIT_08s0040g00720.t01 |  |
| Selenocompound metabolism | 2 | 0.1168654 | 9.97E-01 | ko00450 | VIT_05s0020g04210.t01;VIT_08s0007g08760.t01 |  |
| Caffeine metabolism | 1 | 0.1273584 | 9.97E-01 | ko00232 | VIT_17s0000g03930.t01 |  |
| Stilbenoid, diarylheptanoid and gingerol biosynthesis | 3 | 0.1502166 | 9.97E-01 | ko00945 | VIT_12s0028g01880.t01;VIT_12s0028g01940.t01;VIT_12s0028g03110.t01 |  |
| Protein processing in endoplasmic reticulum | 10 | 0.1952419 | 9.97E-01 | ko04141 | VIT_01s0011g03410.t01;VIT_10s0003g03950.t01;VIT_13s0019g03050.t01;VIT_13s0067g01710.t01;VIT_04s0043g00710.t01;VIT_07s0031g00230.t01;VIT_07s0031g02660.t01;VIT_08s0032g00940.t01;VIT_09s0002g04250.t01;VIT_09s0096g00090.t01 |  |
| Valine, leucine and isoleucine biosynthesis | 2 | 0.2219198 | 9.97E-01 | ko00290 | VIT_05s0049g01980.t01;VIT_08s0056g01640.t01 |  |
| RNA transport | 8 | 0.2503203 | 9.97E-01 | ko03013 | VIT_12s0059g02370.t01;VIT_13s0067g01710.t01;VIT_14s0128g00480.t01;VIT_18s0001g10440.t01;VIT_04s0043g00740.t01;VIT_06s0004g00080.t01;VIT_06s0004g00090.t01;VIT_08s0007g08500.t01 |  |
| Monobactam biosynthesis | 1 | 0.2590739 | 9.97E-01 | ko00261 | VIT_05s0020g04210.t01 |  |
| Butanoate metabolism | 2 | 0.3320414 | 9.97E-01 | ko00650 | VIT_01s0011g06600.t01;VIT_17s0000g00920.t01 |  |
| Nicotinate and nicotinamide metabolism | 1 | 0.3357036 | 9.97E-01 | ko00760 | VIT_01s0011g04800.t01 |  |
| Pyrimidine metabolism | 5 | 0.3556393 | 9.97E-01 | ko00240 | VIT_01s0026g02490.t01;VIT_13s0084g00170.t01;VIT_04s0023g01440.t01;VIT_05s0051g00930.t01;VIT_08s0007g08760.t01 |  |
| Nucleotide excision repair | 3 | 0.3718613 | 9.97E-01 | ko03420 | VIT_01s0011g03410.t01;VIT_15s0045g00530.t01;VIT_00s0591g00010.t01 |  |
| Ascorbate and aldarate metabolism | 2 | 0.4001397 | 9.97E-01 | ko00053 | VIT_10s0003g05000.t01;VIT_18s0001g01640.t01 |  |
| 2-Oxocarboxylic acid metabolism | 3 | 0.4229375 | 9.97E-01 | ko01210 | VIT_12s0142g00610.t01;VIT_05s0049g01980.t01;VIT_08s0056g01640.t01 |  |
| Folate biosynthesis | 1 | 0.4361236 | 9.97E-01 | ko00790 | VIT_13s0019g04180.t01 |  |
| Alanine, aspartate and glutamate metabolism | 2 | 0.4467772 | 9.97E-01 | ko00250 | VIT_01s0011g06600.t01;VIT_17s0000g00920.t01 |  |
| Starch and sucrose metabolism | 9 | 0.455052 | 9.97E-01 | ko00500 | VIT_12s0057g00370.t01;VIT_12s0057g00380.t01;VIT_13s0047g00230.t01;VIT_05s0020g00510.t01;VIT_06s0004g06110.t01;VIT_08s0007g07750.t01;VIT_08s0007g07760.t01;VIT_08s0007g07770.t01;VIT_08s0007g07780.t01 |  |
| Linoleic acid metabolism | 1 | 0.4661217 | 9.97E-01 | ko00591 | VIT_17s0000g03930.t01 |  |
| RNA polymerase | 2 | 0.4826051 | 9.97E-01 | ko03020 | VIT_13s0084g00170.t01;VIT_04s0023g01440.t01 |  |
| Cyanoamino acid metabolism | 3 | 0.4863977 | 9.97E-01 | ko00460 | VIT_06s0004g06110.t01;VIT_06s0004g06480.t01;VIT_06s0080g00940.t01 |  |
| Mismatch repair | 2 | 0.4999802 | 9.97E-01 | ko03430 | VIT_15s0045g00530.t01;VIT_17s0000g02690.t01 |  |
| Other glycan degradation | 1 | 0.5214409 | 9.97E-01 | ko00511 | VIT_06s0061g00480.t01 |  |
| Citrate cycle (TCA cycle) | 2 | 0.5335954 | 9.97E-01 | ko00020 | VIT_10s0042g00950.t01;VIT_12s0142g00610.t01 |  |
| beta-Alanine metabolism | 2 | 0.5417566 | 9.97E-01 | ko00410 | VIT_01s0011g06600.t01;VIT_17s0000g00920.t01 |  |
| Phenylalanine, tyrosine and tryptophan biosynthesis | 2 | 0.549819 | 9.97E-01 | ko00400 | VIT_14s0066g00970.t01;VIT_09s0002g08060.t01 |  |
| Terpenoid backbone biosynthesis | 2 | 0.5577822 | 9.97E-01 | ko00900 | VIT_06s0004g06980.t01;VIT_06s0004g06990.t01 |  |
| DNA replication | 2 | 0.5577822 | 9.97E-01 | ko03030 | VIT_15s0045g00530.t01;VIT_05s0051g00930.t01 |  |
| Circadian rhythm - plant | 2 | 0.5577822 | 9.97E-01 | ko04712 | VIT_14s0006g01340.t01;VIT_17s0000g06570.t01 |  |
| Purine metabolism | 5 | 0.5737726 | 9.97E-01 | ko00230 | VIT_01s0026g02490.t01;VIT_13s0084g00170.t01;VIT_04s0023g01440.t01;VIT_05s0020g04210.t01;VIT_05s0051g00930.t01 |  |
| Homologous recombination | 2 | 0.5886331 | 9.97E-01 | ko03440 | VIT_04s0044g01570.t01;VIT_05s0062g00860.t01 |  |
| Arginine and proline metabolism | 2 | 0.5886331 | 9.97E-01 | ko00330 | VIT_14s0083g00520.t01;VIT_05s0020g02220.t01 |  |
| Phenylpropanoid biosynthesis | 6 | 0.5972976 | 9.97E-01 | ko00940 | VIT_01s0010g03720.t01;VIT_10s0003g04160.t01;VIT_12s0028g03110.t01;VIT_05s0020g02110.t01;VIT_06s0004g06110.t01;VIT_08s0040g02200.t01 |  |
| ABC transporters | 1 | 0.625949 | 9.97E-01 | ko02010 | VIT_10s0003g02540.t01 |  |
| Cutin, suberine and wax biosynthesis | 1 | 0.625949 | 9.97E-01 | ko00073 | VIT_17s0000g00950.t01 |  |
| Diterpenoid biosynthesis | 1 | 0.6360585 | 9.97E-01 | ko00904 | VIT_09s0002g05300.t01 |  |
| Sesquiterpenoid and triterpenoid biosynthesis | 2 | 0.6454973 | 9.97E-01 | ko00909 | VIT_10s0003g03540.t01;VIT_18s0001g04510.t01 |  |
| Galactose metabolism | 2 | 0.6454973 | 9.97E-01 | ko00052 | VIT_18s0001g01640.t01;VIT_05s0077g00840.t01 |  |
| Sulfur metabolism | 1 | 0.6458963 | 9.97E-01 | ko00920 | VIT_05s0020g04210.t01 |  |
| Cysteine and methionine metabolism | 3 | 0.6531771 | 9.97E-01 | ko00270 | VIT_11s0016g02560.t01;VIT_12s0035g01770.t01;VIT_16s0039g02460.t01 |  |
| Phenylalanine metabolism | 2 | 0.6900326 | 9.97E-01 | ko00360 | VIT_01s0010g03720.t01;VIT_12s0028g03110.t01 |  |
| Lysine degradation | 1 | 0.6912585 | 9.97E-01 | ko00310 | VIT_16s0039g00740.t01 |  |
| Aminoacyl-tRNA biosynthesis | 2 | 0.7076731 | 9.97E-01 | ko00970 | VIT_12s0057g00400.t01;VIT_16s0100g00370.t01 |  |
| N-Glycan biosynthesis | 1 | 0.7452215 | 9.97E-01 | ko00510 | VIT_14s0066g02390.t01 |  |
| Porphyrin and chlorophyll metabolism | 1 | 0.7588389 | 9.97E-01 | ko00860 | VIT_01s0010g00590.t01 |  |
| Ribosome biogenesis in eukaryotes | 2 | 0.7653446 | 9.97E-01 | ko03008 | VIT_07s0031g01750.t01;VIT_08s0007g08500.t01 |  |
| Protein export | 1 | 0.7653738 | 9.97E-01 | ko03060 | VIT_15s0048g02080.t01 |  |
| Tryptophan metabolism | 1 | 0.7653738 | 9.97E-01 | ko00380 | VIT_17s0000g03930.t01 |  |
| Propanoate metabolism | 1 | 0.7897995 | 9.97E-01 | ko00640 | VIT_10s0042g00950.t01 |  |
| Inositol phosphate metabolism | 1 | 0.7897995 | 9.97E-01 | ko00562 | VIT_01s0127g00430.t01 |  |
| mRNA surveillance pathway | 3 | 0.7962919 | 9.97E-01 | ko03015 | VIT_18s0001g01800.t01;VIT_04s0008g00840.t01;VIT_05s0049g01660.t01 |  |
| Tropane, piperidine and pyridine alkaloid biosynthesis | 1 | 0.8064443 | 9.97E-01 | ko00960 | VIT_06s0004g05320.t01 |  |
| Ubiquinone and other terpenoid-quinone biosynthesis | 1 | 0.8266164 | 9.97E-01 | ko00130 | VIT_01s0010g03720.t01 |  |
| Insulin resistance | 1 | 0.8403612 | 9.97E-01 | ko04931 | VIT_08s0040g00720.t01 |  |
| Photosynthesis | 1 | 0.848917 | 9.97E-01 | ko00195 | VIT_19s0014g05080.t01 |  |
| Fructose and mannose metabolism | 1 | 0.8530224 | 9.97E-01 | ko00051 | VIT_11s0052g01710.t01 |  |
| Phosphatidylinositol signaling system | 1 | 0.8683643 | 9.97E-01 | ko04070 | VIT_01s0127g00430.t01 |  |
| Amino sugar and nucleotide sugar metabolism | 3 | 0.8909993 | 9.97E-01 | ko00520 | VIT_15s0046g01720.t01;VIT_18s0001g01640.t01;VIT_05s0020g00510.t01 |  |
| Biosynthesis of amino acids | 5 | 0.9032105 | 9.97E-01 | ko01230 | VIT_12s0142g00610.t01;VIT_14s0066g00970.t01;VIT_05s0049g01980.t01;VIT_08s0056g01640.t01;VIT_09s0002g08060.t01 |  |
| Carbon fixation in photosynthetic organisms | 1 | 0.9054742 | 9.97E-01 | ko00710 | VIT_02s0109g00080.t01 |  |
| Flavonoid biosynthesis | 1 | 0.9080499 | 9.97E-01 | ko00941 | VIT_12s0028g03110.t01 |  |
| Plant hormone signal transduction | 5 | 0.9108258 | 9.97E-01 | ko04075 | VIT_01s0011g05260.t01;VIT_01s0011g05560.t01;VIT_15s0046g00290.t01;VIT_03s0038g01150.t01;VIT_07s0104g00930.t01 |  |
| Glyoxylate and dicarboxylate metabolism | 1 | 0.9199187 | 9.97E-01 | ko00630 | VIT_12s0142g00610.t01 |  |
| Glutathione metabolism | 2 | 0.9233503 | 9.97E-01 | ko00480 | VIT_18s0072g00320.t01;VIT_07s0104g01830.t01 |  |
| Glycerophospholipid metabolism | 1 | 0.9539802 | 9.97E-01 | ko00564 | VIT_10s0116g00860.t01 |  |
| Oxidative phosphorylation | 2 | 0.9681027 | 9.97E-01 | ko00190 | VIT_01s0011g01030.t01;VIT_02s0012g01500.t01 |  |
| Phagosome | 1 | 0.9696627 | 9.97E-01 | ko04145 | VIT_01s0127g00430.t01 |  |
| Carbon metabolism | 3 | 0.9947465 | 9.97E-01 | ko01200 | VIT_10s0042g00950.t01;VIT_12s0142g00610.t01;VIT_02s0109g00080.t01 |  |
| Endocytosis | 1 | 0.9955983 | 9.97E-01 | ko04144 | VIT_10s0003g01610.t01 |  |
| Ribosome | 3 | 0.9962036 | 9.97E-01 | ko03010 | VIT_12s0035g01020.t01;VIT_14s0060g02050.t01;VIT_06s0080g00400.t01 |  |
| Spliceosome | 1 | 0.9973496 | 9.97E-01 | ko03040 | VIT_06s0004g08160.t01 |  |

**Table S7** List of miRNA specific primers for miR-RACE

| miRNA name | MGSP1 | MGSP2 |
| --- | --- | --- |
| VvmiR8-5p | GGAGTAGAAATCCAAGGATGGAAAAGG | TTTTTTTTTTGAAGCCTTTTCCATCCT |
| VvmiR16-5p | GGAGTAGAAATTTCTTAGCAACCAAAC | TTTTTTTTTTAGGCCTTCTATCAAGAA |
| VvmiR31-3p | GGAGTAGAAAGAAGCTCTTGAGGGGGA | TTTTTTTTTTCTCTGTTTGGTTGCTAA |
| VvmiR38-3p | GGAGTAGAAAAGGTGCAGGTGAAGGTG | TTTTTTTTTTCAGTCCCCCTCAAGAGC |
| VvmiR44-3p | GGAGTAGAAAAGGTGCAGGTGAAGGTG | TTTTTTTTTTTCTGCACCTTCACCTGC |
| VvmiR53-3p | GGAGTAGAAAGGCAGCAGCATACTACT | TTTTTTTTTTCAAAGTAGTATGCTGCT |

**Table S8** List of primers for RLM-RACE and PPM-RACE

| miRNA name | P1 | P2 |
| --- | --- | --- |
| VvmiR8-5p | TTGCTGATTGCTGGAGATTG | CAGGGCGTGCAAGTACATTA |
| VvmiR16-5p | CCAACCATGAAAATCCATCC | GCATGGACAAGGGAAGAAGA |
| VvmiR31-3p | ACCAACCGGGACTTGTGATA | GGAGCCACAACCTCTCAAAG |
| VvmiR38-3p | AATGCATCCAACAGACCACA | GCTTGAGAAGGTCGGTGAAG |
| VvmiR44-3p | AGCTCTGAGTCTCCCCACAA | CCTAAACTTCCGATCGTCCA |
| VvmiR53-3p | GAAGCCCCGTGTTGAATAAA | TTGTTCACAGGCACAAGAGC |

**Table S9** List of primers for qRT-PCR

| miRNA name | qP1 | qP2 |
| --- | --- | --- |
| VvmiR8-5p | ATTCTAGAGGCCGAGGCGGCCGACATG | TCCAAGGATGGAAAAGGCTTC |
| VvmiR16-5p | ATTCTAGAGGCCGAGGCGGCCGACATG | TCTTTTCTTGATAGAAGGCCT |
| VvmiR31-3p | ATTCTAGAGGCCGAGGCGGCCGACATG | TTTCTTAGCAACCAAACAGAG |
| VvmiR38-3p | ATTCTAGAGGCCGAGGCGGCCGACATG | GAAGCTCTTGAGGGGGACTG |
| VvmiR44-3p | ATTCTAGAGGCCGAGGCGGCCGACATG | AGGTGCAGGTGAAGGTGCAGA |
| VvmiR53-3p | ATTCTAGAGGCCGAGGCGGCCGACATG | GGCAGCAGCATACTACTTTG |
